# Skeletal Editing of Alkenes with Nitroarenes via Photoinduced Rearrangement of N─O═C Dipoles Forms Lactams and Amides

**DOI:** 10.1002/advs.202518828

**Published:** 2025-11-23

**Authors:** Hongyun Qin, Zemin Wang, Cong Shi, Jiashu Chen, Chao Liu, Xiangqian Li, Ruihua Liu, Dayong Shi

**Affiliations:** ^1^ State Key Laboratory of Microbial Technology Shandong University Qingdao Shandong 266237 P. R. China; ^2^ Shenzhen Research Institute of Shandong University Shenzhen Guangdong 518057 P. R. China; ^3^ Laboratory for Marine Drugs and Bioproducts Qingdao Marine Science and Technology Center Qingdao Shandong 266237 P. R. China

**Keywords:** lactams and amides, N─C═O dipoles, nitrogen atom insertion, rearrangement, skeletal editing

## Abstract

Photoinduced oxidative cleavage of alkenes with nitroarenes has proven to be an efficient approach for generating N─O = C dipoles. These dipoles exhibit diverse reactivity profiles, making the rearrangement of N─O═C dipoles a promising strategy for achieving nitrogen atom insertion in skeletal editing. Herein, we present a novel protocol for the synthesis of diverse lactam and amide derivatives through skeletal editing of alkenes using nitroarenes as nitrogen atom insertion reagents under light irradiation, offering the advantages of operational simplicity, high efficiency, and sustainability. Mechanistic studies and DFT calculations indicate that it proceeds through an intramolecular synergistic cyclization‐cleavage‐rearrangement process involving N─O═C dipoles, which realizes the cascade cleavage of C(sp^2^)═C(sp^2^) and C(sp^3^)─C(sp^3^) bonds and the insertion of the nitrogen atom, differing from previous understanding. This exploration highlights an emerging frontier in the application of N─O═C dipoles, offering potential for the development of novel skeletal editing strategies.

## Graphical abstract

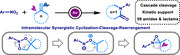

## 1 Introduction

**Scheme 1:**
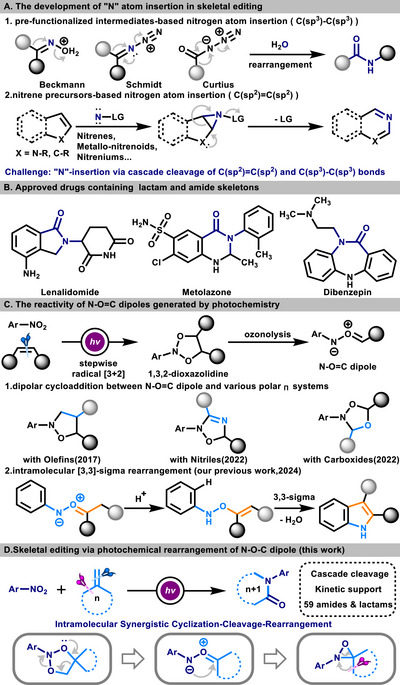
A) The nitrogen atom insertion of novel pathways can enable the skeletal editing of more N‐heterocyclic structures; B) Lactams and amides are common drug scaffolds; C) The development and utilization of N─O═C dipoles generated from oxidative cleavage of alkenes with nitroarenes; D) Skeletal editing of alkenes with nitroarenes via photoinduced rearrangement of N─O═C dipoles forms lactams and amides (this work).

N‐heterocyclic scaffolds represent a fundamental structural motif in medicinal chemistry, and the development of synthetic strategies to address the demands of varied chemical contexts remains of practical significance. In recent years, skeletal editing involving nitrogen atom insertion has emerged as an essential strategy for the construction of N‐heterocycles. As research progresses, substantial advancements have been achieved in the expansion of reaction pathways for nitrogen atom insertion. From the perspective of mechanism, it can be divided into two major categories. One is that the nitrogen atom insertion is achieved through the rearrangement of pre‐functionalized intermediates (similar to the Ritter intermediate).^[^ ^1^, ^2^, ^3^, ^4^, ^5^ ^]^ The other is that the widely developed insertion strategies based on nitrene precursors (such as nitrenes, metallo‐nitrenoids, and nitreniums) can achieve the insertion of highly active nitrene species through a concerted process (**Scheme** **1** **A**).^[^ ^6^, ^7^, ^8^, ^9^, ^10^, ^11^, ^12^, ^13^, ^14^, ^15^ ^]^ Compared with the nitrene strategy targeting the C(sp^2^)═C(sp^2^) bond (such as arenes and alkenes) for the synthesis of azaarenes, the rearrangement of specific intermediates has an irreplaceable advantage in achieving the insertion of nitrogen atom into the C(sp^3^)─C(sp^3^) bond to construct specific N‐heterocyclic scaffolds (such as amide motifs). Lactams and amides represent fundamental N‐heterocycles in pharmaceutical chemistry, and their synthetic methodologies are well established (Scheme 1B).^[^ ^16^, ^17^, ^18^, ^19^, ^20^, ^21^ ^]^ Meanwhile, transition metal‐catalyzed amine carbonylation,^[^ ^22^, ^23^, ^24^, ^25^, ^26^, ^27^ ^]^ oxidation of aldimines,^[^ ^28^, ^29^ ^]^ Beckmann/Schmidt rearrangements,^[^ ^30^, ^31^, ^32^, ^33^ ^]^ Passerini/Ugi reactions,^[^ ^34^, ^35^, ^36^ ^]^ and enzymatic catalysis have demonstrated unique value in the synthesis of amides with specific structures from designated starting materials.^[^ ^37^, ^38^, ^39^ ^]^ Therefore, developing efficient and environmentally friendly strategies that enable the cascade cleavage of C(sp^2^)═C(sp^2^) and C(sp^3^)─C(sp^3^) bonds through the use of well‐designed intermediates, and thereby facilitate the insertion of nitrogen atoms to construct structurally diverse amide motifs, represents a promising research direction.

Meanwhile, the re‐examination of the properties of nitroarenes in photochemistry have led to their widespread application as multifunctional materials in the development of various synthetic strategies,^[^ ^40^, ^41^ ^]^ which include hydrogen atom transfer (HAT),^[^ ^42^, ^43^, ^44^, ^45^, ^46^ ^]^ oxygen atom transfer (OAT) through dipolar cycloadditions analogous to ozonolysis,^[^ ^47^, ^48^, ^49^, ^50^, ^51^, ^52^, ^53^, ^54^, ^55^, ^56^ ^]^ electron donor–acceptor (EDA) complexes,^[^ ^57^, ^58^, ^59^ ^]^ and other novel strategies.^[^ ^60^, ^61^, ^62^, ^63^, ^64^, ^65^, ^66^ ^]^ The photoexcited nitro‐driven synthesis, due to its favorable characteristics such as mild conditions and easy availability of substrates, has been widely applied in the transformation of various unsaturated structures such as alkenes, alkynes, imines, and aromatic rings.^[^ ^67^, ^68^, ^69^ ^]^ Notably, the oxidative cleavage of alkenes with nitroarenes has emerged as an efficient method for synthesizing carbonyl compounds.^[^ ^48^, ^49^, ^50^ ^]^ This strategy, grounded in the secondary cycloaddition‐cleavage mechanism of N─O═C dipoles, underscores the synthetic versatility of N─O═C dipoles in organic chemistry. Within the dipole chemistry, the utilization of N─O═C dipoles remains comparatively limited relative to other dipole types. At present, their applications are confined to 3+2 cycloadditions with polar π systems,^[^ ^70^, ^71^ ^]^ and a photoinduced Bartoli indole synthesis was reported in 2024, which achieves the synthesis of indoles and azaindoles through the ene‐rearrangement of the N─O═C dipole.^[^ ^72^ ^]^ Therefore, the N─O═C dipole generated from photoexcited nitro can undergo rearrangement driven by its charge distribution and the stability of the compound, forming a more stable system, and this property shows application potential in the construction of N‐heterocycles (Scheme 1C).^[^ ^73^ ^]^

Based on the above studies and theoretical feasibility, a method has been developed for skeletal editing of alkenes using nitroarenes as nitrogen atom insertion reagents to construct lactam and amide derivatives. This process involves the radical addition of photoexcited nitroarenes to alkenes and an intramolecular synergetic cyclization‐cleavage‐rearrangement of the N─O═C dipole intermediate, which realizes the cascade cleavage of C(sp^2^)═C(sp^2^) and C(sp^3^)─C(sp^3^) bonds and the insertion of the nitrogen atom. It is supported by experiments and DFT calculations. Compared with previous methods, this approach is more environmentally friendly and efficient. The skeletal editing of various olefins demonstrates its potential in drug synthesis, making it an indispensable strategy for the synthesis of amide structural motifs. Overall, this method expands the reactivity of the N─O═C dipole, representing a novel approach to skeletal editing and enriching the connotations of excited‐state nitro synthesis and dipole chemistry (Scheme 1D).

## 2 Results and Discussion

### 2.1 Optimization of the Conditions

Photoinduced oxidative cleavage of alkenes using nitroarenes has emerged as a well‐established approach for the synthesis of carbonyl compounds,^[^ ^48^, ^49^, ^50^ ^]^ and amides are formed as by‐products in this process. However, their formation mechanisms and potential applications remain largely unexplored. Given the practical value of amides and lactams, the factors that influence their reactivity transformation were systematically investigated and optimized. We initially selected 5‐methylene‐6,7‐dihydro‐5H‐cyclopenta[b]pyridine (**1o**) and 1,3,5‐trifluoro‐2‐nitrobenzene (**2r**) as model substrates for optimization. Encouragingly, irradiation. with a 24 W, 390 nm light source in MeCN (0.04 M) at room temperature under an air atmosphere afforded lactam **3o** in 66% yield, accompanied by 20% ketone by‐product (**Table** **1** Entry 1). These results indicate that, although further optimization is possible, this system offers a promising platform for constructing lactam skeletons. Subsequent experiments confirmed that light irradiation is essential for the reaction (Table 1 Entry 2). The yield decreased significantly as the wavelength increased, due to the specific excitation characteristics of nitroarenes (Table 1 Entry 3, 4). Regarding solvents, ethyl acetate showed moderate performance, while no trace of product was obtained in dichloromethane. In contrast, other nitrile solvents consistently demonstrated stable reactivity (Table 1 Entry 5–7). Given the potential for an intramolecular rearrangement, the impact of concentration was evaluated. And reduced concentrations were found to slightly enhance the yield (Table 1 Entry 8, 9). Employing photoreactors with identical wavelengths but varying power levels had a notable influence on both yield and selectivity: the 12 W lamp yielded less product, while the 50 W lamp produced a marginally lower yield (Table 1 Entry 11, 12). These effects are associated with photon utilization efficiency, and proper photon utilization facilitates the regulation of intermediate concentrations and system energy levels, thereby modulating reactivity and overall yield.^[^ ^74^ ^]^

**Table 1:** Optimization Studies for the Synthesis of **3o**.^a)^

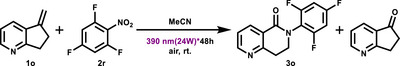		

Entry	Deviation from standard conditions	Yield,(3o,%)^b)^
1	None	66
2	No light	n.d.
3	405nm	63
4	425nm	41
5	EtOAc	62
6	CH_2_Cl_2_	Trace
7	Isobutyronitrile	65
8	MeCN(0.1M)	54
9	MeCN(0.08M)	56
10	Ar atmosphere	66
11	390nm*12W	58
12	390*50W	64

^a)^ Reaction conditions: **1o** (0.3 mmol), **2r** (0.2 mmol), in MeCN (0.04 M), 390 nm irradiation (24 W), under an air atmosphere and room temperature for 48 h unless noted otherwise.

^b)^ Isolated yield. n.d. = not detected.

### 2.2 Substrate Scopes

**Scheme 2:**
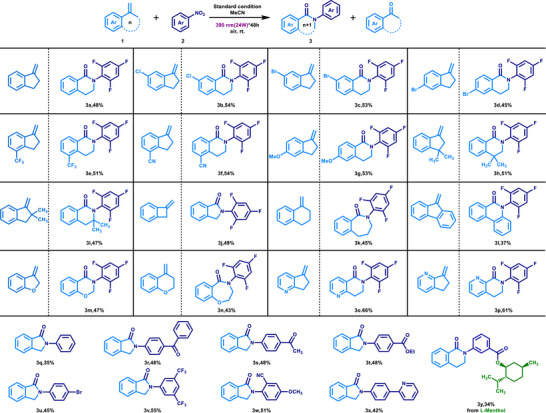
Scope of alkenes and nitroarenes in the synthesis of lactams. Standard condition: **1** (0.3 mmol), **2** (0.2 mmol), in MeCN (0.04 M), 390 nm irradiation (24 W), under an air atmosphere and room temperature for 48 h unless noted otherwise. Isolated yield.

The model reaction was employed to assess the compatibility and scope of various alkenes and nitroarenes. The results demonstrated that diverse cyclic arylalkenes were efficiently converted into the corresponding N‐aryl lactams (**Scheme** **2**). First, the results align with that obtained using 5‐methylene‐6,7‐dihydro‐5H‐cyclopenta[b]pyridine (**1o**) to synthesize 6‐(2,4,6‐trifluorophenyl)‐7,8‐dihydro‐1,6‐naphthyridin‐5(6H)‐one (**3o**). The lactam (**3a**) was obtained with a good yield without any substituents on the aromatic ring. Replacing the aryl groups in arylalkenes with halogens (─Cl, ─Br), electron‐withdrawing groups (─CF3, ─CN), or electron‐donating groups (‐OMe) efficiently produced the corresponding isoquinoline ketones (**3b–3g**), showing that electronic properties and substituents have little effect on the reaction. Next, substituting two methyl groups at the α‐ or β‐position of the arylalkene efficiently yielded the corresponding isoquinoline ketones (**3** **h, 3i**) in good yields. Following confirmation of cyclopentene compatibility, a range of structurally diverse arylalkenes were evaluated to further expand the scope of the synthesized lactam derivatives. Under standard conditions, tetracyclic arylalkene (**1j**) afforded N‐arylindolizine (**3j**) in 49% yield, while hexacyclic arylalkene (**1k**) provided amide (**3k**) in 46% yield. Meanwhile, 9‐methylidenefluorene (**1l**) afforded aryl migration product (**3l**) in 37% yield, demonstrating that aryl groups can migrate in this system, albeit with lower priority than alkyl groups. This outcome contrasts with the classic Beckmann rearrangement rule. Studies on the compatibility of heterocyclic arylalkenes revealed that both five‐ and six‐membered oxygen‐containing arylalkenes were well tolerated (**3m, 3n**). Notably, pyridine‐alkenes outperformed benzene‐alkenes (**3o, 3p**), likely due to favorable electronic properties of the intermediates that promote rearrangement. Overall, the system exhibited consistent reactivity across various arylalkenes, efficiently producing structurally diverse lactams with potential applications in drug scaffold construction. Furthermore, substrate (**1o**) was successfully converted into lactam (**3o,** 54%, 0.75 g) in a gram‐ scale reaction conducted at a 5 mmol scale, which verified the practicality of this strategy.

To evaluate the scope and applicability of nitroarenes in this system, 7‐methylenebicyclo [4.2.0] octa‐1,3,5‐triene (**1j**) was employed as a reactant with various nitroarenes. Neutral nitrobenzene was transformed into N‐phenyl isoindoline (**3q**) in 35% yield. Notably, the reaction efficiency was enhanced when electron‐deficient nitroarenes were employed. For example, nitrobenzenes bearing carbonyl groups afforded the corresponding isoindolines in good yields (**3r–3t**). Halogen (–Br), as well as strongly electron‐withdrawing groups (─CF_3_ ─CN), were also well tolerated, affording the desired products (**3u–3w**) in satisfactory yields. Furthermore, 2‐(4‐nitrophenyl) pyridine underwent the transformation efficiently, yielding compound (**3x**) at 42%. Furthermore, the nitroarene derivative of L‐Menthol was successfully converted into lactam (**3y**) in moderate yield, demonstrating the potential of this strategy for the direct synthesis of lactams with medicinal value. The observed photophysical properties of the electron‐deficient nitroarenes align well with previous reports. For electron‐rich nitroarenes, the high energy barrier associated with inter‐system crossing of excited‐state photons results in markedly distinct optical properties compared to electron‐deficient nitroarenes, thereby limiting compatibility within this system.^[^ ^40^, ^41^, ^42^, ^43^, ^44^, ^45^, ^46^, ^47^, ^48^, ^49^, ^50^, ^51^, ^52^, ^53^, ^54^, ^55^, ^56^, ^57^, ^58^, ^59^, ^60^, ^61^, ^62^, ^63^, ^64^, ^65^, ^66^ ^]^

**Scheme 3:**
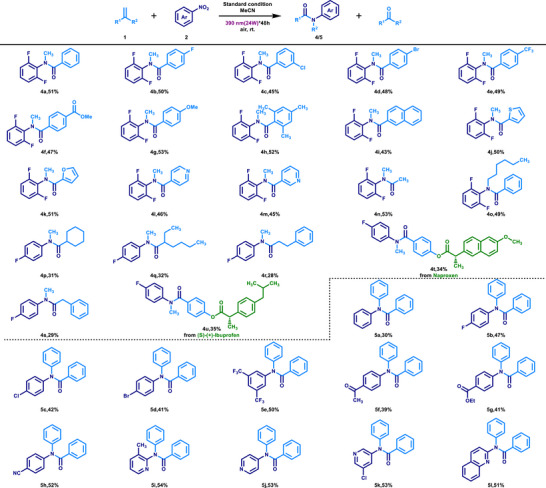
Scope of alkenes and nitroarenes in the synthesis of amides. Standard condition: **1** (0.3 mmol), **2** (0.2 mmol), in MeCN (0.04 M), 390 nm irradiation (24 W), under an air atmosphere and room temperature for 48 h unless noted otherwise. Isolated yield.

The above content has initially demonstrated the ability of cyclic arylalkenes and nitroarenes to construct complex structure lactam derivatives in this reaction. To expand the application scope of this system in the synthesis of amide derivatives, we selected o‐difluoronitrobenzene (**2j**) to react with various α‐alkyl styrenes to evaluate their compatibility (**Scheme** **3**). As expected, the reactivity of various α‐alkyl styrenes was comparable to that of cyclic arylalkenes. Specifically, substrates containing halogen substituents (─F, ─Cl, ─Br) and electron‐withdrawing groups (─CF_3_, ─COOCH_3_, ─CH_3_) could all yield the corresponding N‐methylamides with good yields (**4a–4h**), indicating that the electronic properties of α‐alkyl styrenes had a small impact on the reaction. Moreover, various polycyclic and heterocyclic compounds could also smoothly participate in this reaction and generate the target products (**4i–4m**). Further studies showed that isobutylene could be efficiently converted into the corresponding amide (**4n**), and n‐butyl could also undergo rearrangement to generate the amide (**4o**), thereby enhancing the structural diversity of the obtained amide products and demonstrating its wide application potential in the synthesis of amide skeleton‐based drugs. To further evaluate the migration selectivity, we investigated a series of unsymmetrical dialkyl alkenes in this reaction system. When 4‐fluoronitrobenzene was employed as the nitro source, the transformation proceeded smoothly, affording the desired products in moderate yields (**4p–s**). Notably, methyl groups demonstrated a distinct preference for migration over primary or secondary alkyl substituents. Meanwhile, α‐methylstyrenes functionalized with naproxen or (S)‐(+)‐ibuprofen undergo smooth conversion into the corresponding amide products in moderate yields (**4t–4u**), demonstrating the potential of this strategy for the direct construction of amides bearing pharmaceutically relevant motifs.

Benzene has been proven to have a similar migration ability to alkyl groups, although its migration activity is lower than that of alkyl groups. Based on this, we evaluated the ability of different nitroarenes and 1,1‐diphenylethylene (**1q**) to form N,N‐diphenylamides in this system. The results showed that nitroarenes with electron‐deficient characteristics exhibited good reactivity in this system. Compared with the reaction performance of nitrobenzene, nitroarenes containing halogens (─F, ─Cl, ─Br), electron‐deficient groups (─CF_3_, ─COCH_3_, ─COOCH_3_, ─CN), and electron‐deficient heterocyclic or polycyclic structures could all efficiently generate the corresponding N,N‐diphenylamides with good yields (**5a–5l**). Furthermore, in the evaluation of unsymmetrical biaryl alkenes, 4‐(1‐phenylvinyl)pyridine (**1s**) was also smoothly converted into amide product (**5j**) in 46% yield (see SI), demonstrating that the heteroaryl group exhibits higher migratory aptitude than the phenyl group.

In addition to terminal alkenes, the compatibility of multi‐substituted alkenes was systematically investigated (see SI, Table S2). For trisubstituted alkenes (**1aa–1ad**), the electronic nature of the substituents (─COOEt, ─CH_3_, ─OCH_3_, ─F) exerted minimal influence on the reaction efficiency. However, the overall yields were lower than those observed for terminal alkenes, likely due to increased steric hindrance. Tetrasubstituted alkenes displayed a similar reactivity trend (**1ae**), with a further reduction in yield attributed to greater steric congestion around the double bond.

### 2.3 DFT Calculations and Proposed Mechanism

**Scheme 4:**
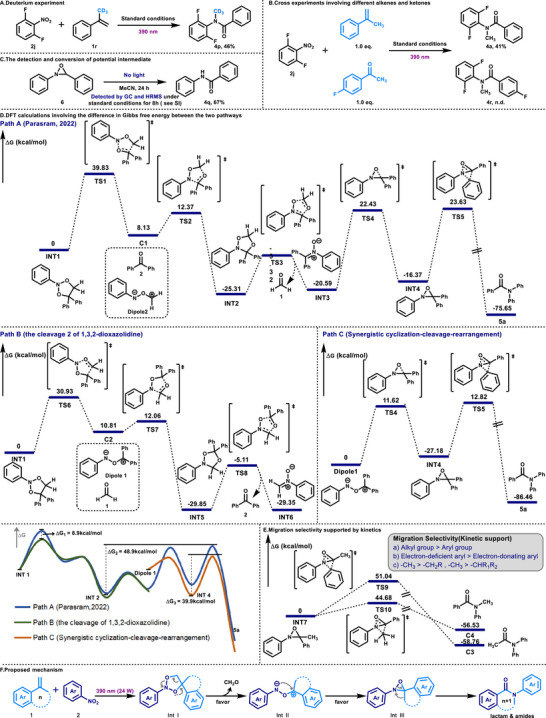
A) Deuterium labeling experiment involving alkyl migration; B) Cross experiments involving different alkenes and ketones have provided new insights into the mechanism; C) The detection and conversion of potential intermediate; D) DFT calculations support the difference in Gibbs free energy between the two pathways; E) Migration selectivity supported by kinetics; F) Proposed mechanism.

To explore the detailed mechanism of this reaction, we conducted a series of mechanism studies and DFT calculations (**Scheme** **4**). First, after adding radical inhibitors (TEMPO and BHT), the reactions were significantly inhibited (see SI). This phenomenon is related to the radical characteristics of the excited‐state nitroarenes. Second, when using α‐CD_3_ styrene for the reaction, a deuterated product (**4p**) was obtained (Scheme 4A). The labeling of the deuterium element confirmed the migration ability of the alkyl group, and it was confirmed that this strategy achieved the sp^3^ C─C bond insertion of the nitrogen atom. Moreover, compared with the pathway proposed by Parasram previously ‐ that is,^[^ ^48^, ^49^, ^50^ ^]^ through the ring‐opening of 1,3,2‐dioxazolidine to generate carbonyl compounds and carbonyl imine intermediates, then undergoing dipole cycloaddition, and through 1,4,2‐dioxazolidine ring‐opening to generate nitrone, and finally rearranging to form amide through light induction (Scheme 4D Path A) ‐ we carried out cross‐control experiments on the reaction of o‐difluoronitrobenzene (**2j**) with α‐methyl styrene and p‐fluorobenzoylacetone. The results showed that the reaction could obtain amide product (**4a**) with good yield, but no compound (**4r**) was detected, which to some extent questioned the possible amide formation pathway proposed previously (Scheme 4B). Then the potential intermediate (**6**) was detedted by GC and HRMS under standard conditions for 8 h, and it was synthesized to evaluate its transformation within this system. Under a light‐free atmosphere, amide (**4q**) was obtained with a yield of 67% after 24 h (Scheme 4C).

Furthermore, the DFT theoretical calculations conducted a detailed study on two possible reaction pathways: one is the nitrone photo‐induced rearrangement pathway,^[^ ^42^, ^43^ ^]^ and the other is the intramolecular concerted cyclization‐cleavage‐rearrangement pathway directly involving the N─O═C dipole (Scheme 4D). The calculation results indicate that there are two significant differences in the Gibbs free energy changes of the two pathways, providing a theoretical basis for determining the mechanism. First, when 1,3,2‐dioxazolidine (**INT1**) undergoes ring‐opening to generate two different carbonyl imide intermediates, due to differences in charge distribution and stability of the N─O═C dipole, the Gibbs free energy difference between transition states is relatively significant (ΔG_1_ ≈ 8.9 kcal mol^−1^). Second, compared to the energy barrier required for generating amides through nitrone photo‐induced rearrangement (ΔG_2_ ≈ 48.9 kcal mol^−1^), the energy barrier required for generating amides through N─O═C dipole undergoing intramolecular concerted cyclization‐cleavage‐rearrangement process is lower (ΔG_3_ ≈ 39.9 kcal mol^−1^).

Additionally, the migration selectivity of different groups and the deeper mechanism were investigated (Scheme 4E). In the study of the reactivity of alkenes, it was found that alkyl migration always outperformed aryl migration. This phenomenon is contrary to the classical Beckmann rearrangement, in which aryl migration can more effectively stabilize the partially positive charge formed in the transition state through the conjugation effect. DFT calculations indicate that when the intermediate (**INT7**) undergoes alkyl migration, its transition state energy barrier is lower than that of aryl migration, and the free energy barrier difference between the two is significant (ΔG^‡^ ≈ 6.4 kcal mol^−1^). This result supports the reaction pathway of alkyl migration being preferred over aryl migration from the kinetic perspective. Similar mechanisms can be applied to unsymmetrical dialkyl alkenes and unsymmetrical diaryl alkenes: in the former, methyl migration is superior to primary or secondary alkyl; in the latter, the migration of electron‐deficient aryl groups is preferred over phenyl.

Based on the Gibbs free energy differences derived from DFT calculations and supported by the cross‐control experimental results and the conversion of potential intermediate, we propose a plausible mechanism for the reaction (Scheme 4F). Under irradiation at 390 nm (24 W), nitroarene (**2**) and cyclic arylalkenes (**1**) undergo a stepwise [3+2] radical cycloaddition to form the intermediate 1,3,2‐dioxazolidine (**IntI**). Owing to the differences in Gibbs free energy among the corresponding transition states, **IntI** preferentially transforms into formaldehyde and the carbonyl imide intermediate (**IntII**). Subsequently, **IntII** undergoes an intramolecular concerted cyclization–cleavage–rearrangement process to obtain **IntIII**, ultimately leading to the efficient formation of the lactam product. It is essential to acknowledge that, compared with the pathways leading to the formation of carbonyl compounds in this system,^[^ ^48^, ^49^, ^50^ ^]^ the dominance of the proposed pathway in terms of kinetics primarily arises from structural differences among the alkenes involved. The behavior of the N─O═C dipoles generated from different alkenes in subsequent reactions exhibits notable variations. Furthermore, the photon utilization efficiency provided by light sources of varying power also exerts a certain influence on the selectivity of the reaction pathway.

## 3 Conclusion

This study reports the first example of skeletal editing of alkenes via the cascade cleavage of C(sp^2^)═C(sp^2^) and C(sp^3^)─C(sp^3^) bonds, enabling the insertion of the nitrogen atom and thereby the synthesis of lactam and amide derivatives, using nitroarenes as nitrogen atom insertion reagents under light irradiation. This approach highlights the broad potential of integrating skeletal editing with N─O═C dipole chemistry in organic synthesis. The reaction features environmental friendliness, high efficiency, and operational simplicity, enabling the preparation of structurally diverse lactam and amide derivatives through the use of various alkene substrates. Furthermore, both experiments and DFT calculations support the involvement of N─O═C dipoles in an intramolecular concerted cyclization–cleavage–rearrangement process, thereby expanding the scope of dipolar chemistry. Looking ahead, the reactivity of N─O═C dipoles — generated from the photoinduced oxidative cleavage of alkenes with nitroarenes — has evolved from classical dipolar cycloadditions to 3,3‐sigma rearrangements of O‐alkenyl hydroxylamines with specific alkenes, and further to unique intramolecular rearrangement processes. As a versatile dipolar intermediate, the N─O═C dipole holds significant promise for developing novel synthetic methodologies that serve skeletal editing and contribute to pharmaceutical synthesis.

## Conflict of Interest

The authors declare no conflict of interest.

## Supporting information



Supporting Information

## Data Availability

The data that support the findings of this study are available in the Supporting Information of this article.
