# Supplementary material for: Skeletal Editing of Alkenes with Nitroarenes via Photoinduced Rearrangement of N─O═C Dipoles Forms Lactams and Amides
Source: Adv Sci (Weinh). 2025 Nov 23;13(6):e18828. doi: 10.1002/advs.202518828 (PMC12866758; doi:10.1002/advs.202518828)

**Supporting Information**

**Skeletal Editing of Alkenes with Nitroarenes via Photoinduced Rearrangement of N-O=C Dipoles Forms Lactams and Amides**

Hongyun Qin,^[a]^ Zemin Wang,^[a]^ Cong Shi,^[a]^ Jiashu Chen,^[a]^ Chao Liu,^[a]^ Xiangqian Li,^[a]^ Ruihua Liu,* ^[a]^ and Dayong Shi*^[a][b][c]^

[a] State Key Laboratory of Microbial Technology, Shandong University, Qingdao, 266237, Shandong, P. R. China
E-mail: shidayong@sdu.edu.cn

[b] Shenzhen Research Institute of Shandong University, Shenzhen, 518057, Guangdong, P. R. China.

[c] Laboratory for Marine Drugs and Bioproducts, Qingdao Marine Science and Technology Center, Qingdao, 266237, Shandong, P. R. China

**Contents**

[1. General information 2](#_Toc212214940)

[2. Optimization of the reaction conditions 3](#_Toc212214941)

[3. General procedure 5](#_Toc212214942)

[4. Substrate synthesis 6](#_Toc212214943)

[5. Characterization of products 8](#_Toc212214944)

[6. Mechanistic studies 27](#_Toc212214945)

[**6.1 Redical quenching experiments 27**](#_Toc212214946)

[**6.2 Deuterium labeling experiment 27**](#_Toc212214947)

[**6.3 The capture and conversion of potential intermediate 28**](#_Toc212214948)

[**6.4 Cross-over study 30**](#_Toc212214949)

[**6.5 DFT calculations of energy barriers for two pathways involving the N-O=C dipole 30**](#_Toc212214950)

[**6.6 Migration selectivity supported by DFT calculations 31**](#_Toc212214951)

[7. Copies of product NMR spectra 61](#_Toc212214952)

# General information

All reaction involving air- and moisture-sensitive compounds were carried out in the argon-filled glove box or by standard Schlenk techniques under argon atmosphere. Unless otherwise noted, chemicals and solvents were purchased from commercial suppliers (Alfa, Adamas-beta® Aldrich, Innochem, Stream, and so on) and used without further purification. All new compounds were fully characterized. Reactions were monitored by thin layer chromatography (TLC) using glass 0.25 mm silica gel plates. Column chromatography was performed on 200-300 mesh silica gel.

All NMR spectra (^1^H, ^13^C, and ^19^F) were recorded on Bruker AVANCE NEO (600 MHz) and Bruker AVANCE III (400 MHz) spectrometers. All chemical shifts (δ) were given in ppm and coupling constants (J) were provided in Hz. Multiplicities are abbreviated as follows: singlet (s), doublet (d), triplet (t), quartet (q), and multiplet (m). Gas chromatographic (GC) analyses were acquired on a SHIMADZU GC 2030 gas chromatography instrument with a FID detector and adamantane was added as an internal standard. Melting points were determined with melting point apparatus SGW X-4A and were not corrected. High resolution mass spectra (HRMS) analysis was performed on a Thermo Fisher Q-Exactive instrument. The reactions were performed in a photocatalytic reaction unit HJY-1201.

| **Abbreviation notes** | | | |
| --- | --- | --- | --- |
| **Abbreviation** | **The name** | **Abbreviation** | **The name** |
| BHT | butylated hydroxytoluene | EA | ethyl acetate |
| DCM | dichloromethane | PE | petroleum ether (boiling  range: 60-90 ℃) |
| DMF | N,N-Dimethylformamide | THF | tetrahydrofuran |
| DMSO | dimethyl sulfoxide | TEMPO | 2,2,6,6-tetramethyl-1-  piperadoxyl |

# Optimization of the reaction conditions

**Table S1.** Optimization of solvent, equivalency, concentration, wavelength, power, temperature and time course.^a^

^a^Reactions were conducted on a 0.20 mmol scale. ^b^Isolated after silica gel chromatography. n.d. = not detected.

**Table S2.** Scope of various various multi-substituted alkenes^a,b^

^a^Reaction conditions: **1** (0.3 mmol), **2r** (0.2 mmol), in MeCN (0.04 M), 390 nm irradiation (24 W), under air atmosphere and room temperature for 48 h unless noted otherwise. ^b^Isolated after silica gel chromatography.

# General procedure

**General Procedure A:** Standard conditions

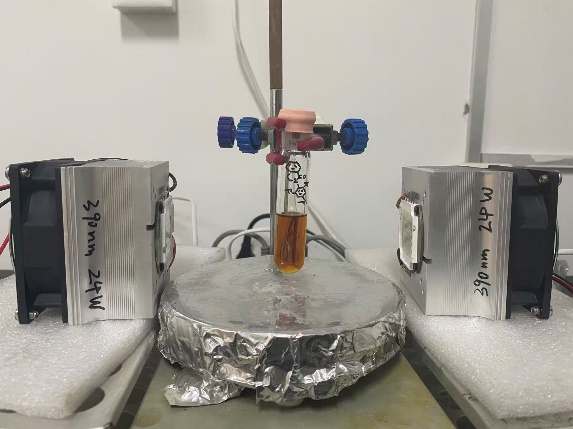

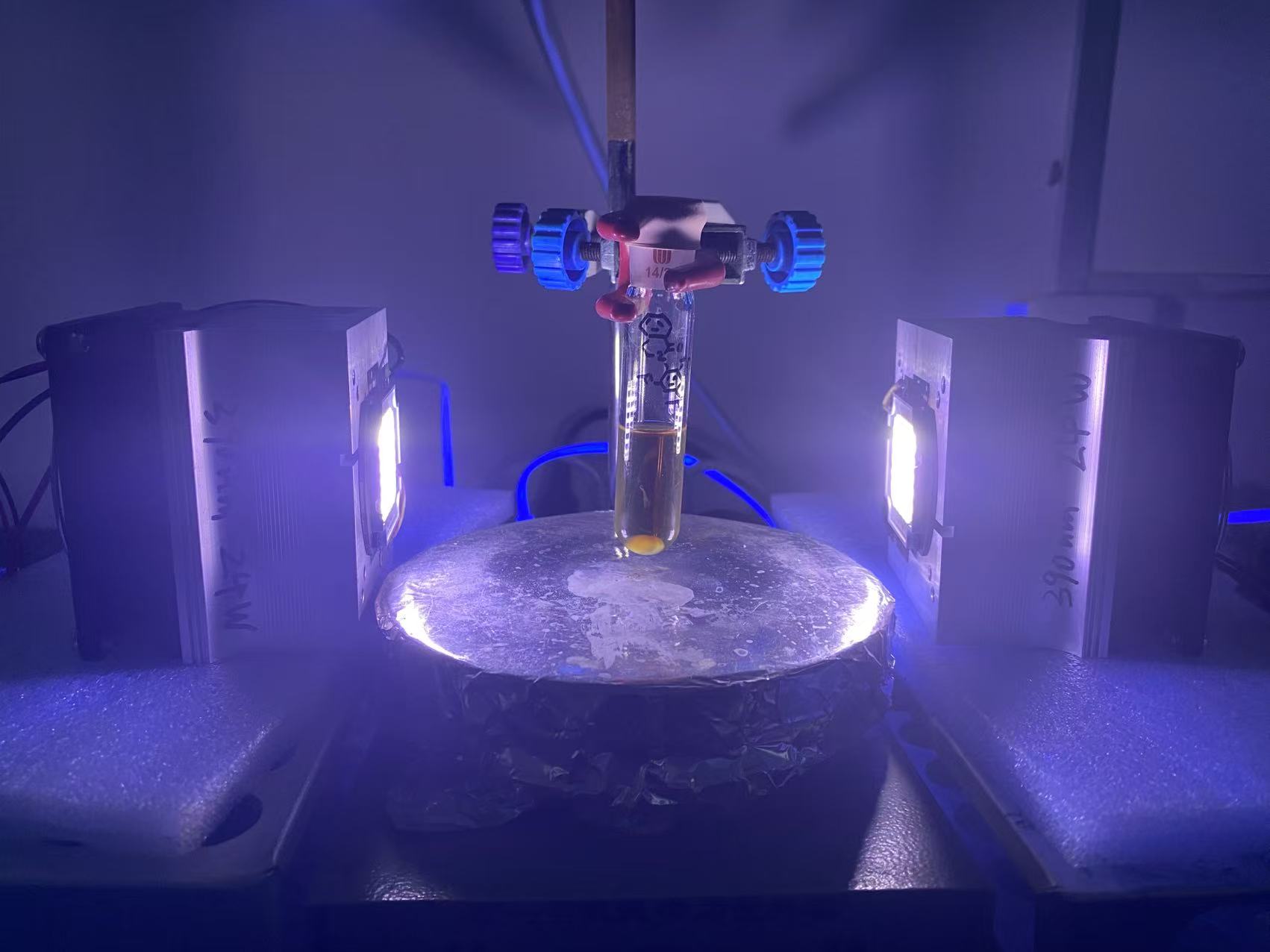


Alkene **1** (0.3 mmol, 1.5 equiv.), nitro(hetero)arenes **2** (0.2 mmol, 1.0 equiv.), were added to a 15 mL scintillation vial (flame dried or oven dried). Then MeCN (5.0 mL, 0.04 M) was added to the reaction. The reaction mixture was stirred and illuminated under blue LEDs (390 nm, 24W) and air for 48 hours until full conversion of nitro(hetero)arenes as determined by TLC or GCMS analysis. Upon completion of the reaction, the reaction mixture was concentrated to remove the solvent, and then the pure product was obtained by flash column chromatography on silica gel.

**General Procedure B:** Scale up procedure

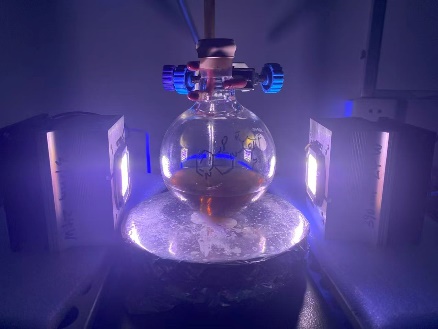

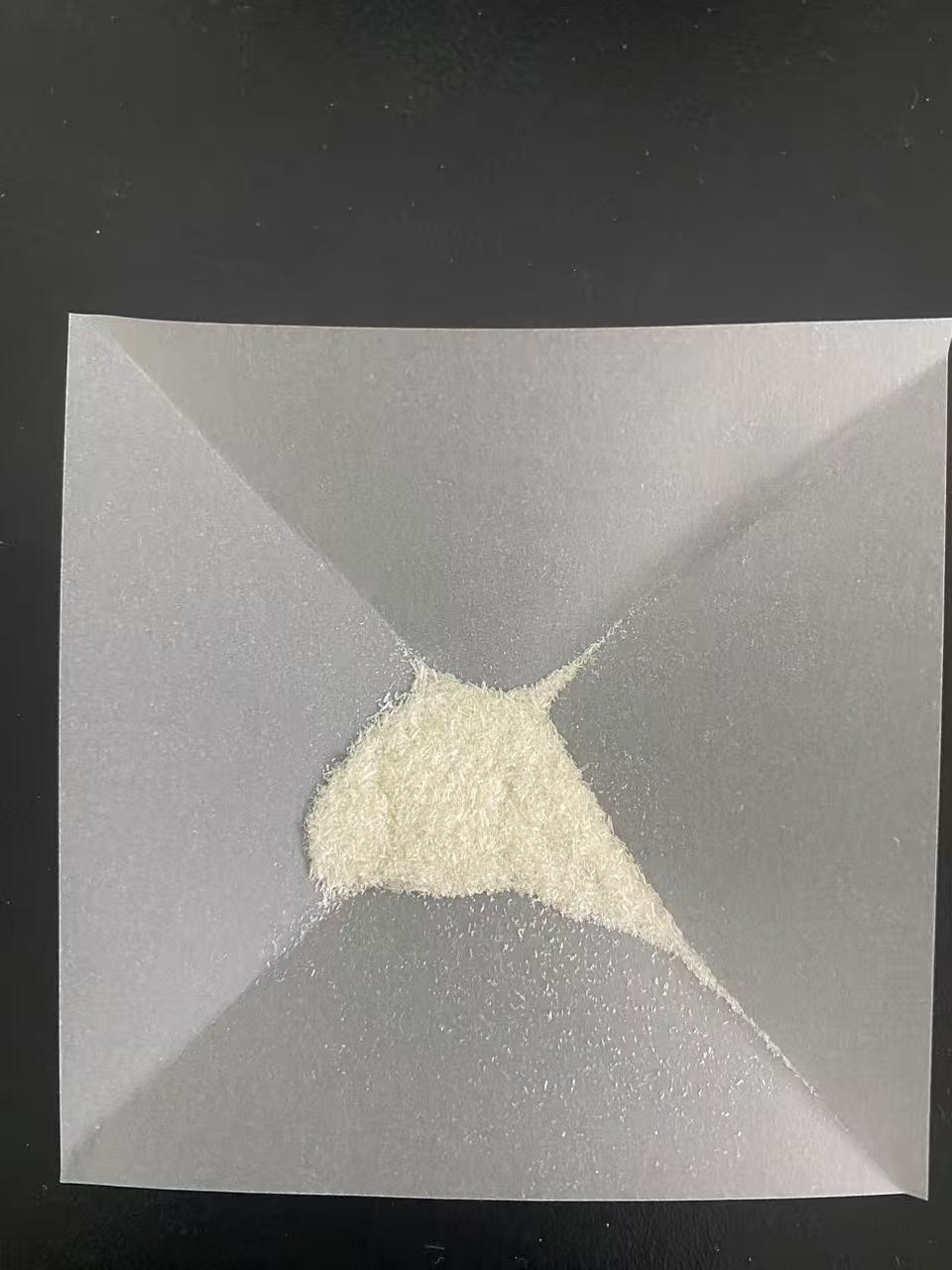


5-methylene-6,7-dihydro-5H-cyclopenta[b]pyridine **1o** (0.983 g, 7.5 mmol, 1.5 equiv.), 1,3,5-Trifluoro-2-nitrobenzene **2r** (0.885 g, 5 mmol, 1.0 equiv.), were added to a 250 mL round-bottomed flask (flame dried or oven dried). Then MeCN (125 mL, 0.04 M) was added to the reaction. The reaction mixture was stirred and illuminated under blue LEDs (390 nm, 24W) and air for 60 hours until full conversion of nitro(hetero)arenes as determined by TLC or GCMS analysis. Upon completion of the reaction, the reaction mixture was concentrated to remove the solvent, and then the pure product **3o** (0.75 g, 54%) was obtained by flash column chromatography on silica gel.

# Substrate synthesis


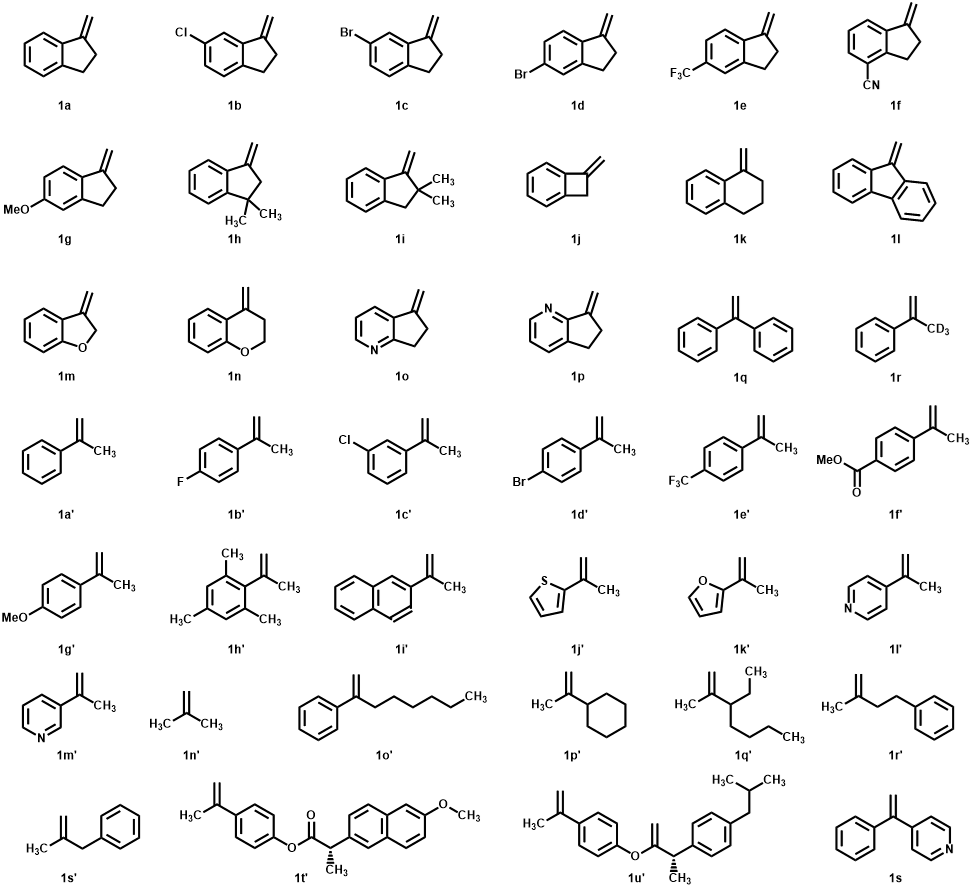
**Table S3.** Used alkenes


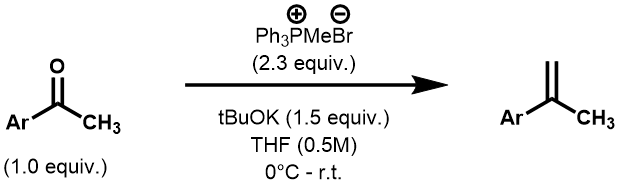
Unless otherwise noted, the olefins were synthesized according to reported procedures.

In an oven dried round bottom flask, Methyltriphenylphosphonium bromide (2.3 equiv.) was dissolved in anhydrous THF (0.5 M) at 0℃, followed by the addition of Potassium tert-butoxide (1.5 equiv). Mixture was stirred for 30 min-1hr. Then, the chosen carbonyl (1.0 equiv) was dissolved in anhydrous THF and added dropwise at 0℃. The reaction was left to warm gradually and stirred until reaction had gone to completion, as monitored by TLC. After completion the reaction was diluted with Et_2_O and washed with saturated aqueous sodium chloride (sat. aq. NaCl) solution. Product was extracted three times into CH_2_Cl_2_ or EtOAc. The organic layers were dried (Na_2_SO_4_), concentrated, and purified by column chromatography to give the desired alkene product.


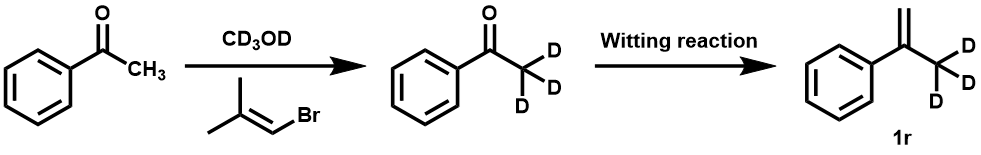
Acetophenone (1.0 equiv.) and prenyl bromide (1.2 equiv.) were mixed in methanol-d4 and stirred at room temperature for 5 h, then the reaction solution was evaporated in vacuo. The residue was purified by flash column chromatography (silica gel, EA/PE = 1:10 as an eluent) to afford the desired product Acetophenone-d_3_. Then, the substrate **1r** was obtained through witting reaction. All the products were also confirmed by comparing the ^1^H NMR and ^13^C NMR data with authentic samples.


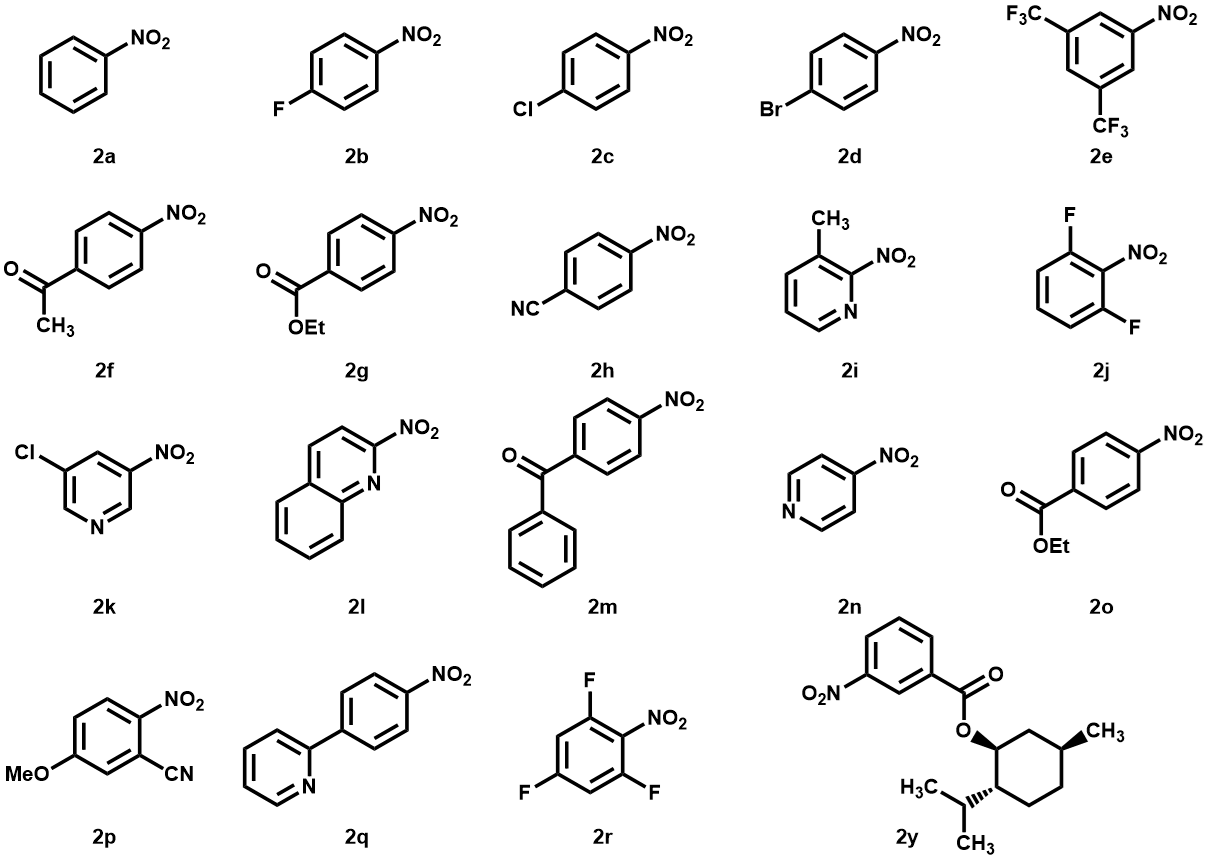
**Table S4.** Used nitro(hetero)arenes


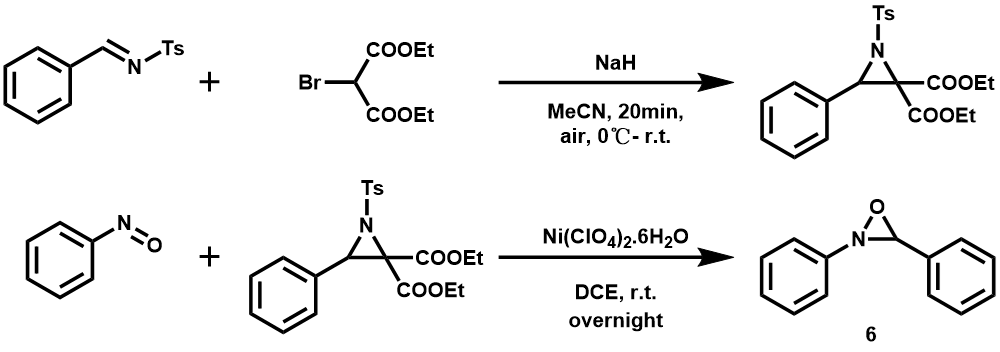
Unless otherwise noted, the nitro(hetero)arenes described above were purchased from commercial suppliers (Alfa, Adamas-beta® Aldrich, Innochem, Stream, and so on) and used without further purification, unreported ones were identified by their NMR spectroscopic character.

In an inert atmosphere, a solution of imine (0.5 mmol, 1 equiv), 2-bromomalonate (0.55 mmol, 1.1 equiv) in 5 mL of dry CH_3_CN was cooled to 0℃, and treated with NaH (22 mg, 60% dispersion in mineral oil, 1.1 equiv). The resultant mixture was warmed up and stirred at room temperature. After 20 mins when the reaction completed (determined by TLC analysis), the reaction mixture was then passed over a small plug of silica gel eluted with CH_2_Cl_2_ to remove the excess NaH and resulting NaBr. After evaporation under reduced pressure, usually, the curde product was in enough purity, which can be quickly purified by flash chromatography on 10cm-long silica gel (eluent, 10-30% ethyl acetate in petroleum ether) to afford the desired product diethyl 3-phenyl-1-tosylaziridine-2,2-dicarboxylate.

Then, diethyl 3-phenyl-1-tosylaziridine-2,2-dicarboxylate (0.34 mmol), nitrosobenzene (0.3 mmol), Ni(C10_4_)_2_·6H_2_O (0.009 mmol), and 1,2-dichloroethane (1.5 mL) were added to a test tube, and the mixture was stirred at room temperature overnight. The crude product was obtained through extraction, washing, and drying. The crude material was then purified by silica gel column chromatography (PE/EA = 20:1) to yield cyclization product 2,3-diphenyl-1,2-oxaziridine (**6**).

# Characterization of products


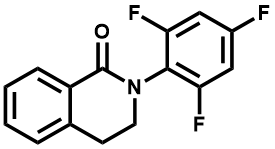
**2-(2,4,6-trifluorophenyl)-3,4-dihydroisoquinolin-1(2H)-one (3a)**

According to the general procedure A, the product **3a** was obtained after silica gel chromatography (PE/EA: 10/1).

White solid; 26.6 mg, 48% yield; M.p. = 160-162ºC.

**^1^H NMR** (400 MHz, CDCl_3_) δ 8.14 (d, *J* = 7.7 Hz, 1H), 7.50 (t, *J* = 7.4 Hz, 1H), 7.39 (t, *J* = 7.5 Hz, 1H), 7.26 (d, *J* = 3.6 Hz, 1H), 6.79 (t, *J* = 8.1 Hz, 2H), 3.87 (t, *J* = 6.4 Hz, 2H), 3.19 (t, *J* = 6.4 Hz, 2H).

**^13^C NMR** (151 MHz, CDCl_3_) δ 163.9, 161.4 (dt, *J* = 249.8, 14.7 Hz), 159.1 (ddd, *J* = 252.6, 15.3, 7.6 Hz), 138.7, 132.5, 128.9, 128.7, 127.3, 127.2, 115.2, 100.9 (dd, *J* = 28.2, 26.3 Hz), 49.11, 28.6.

**^19^F NMR** (376 MHz, CDCl_3_) δ -107.99, -114.04.

**FTMS (ESI)** m/z: [M+H]^+^ calcd for C_15_H_11_F_3_NO^+^ 278.0787; found 278.0789.


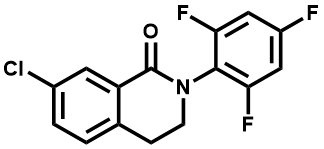
**7-chloro-2-(2,4,6-trifluorophenyl)-3,4-dihydroisoquinolin-1(2H)-one (3b)**

According to the general procedure A, the product **3b** was obtained after silica gel chromatography (PE/EA: 10/1).

White solid; 33.6 mg, 54% yield; M.p. = 138-139ºC.

**^1^H NMR** (400 MHz, CDCl_3_) δ 8.09 (d, *J* = 2.3 Hz, 1H), 7.45 (dd, *J* = 8.1, 2.3 Hz, 1H), 7.21 (d, *J* = 8.1 Hz, 1H), 6.79 (td, *J* = 9.7, 2.1 Hz, 2H), 3.85 (t, *J* = 6.5 Hz, 2H), 3.15 (t, *J* = 6.6 Hz, 2H).

**^13^C NMR** (101 MHz, CDCl_3_) δ 162.7, 162.0 (d, *J* = 240.1 Hz), 159.0 (ddd, *J* = 22.7, 15.2, 7.0 Hz), 136.9, 134.6, 133.4, 132.5, 130.1, 128.7, 115.9, 101.1 (dd, *J* = 28.0, 26.0 Hz), 49.0, 28.0.

**^19^F NMR** (376 MHz, CDCl_3_) δ -107.5, -114.1.

**FTMS (ESI)** m/z: [M+H]^+^ calcd for C_15_H_10_ClF_3_NO^+^ 312.0398; found 312.0401.


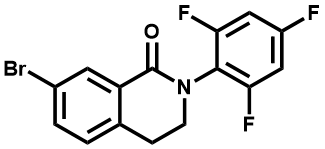
**7-bromo-2-(2,4,6-trifluorophenyl)-3,4-dihydroisoquinolin-1(2H)-one (3c)**

According to the general procedure A, the product **3c** was obtained after silica gel chromatography (PE/EA: 10/1).

Pale yellow solid; 37.6 mg, 53% yield; M.p. = 172-173ºC.

**^1^H NMR** (400 MHz, CDCl_3_) δ 8.26 (d, *J* = 2.1 Hz, 1H), 7.61 (dd, *J* = 8.1, 2.2 Hz, 1H), 7.15 (d, *J* = 8.1 Hz, 1H), 6.79 (td, *J* = 9.7, 2.1 Hz, 2H), 3.85 (t, *J* = 6.5 Hz, 2H), 3.13 (t, *J* = 6.5 Hz, 2H).

**^13^C NMR** (101 MHz, CDCl_3_) δ 162.6, 161.9 (d, *J* = 243.6 Hz), 158.9 (ddd, *J* = 22.6, 13.9, 7.5 Hz), 137.4, 135.4, 131.7, 130.4, 129.0, 121.1, 115.9 (d, *J* = 5.1 Hz), 101.0 (dd, *J* = 28.0, 26.0 Hz), 49.0, 28.1.

**^19^F NMR** (376 MHz, CDCl_3_) δ -107.4, -114.1.

**FTMS (ESI)** m/z: [M+H]^+^ calcd for C_15_H_10_BrF_3_NO^+^ 355.9892; found 355.9896.


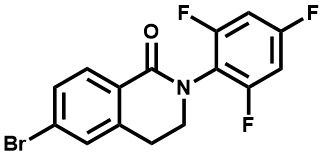
**6-bromo-2-(2,4,6-trifluorophenyl)-3,4-dihydroisoquinolin-1(2H)-one (3d)**

According to the general procedure A, the product **3d** was obtained after silica gel chromatography (PE/EA: 10/1).

Pale yellow solid; 31.9 mg, 45% yield; M.p. = 180-181ºC.

**^1^H NMR** (600 MHz, CDCl_3_) δ 7.99 (d, *J* = 8.3 Hz, 1H), 7.53 (dd, *J* = 8.3, 1.8 Hz, 1H), 7.06 (d, *J* = 8.5 Hz, 3H), 6.79 (dd, *J* = 12.9, 5.2 Hz, 2H), 6.76 – 6.74 (m, 2H), 3.86 (t, *J* = 6.5 Hz, 2H), 3.16 (t, *J* = 6.5 Hz, 2H).

**^13^C NMR** (151 MHz, CDCl_3_) δ 163.2, 161.6 (d, *J* = 250.9 Hz), 159.0 (d, *J* = 252.7 Hz), 140.4, 132.8, 130.7, 130.6, 130.2, 129.4, 115.3, 101.0 (t, *J* = 27.3 Hz), 48.9, 28.3.

**^19^F NMR** (565 MHz, CDCl_3_) δ -107.5, -114.1.

**FTMS (ESI)** m/z: [M+H]^+^ calcd for C_15_H_10_BrF_3_NO^+^ 355.9892; found 355.9892.


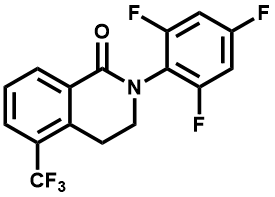
**5-(trifluoromethyl)-2-(2,4,6-trifluorophenyl)-3,4-dihydroisoquinolin-1(2H)-one (3e)**

According to the general procedure A, the product **3e** was obtained after silica gel chromatography (PE/EA: 10/1).

White solid; 35.2 mg, 51% yield; M.p. = 186-188ºC.

**^1^H NMR** (400 MHz, CDCl_3_) δ 8.37 (d, *J* = 7.8 Hz, 1H), 7.85 (d, *J* = 7.8 Hz, 1H), 7.51 (t, *J* = 7.8 Hz, 1H), 6.81 (td, *J* = 9.7, 2.0 Hz, 2H), 3.89 (t, *J* = 6.5 Hz, 2H), 3.37 (t, *J* = 6.3 Hz, 2H).

**^13^C NMR** (151 MHz, CDCl_3_) δ 162.6, 161.7 (dt, *J* = 249, 14.5 Hz), 159.0 (ddd, *J* = 252.9, 15.3, 7.5 Hz), 137.3, 132.6, 130.5, 129.6 (q, *J* = 5.2 Hz), 127.9, 127.7, 127.2, 115.6 (td, *J* = 17.0, 5.1 Hz), 101.1 (td, *J* = 26.7, 5.0 Hz), 48.2, 25.5 (d, *J* = 1.8 Hz).

**^19^F NMR** (565 MHz, CDCl_3_) δ -60.5, -107.3, -114.0.

**FTMS (ESI)** m/z: [M+H]^+^ calcd for C_16_H_10_F_6_NO^+^ 346.0661; found 346.0661.


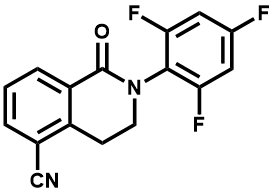
**1-oxo-2-(2,4,6-trifluorophenyl)-1,2,3,4-tetrahydroisoquinoline-5-carbonitrile (3f)**

According to the general procedure A, the product **3f** was obtained after silica gel chromatography (PE/EA: 5/1).

White solid; 32.6 mg, 54% yield; M.p. = 188-190ºC.

**^1^H NMR** (400 MHz, CDCl_3_) δ 8.37 (dd, *J* = 7.9, 1.2 Hz, 1H), 7.83 (dd, *J* = 7.8, 1.3 Hz, 1H), 7.52 (t, *J* = 7.8 Hz, 1H), 6.81 (td, *J* = 9.7, 2.1 Hz, 2H), 3.94 (t, *J* = 6.5 Hz, 2H), 3.42 (t, *J* = 6.5 Hz, 2H).

**^13^C NMR** (101 MHz, CDCl_3_) δ 162.0, 161.8 (dt, *J* = 252.9 ,15.0 Hz), 158.9 (ddd, *J* = 252.9, 15.2, 7.4 Hz), 142.2, 136.1, 133.2, 129.9, 128.3, 127.9, 116.7, 111.6, 101.1 (td, *J* = 26.7, 5.0 Hz), 48.3, 27.0.

**^19^F NMR** (376 MHz, CDCl_3_) δ -106.9, -114.1.

**FTMS (ESI)** m/z: [M+H]^+^ calcd for C_16_H_10_F_3_N_2_O^+^ 303.0740; found 303.0743.


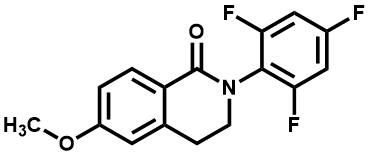
**6-methoxy-2-(2,4,6-trifluorophenyl)-3,4-dihydroisoquinolin-1(2H)-one (3g)**

According to the general procedure A, the product **3g** was obtained after silica gel chromatography (PE/EA: 10/1).

Pale yellow solid; 32.5 mg, 53% yield; M.p. = 138-140ºC.

**^1^H NMR** (600 MHz, CDCl_3_) δ 7.69 (d, *J* = 9.1 Hz, 1H), 7.00 – 6.85 (m, 3H), 6.78 (t, *J* = 8.0 Hz, 1H), 3.89 (s, 3H), 3.09 (t, *J* = 6.5 Hz, 2H), 2.68 (t, *J* = 6.5 Hz, 2H).

**^13^C NMR** (151 MHz, CDCl_3_) δ 165.3, 163.4 (d, *J* = 140.6 Hz), 160.3 (d, *J* = 63.9 Hz), 158.2, 140.8, 131.1, 130.5, 125.4, 115.3, 109.8, 100.9 (t, *J* = 27.0 Hz), 55.6, 49.1, 25.9.

**^19^F NMR** (565 MHz, CDCl_3_) δ -108.3, -114.1.

**FTMS (ESI)** m/z: [M+H]^+^ calcd for C_16_H_13_F_3_NO_2_^+^ 308.0893; found 308.0897.


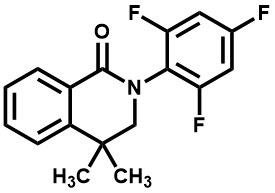
**4,4-dimethyl-2-(2,4,6-trifluorophenyl)-3,4-dihydroisoquinolin-1(2H)-one (3h)**

According to the general procedure A, the product **3h** was obtained after silica gel chromatography (PE/EA: 10/1).

White solid; 31.1 mg, 51% yield; M.p. = 164-165ºC.

**^1^H NMR** (400 MHz, CDCl_3_) δ 8.16 (dd, *J* = 8.0, 1.2 Hz, 1H), 7.55 (t, *J* = 6.9 Hz, 1H), 7.38 (t, *J* = 7.0 Hz, 2H), 6.79 (t, *J* = 8.1 Hz, 2H), 3.64 (s, 2H), 1.45 (s, 6H).

**^13^C NMR** (151 MHz, CDCl_3_) δ 163.8, 161.4 (dt, *J* = 250.0, 14.9 Hz), 159.2 (ddd, *J* = 252.8, 15.2, 7.6 Hz), 147.3, 133.0, 129.2, 127.1, 126.9, 123.6, 116.2 (td, *J* = 16.6, 5.1 Hz), 100.9 (td, *J* = 26.7, 5.2 Hz), 60.8 (s), 34.9, 26.6.

**^19^F NMR** (565 MHz, CDCl_3_) δ -108.0, -113.8.

**FTMS (ESI)** m/z: [M+H]^+^ calcd for C_17_H_15_F_3_NO^+^ 306.1100; found 306.1101.


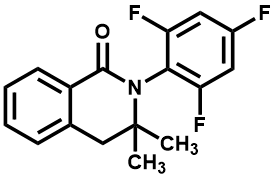
**3,3-dimethyl-2-(2,4,6-trifluorophenyl)-3,4-dihydroisoquinolin-1(2H)-one (3i)**

According to the general procedure A, the product **3i** was obtained after silica gel chromatography (PE/EA: 10/1).

White solid; 28.7 mg, 47% yield; M.p. = 167-168ºC.

**^1^H NMR** (600 MHz, CDCl_3_) δ 7.21 (d, *J* = 7.3 Hz, 1H), 7.11 (t, *J* = 7.2 Hz, 1H), 7.04 (t, *J* = 7.4 Hz, 1H), 6.84 (td, *J* = 9.1, 1.8 Hz, 2H), 6.35 (d, *J* = 8.0 Hz, 1H), 2.94 (s, 2H), 1.28 (s, 6H).

**^13^C NMR** (151 MHz, CDCl_3_) δ 175.0, 162.3 (d, *J* = 251.3 Hz), 159.7 (d, *J* = 238.9 Hz), 139.1, 128.7, 127.4, 124.4, 123.5, 114.4, 112.1, 101.3 (t, *J* = 27.3 Hz), 40.0, 29.7, 24.4.

**^19^F NMR** (565 MHz, CDCl_3_) δ -106.2, -114.8.

**FTMS (ESI)** m/z: [M+H]^+^ calcd for C_17_H_15_F_3_NO^+^ 306.1100; found 306.1100.


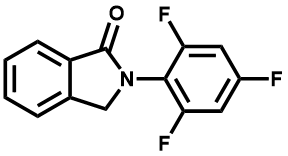
**2-(2,4,6-trifluorophenyl)isoindolin-1-one (3j)**

According to the general procedure A, the product **3j** was obtained after silica gel chromatography (PE/EA: 10/1).

White solid; 25.8 mg, 49% yield; M.p. = 165-166ºC.

**^1^H NMR** (600 MHz, CDCl_3_) δ 7.97 (d, *J* = 7.6 Hz, 1H), 7.63 (t, *J* = 7.1 Hz, 1H), 7.53 (dd, *J* = 12.9, 7.5 Hz, 2H), 6.83 (t, *J* = 8.1 Hz, 2H), 4.75 (s, 2H).

**^13^C NMR** (151 MHz, CDCl_3_) δ 171.2, 167.8, 161.8 (d, *J* = 251.0 Hz), 159.6 (ddd, *J* = 254.2, 15.1, 7.3 Hz), 142.0, 132.3, 131.2, 128.4, 124.6, 122.9, 101.2 (t, *J* = 27.3 Hz), 51.6.

**^19^F NMR** (565 MHz, CDCl_3_) δ -107.0, -113.7.

**FTMS (ESI)** m/z: [M+H]^+^ calcd for C_14_H_9_F_3_NO^+^ 264.0631; found 264.0629.


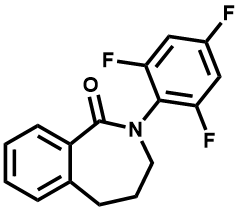
**2-(2,4,6-trifluorophenyl)-2,3,4,5-tetrahydro-1H-benzo[c]azepin-1-one (3k)**

According to the general procedure A, the product **3k** was obtained after silica gel chromatography (PE/EA: 10/1).

White solid; 26.2 mg, 45% yield; M.p. = 173-175ºC.

**^1^H NMR** (400 MHz, CDCl_3_) δ 7.75 (d, *J* = 7.5 Hz, 1H), 7.44 (t, *J* = 7.5 Hz, 1H), 7.37 (t, *J* = 7.5 Hz, 1H), 7.22 (d, *J* = 7.4 Hz, 1H), 6.80 (t, *J* = 8.0 Hz, 2H), 3.53 (t, *J* = 6.4 Hz, 2H), 3.03 (t, *J* = 7.1 Hz, 2H), 2.14 – 2.04 (m, 2H).

**^13^C NMR** (151 MHz, CDCl_3_) δ 171.1, 161.4 (dt, *J* = 29.4, 14.4 Hz), 159.2 (ddd, *J* = 22.2, 15.3, 7.3 Hz), 138.0, 135.1, 131.6, 129.7, 129.2, 128.6, 116.4, 101.0 (t, *J* = 27.6 Hz), 49.3, 30.1, 29.2.

**^19^F NMR** (376 MHz, CDCl_3_) δ -107.9, -114.2.

**FTMS (ESI)** m/z: [M+H]^+^ calcd for C_16_H_13_F_3_NO^+^ 292.0944; found 292.0945.


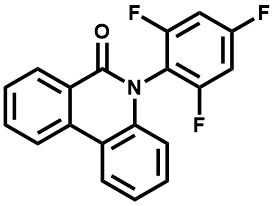
**5-(2,4,6-trifluorophenyl)phenanthridin-6(5H)-one (3l)**

According to the general procedure A, the product **3l** was obtained after silica gel chromatography (PE/EA: 5/1).

White solid; 24.1 mg, 37% yield; M.p. = 126-128ºC.

**^1^H NMR** (600 MHz, CDCl_3_) δ 8.55 (d, *J* = 7.9 Hz, 1H), 8.34 (t, *J* = 8.9 Hz, 2H), 7.84 (t, *J* = 7.7 Hz, 1H), 7.63 (t, *J* = 7.5 Hz, 1H), 7.40 (t, *J* = 7.8 Hz, 1H), 7.35 (t, *J* = 7.5 Hz, 1H), 6.95 (t, *J* = 7.9 Hz, 2H), 6.77 (d, *J* = 8.2 Hz, 1H).

**^13^C NMR** (151 MHz, CDCl_3_) δ 162.8 (d, *J* = 252.4 Hz), 167.0, 159.7 (d, *J* = 262.1 Hz), 137.5, 134.3, 133.4, 129.9, 129.7, 129.2, 128.3, 125.2, 123.5, 123.5, 122.0, 119.4, 115.3, 101.6 (t, *J* = 27.1 Hz).

**^19^F NMR** (565 MHz, CDCl_3_) δ -105.0, -113.9.

**FTMS (ESI)** m/z: [M+H]^+^ calcd for C_19_H_11_F_3_NO^+^ 326.0787; found 326.0792.


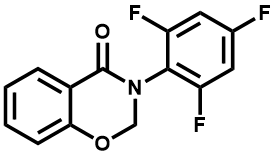
**3-(2,4,6-trifluorophenyl)-2,3-dihydro-4H-benzo[e][1,3]oxazin-4-one (3m)**

According to the general procedure A, the product **3m** was obtained after silica gel chromatography (PE/EA: 10/1).

White solid; 26.2 mg, 47% yield; M.p. = 103-104ºC.

**^1^H NMR** (600 MHz, CDCl_3_) δ 8.05 (dd, *J* = 7.8, 1.6 Hz, 1H), 7.53 (ddd, *J* = 8.3, 7.5, 1.7 Hz, 1H), 7.18 (td, *J* = 7.8, 0.9 Hz, 1H), 7.07 (d, *J* = 8.7 Hz, 1H), 6.81 (td, *J* = 9.6, 2.0 Hz, 2H), 5.44 (s, 2H).

**^13^C NMR** (151 MHz, CDCl_3_) δ 161.9 (d, *J* = 236.7 Hz), 161.1, 159.1 (d, *J* = 238.2 Hz), 158.3, 134.9, 129.1, 123.1, 118.3, 116.8, 112.7, 101.1 (t, *J* = 27.4 Hz), 79.8.

**^19^F NMR** (565 MHz, CDCl_3_) δ -106.5, -113.1.

**FTMS (ESI)** m/z: [M+H]^+^ calcd for C_14_H_9_F_3_NO_2_^+^ 280.0580; found 280.0577.


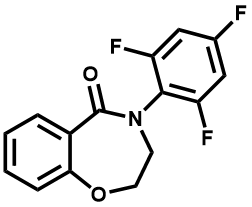
**4-(2,4,6-trifluorophenyl)-3,4-dihydrobenzo[f][1,4]oxazepin-5(2H)-one (3n)**

According to the general procedure A, the product **3n** was obtained after silica gel chromatography (PE/EA: 10/1).

White solid; 25.2 mg, 43% yield; M.p. = 108-109ºC.

**^1^H NMR** (600 MHz, CDCl_3_) δ 7.91 (dd, *J* = 7.8, 1.7 Hz, 1H), 7.48 (ddd, *J* = 8.1, 7.4, 1.7 Hz, 1H), 7.19 (td, *J* = 7.8, 1.1 Hz, 1H), 7.08 (dd, *J* = 8.2, 0.9 Hz, 1H), 6.81 (td, *J* = 9.6, 2.1 Hz, 2H), 4.51 (t, *J* = 5.0 Hz, 2H), 3.83 (t, *J* = 5.0 Hz, 2H).

**^13^C NMR** (151 MHz, CDCl_3_) δ 168.1, 161.6 (d, *J* = 250.9 Hz), 158.9 (ddd, *J* = 252.7, 15.2, 7.3 Hz), 154.4, 133.7, 131.8, 125.3, 123.5, 121.5, 116.4, 101.0 (t, *J* = 27.5 Hz), 72.4, 49.6.

**^19^F NMR** (565 MHz, CDCl_3_) δ -107.3, -114.0.

**FTMS (ESI)** m/z: [M+H]^+^ calcd for C_15_H_11_F_3_NO_2_^+^ 294.0736; found 294.0732.


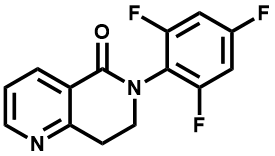
**6-(2,4,6-trifluorophenyl)-7,8-dishydro-1,6-naphthyridin-5(6H)-one (3o)**

According to the general procedure A, the product **3o** was obtained after silica gel chromatography (MeOH/DCM: 1/10).

White solid; 36.7 mg, 66% yield; M.p. = 101-103ºC.

**^1^H NMR** (400 MHz, CDCl_3_) δ 8.68 (dd, *J* = 4.9, 1.6 Hz, 1H), 8.40 (dd, *J* = 7.8, 1.6 Hz, 1H), 7.36 (dd, *J* = 7.8, 4.9 Hz, 1H), 6.81 (t, *J* = 8.1 Hz, 2H), 3.95 (t, *J* = 6.6 Hz, 2H), 3.39 (t, *J* = 6.6 Hz, 2H).

**^13^C NMR** (101 MHz, CDCl_3_) δ 163.2, 162.9, 158.8 (d, *J* = 238.0 Hz), 158.4, 152.7, 136.6, 124.5, 122.8, 115.6, 101.1 (t, *J* = 26.4 Hz), 48.3, 31.3.

**^19^F NMR** (376 MHz, CDCl_3_) δ -107.2, -114.1.

**FTMS (ESI)** m/z: [M+H]^+^ calcd for C_14_H_10_F_3_N2O^+^ 279.0740; found 274.0745.


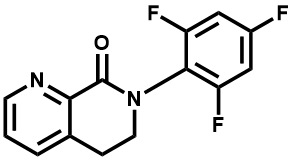
**7-(2,4,6-trifluorophenyl)-6,7-dihydro-1,7-naphthyridin-8(5H)-one (3p)**

According to the general procedure A, the product **3p** was obtained after silica gel chromatography (MeOH/DCM: 1/10).

White solid; 33.9 mg, 61% yield; M.p. = 115-117ºC.

**^1^H NMR** (600 MHz, CDCl_3_) δ 8.12 (d, *J* = 3.4 Hz, 1H), 7.55 (d, *J* = 8.1 Hz, 1H), 6.98 (dd, *J* = 7.4, 4.9 Hz, 1H), 6.81 (dd, *J* = 8.7, 7.3 Hz, 2H), 3.08 (t, *J* = 7.0 Hz, 2H), 2.90 (t, *J* = 7.8 Hz, 2H).

**^13^C NMR** (151 MHz, CDCl_3_) δ 169.7, 162.2 (dt, *J* = 250.3, 14.8 Hz), 159.4 (ddd, *J* = 252.7, 15.4, 7.5 Hz), 151.6, 146.7, 136.2, 120.1, 119.3, 111.1, 100.9 (t, *J* = 27.3 Hz), 31.3, 23.9.

^19^F NMR (565 MHz, CDCl_3_) δ -107.0, -114.5.

**FTMS (ESI)** m/z: [M+H]^+^ calcd for C_14_H_10_F_3_N2O^+^ 279.0740; found 274.0741.


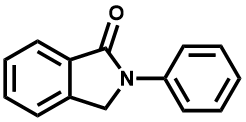
**2-phenylisoindolin-1-one (3q)**

According to the general procedure A, the product **3q** was obtained after silica gel chromatography (PE/EA: 20/1).

White solid; 14.6 mg, 35% yield; M.p. = 158-159ºC.

**^1^H NMR** (600 MHz, CDCl_3_) δ 7.93 (d, *J* = 7.5 Hz, 1H), 7.87 (d, *J* = 7.8 Hz, 2H), 7.60 (t, *J* = 7.0 Hz, 1H), 7.55 – 7.49 (m, 2H), 7.43 (t, *J* = 7.5 Hz , 2H), 7.19 (t, *J* = 7.4 Hz, 1H), 4.87 (s, 2H).

^13^C NMR (151 MHz, CDCl_3_) δ 167.5, 140.1, 139.5, 133.3, 132.1, 129.2, 128.4, 124.5, 124.2, 122.6, 119.5, 50.8.

**FTMS (ESI)** m/z: [M+H]^+^ calcd for C_14_H_12_NO^+^ 210.0913; found 210.0911.


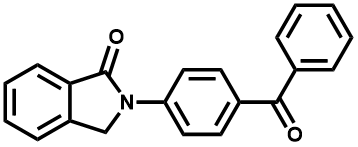
**2-(4-benzoylphenyl)isoindolin-1-one (3r)**

According to the general procedure A, the product **3r** was obtained after silica gel chromatography (PE/EA: 5/1).

White solid; 28.8 mg, 46% yield; M.p. = 149-151ºC.

**^1^H NMR** (400 MHz, CDCl_3_) δ 7.86 (d, *J* = 7.2 Hz, 2H), 7.75 (d, *J* = 7.6 Hz, 1H), 7.62 (td, *J* = 7.8, 1.5 Hz, 1H), 7.57 – 7.49 (m, 2H), 7.49 – 7.34 (m, 7H), 4.84 (s, 2H).

**^13^C NMR** (101 MHz, CDCl_3_) δ 195.9, 167.8, 141.2, 136.9, 136.8, 136.4, 132.9, 131.9, 131.6, 130.1, 128.2, 128.1, 126.6, 126.5, 124.3, 122.6, 53.1.

**FTMS (ESI)** m/z: [M+H]^+^ calcd for C_21_H_16_NO_2_^+^ 314.1176; found 314.1176.


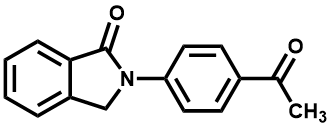
**2-(4-acetylphenyl)isoindolin-1-one (3s)**

According to the general procedure A, the product **3s** was obtained after silica gel chromatography (PE/EA: 10/1).

White solid; 24.1 mg, 48% yield; M.p. = 236-237ºC.

**^1^H NMR** (400 MHz, CDCl_3_) δ 8.08 – 7.98 (m, 4H), 7.93 (d, *J* = 7.5 Hz, 1H), 7.63 (t, *J* = 7.3 Hz, 1H), 7.58 – 7.48 (m, 2H), 4.90 (s, 2H), 2.60 (s, 3H).

**^13^C NMR** (101 MHz, CDCl_3_) δ 197.0, 167.9, 143.7, 139.9, 132.7, 132.7, 132.7, 129.8, 128.7, 124.4, 122.8, 118.1, 50.5, 26.5.

**FTMS (ESI)** m/z: [M+H]^+^ calcd for C_16_H_14_NO_2_^+^ 252.1019; found 252.1018.


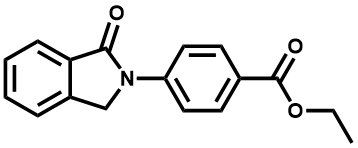
**ethyl 4-(1-oxoisoindolin-2-yl)benzoate (3t)**

According to the general procedure A, the product **3t** was obtained after silica gel chromatography (PE/EA: 5/1).

White solid; 27.0 mg, 48% yield; M.p. = 183-185ºC.

**^1^H NMR** (400 MHz, CDCl_3_) δ 8.09 (d, *J* = 9.0 Hz, 2H), 7.98 (d, *J* = 9.0 Hz, 2H), 7.91 (d, *J* = 7.5 Hz, 1H), 7.62 (t, *J* = 7.9 Hz, 1H), 7.57 – 7.46 (m, 2H), 4.87 (s, 2H), 4.38 (q, *J* = 7.1 Hz, 2H), 1.41 (t, *J* = 7.1 Hz, 3H).

**^13^C NMR** (101 MHz, CDCl_3_) δ 167.8, 166.2, 143.4, 139.9, 132.8, 132.6, 130.8, 128.6, 125.8, 124.3, 122.7, 118.0, 60.9, 50.5, 14.4.

**FTMS (ESI)** m/z: [M+H]^+^ calcd for C_17_H_16_NO_3_^+^ 282.1125; found 282.1129.


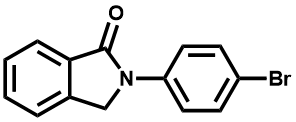
**2-(4-bromophenyl)isoindolin-1-one (3u)**

According to the general procedure A, the product **3u** was obtained after silica gel chromatography (PE/EA: 10/1).

White solid; 25.8 mg, 45% yield; M.p. = 181-182ºC.

**^1^H NMR** (600 MHz, CDCl_3_) δ 7.91 (d, J = 8.2 Hz, 1H), 7.78 (d, J = 9.0 Hz, 2H), 7.61 (t, J = 7.9 Hz, 1H), 7.56 – 7.45 (m, 4H), 4.82 (s, 2H).

**^13^C NMR** (151 MHz, CDCl_3_) δ 167.5, 139.9, 138.6, 132.9, 132.3, 132.1, 128.5, 124.2, 122.7, 120.7, 117.2, 50.6.

**FTMS (ESI)** m/z: [M+H]^+^ calcd for C_14_H_11_BrNO^+^ 288.0019; found 288.0013.


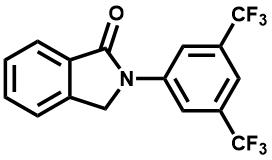
**2-(3,5-bis(trifluoromethyl)phenyl)isoindolin-1-one (3v)**

According to the general procedure A, the product **3v** was obtained after silica gel chromatography (PE/EA: 10/1).

White solid; 37.9 mg, 55% yield; M.p. = 130-131ºC.

**^1^H NMR** (600 MHz, CDCl_3_) δ 8.42 (s, 2H), 7.94 (d, *J* = 7.6 Hz, 1H), 7.66 (t, *J* = 7.4 Hz, 2H), 7.62 – 7.49 (m, 2H), 4.94 (s, 2H).

**^13^C NMR** (151 MHz, CDCl_3_) δ 167.94, 140.9, 139.6, 133.1, 132.5 (dd, *J* = 67.2, 33.7 Hz), 132.2, 128.9, 124.6, 126.4 – 120.0 (m), 122.8, 118.3 (d, *J* = 3.3 Hz), 117.7 – 116.87(m), 50.4.

**^19^F NMR** (565 MHz, CDCl_3_) δ -62.9.

**FTMS (ESI)** m/z: [M+H]^+^ calcd for C_16_H_10_F_6_NO^+^ 346.0661; found 346.0667.


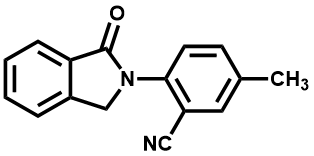
**5-methoxy-2-(1-oxoisoindolin-2-yl)benzonitrile (3w)**

According to the general procedure A, the product **3w** was obtained after silica gel chromatography (PE/EA: 5/1).

White solid; 26.9 mg, 51% yield; M.p. = 177-178ºC.

**^1^H NMR** (400 MHz, CDCl_3_) δ 7.93 (d, *J* = 7.2 Hz, 1H), 7.62 (t, *J* = 7.5 Hz, 1H), 7.52 (t, *J* = 7.2 Hz, 2H), 7.47 (dd, *J* = 7.1, 2.5 Hz, 1H), 7.21 (s, 1H), 7.21 – 7.19 (m, 1H), 4.92 (s, 2H), 3.86 (s, 3H).

**^13^C NMR** (101 MHz, CDCl_3_) δ 168.3, 158.4, 141.3, 133.9, 132.4, 131.4, 129.4, 128.5, 124.5, 123.0, 120.3, 117.9, 116.7, 111.6, 56.0, 52.5.

**FTMS (ESI)** m/z: [M+H]^+^ calcd for C_16_H_13_N_2_O_2_^+^ 265.0972; found 265.0979.


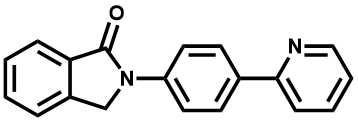
**2-(4-(pyridin-2-yl)phenyl)isoindolin-1-one (3x)**

According to the general procedure A, the product **3x** was obtained after silica gel chromatography (PE/EA: 1/1).

White solid; 24.0 mg, 42% yield; M.p. = 136-138ºC.

**^1^H NMR** (400 MHz, CDCl_3_) δ 8.68 (d, *J* = 4.8 Hz, 1H), 8.07 (d, *J* = 8.9 Hz, 2H), 8.01 (d, *J* = 8.9 Hz, 2H), 7.94 (d, *J* = 7.5 Hz, 1H), 7.81 – 7.71 (m, 2H), 7.61 (t, *J* = 7.2 Hz, 1H), 7.57 – 7.47 (m, 2H), 7.22 (td, *J* = 5.2, 3.1 Hz, 1H), 4.92 (s, 2H).

**^13^C NMR** (101 MHz, CDCl_3_) δ 167.7, 156.7, 149.7, 140.2, 140.1, 136.8, 135.2, 133.2, 132.3, 128.5, 127.7, 124.2, 122.7, 122.0, 120.3, 119.2, 50.9.

**FTMS (ESI)** m/z: [M+H]^+^ calcd for C_19_H_15_N_2_O^+^ 287.1179; found 287.1180.


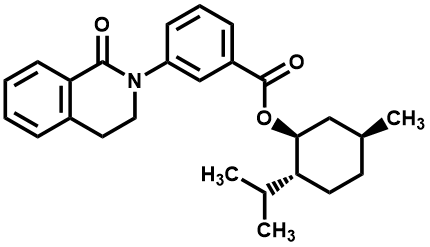
**(1S,2R,5S)-2-isopropyl-5-methylcyclohexyl 3-(1-oxo-3,4-dihydroisoquinolin-2(1H)-yl)benzoate (3y)**

According to the general procedure A, the product **3y** was obtained after silica gel chromatography (PE/EA: 10/1).

White solid; 27.5 mg, 34% yield; M.p. = 175-176ºC.

**^1^H NMR** (600 MHz, CDCl3) δ 8.16 (d, *J* = 7.7 Hz, 1H), 8.00 (s, 1H), 7.94 (d, *J* = 7.7 Hz, 1H), 7.66 (d, *J* = 8.0 Hz, 1H), 7.48 (t, *J* = 7.7 Hz, 2H), 7.39 (t, *J* = 7.6 Hz, 1H), 7.26 (d, *J* = 5.5 Hz, 1H), 4.95 (td, *J* = 10.9, 4.4 Hz, 1H), 4.03 (td, *J* = 6.5, 3.2 Hz, 2H), 3.17 (t, *J* = 6.3 Hz, 2H), 2.11 (d, *J* = 12.0 Hz, 1H), 1.97 – 1.91 (m, 1H), 1.73 (d, *J* = 11.6 Hz, 2H), 1.60 – 1.48 (m, 2H), 1.20 – 1.05 (m, 2H), 0.98 – 0.87 (m, 7H), 0.79 (d, *J* = 6.9 Hz, 3H).

**^13^C NMR** (151 MHz, CDCl3) δ 165.6, 164.3, 143.2, 138.3, 132.2, 131.9, 130.3, 129.5, 128.8, 128.8, 127.4, 127.3, 127.0, 125.8, 75.1, 49.3, 47.2, 40.9, 34.3, 31.5, 28.6, 26.5, 23.6, 22.0, 20.8, 16.5.

**FTMS (ESI)** m/z: [M+H]^+^ calcd for C_26_H_32_NO_3_^+^ 406.2377; found 406.2381.


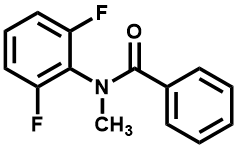
**N-(2,6-difluorophenyl)-N-methylbenzamide (4a)**

According to the general procedure A, the product **4a** was obtained after silica gel chromatography (PE/EA: 20/1).

Colorless oil; 25.2 mg, 51% yield.

**^1^H NMR** (400 MHz, DMSO) δ 7.36 – 7.29 (m, 2H), 7.25 (d, *J* = 4.4 Hz, 4H), 7.09 (t, *J* = 8.3 Hz, 2H), 3.27 (s, 3H).

**^13^C NMR** (101 MHz, DMSO) δ 170.9, 158.0 (dd, *J* = 249.1, 4.2 Hz), 135.5, 130.74, 130.6 (t, *J* = 10.1 Hz), 128.4, 127.2, 121.8 (t, *J* = 16.0 Hz), 112.8 (dd, *J* = 18.4, 4.9 Hz), 36.5.

**^19^F NMR** (376 MHz, DMSO) δ -119.8.

**FTMS (ESI)** m/z: [M+H]^+^ calcd for C_14_H_12_F_2_NO^+^ 248.0881; found 248.0876.


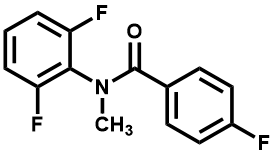
**N-(2,6-difluorophenyl)-4-fluoro-N-methylbenzamide (4b)**

According to the general procedure A, the product **4b** was obtained after silica gel chromatography (PE/EA: 20/1).

Colorless oil; 26.5 mg, 50% yield.

**^1^H NMR** (400 MHz, DMSO) δ 7.34 (dd, *J* = 8.5, 5.5 Hz, 2H), 7.21 – 7.10 (m, 1H), 6.83 (dt, *J* = 11.9, 8.4 Hz, 4H), 3.36 (s, 3H).

**^13^C NMR** (101 MHz, DMSO) δ 165.9, 158.8 (d, *J* = 250.4 Hz), 153.4 (dd, *J* = 251.4, 4.1 Hz), 126.7 (d, *J* = 3.0 Hz), 124.9 (d, *J* = 8.7 Hz), 124.4 (t, *J* = 9.9 Hz), 117.3 (t, *J* = 15.9 Hz), 110.1 (d, *J* = 21.9 Hz), 107.3 (dd, *J* = 18.8, 5.0 Hz), 31.7.

**^19^F NMR** (376 MHz, DMSO) δ -114.2, -123.6.

**FTMS (ESI)** m/z: [M+H]^+^ calcd for C_14_H_11_F_3_NO^+^ 266.0787; found 266.0797.


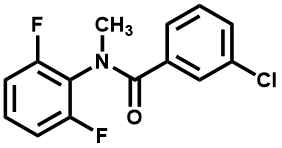
**3-chloro-N-(2,6-difluorophenyl)-N-methylbenzamide (4c)**

According to the general procedure A, the product **4c** was obtained after silica gel chromatography (PE/EA: 20/1).

Colorless oil; 25.3 mg, 45% yield.

**^1^H NMR** (400 MHz, CDCl_3_) δ 7.36 (s, 1H), 7.24 – 7.12 (m, 3H), 7.08 (t, *J* = 7.8 Hz, 1H), 6.83 (t, *J* = 8.0 Hz, 2H), 3.36 (s, 3H).

**^13^C NMR** (101 MHz, CDCl_3_) δ 170.1, 158.1 (dd, *J* = 251.6, 4.0 Hz), 137.0, 133.9, 130.2, 129.5 (t, *J* = 9.9 Hz), 129.1, 127.6, 125.2, 121.7 (t, *J* = 16.1 Hz), 112.1 (dd, *J* = 18.8, 5.0 Hz), 36.4.

**^19^F NMR** (376 MHz, CDCl_3_) δ -118.8.

**FTMS (ESI)** m/z: [M+H]^+^ calcd for C_14_H_11_ClF_2_NO^+^ 282.0492; found 282.0492.

**4-bromo-N-(2,6-difluorophenyl)-N-methylbenzamide (4d)**


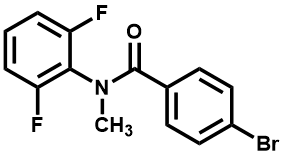


According to the general procedure A, the product **4d** was obtained after silica gel chromatography (PE/EA: 20/1).

Colorless oil; 31.2 mg, 48% yield.

**^1^H NMR** (400 MHz, DMSO) δ 7.49 (d, *J* = 8.4 Hz, 2H), 7.44 – 7.31 (m, 1H), 7.20 (d, *J* = 8.4 Hz, 2H), 7.13 (t, *J* = 8.3 Hz, 2H), 3.27 (s, 3H).

**^13^C NMR** (101 MHz, DMSO) δ 167.0, 157.9 (d, *J* = 253.0 Hz), 134.6, 131.6, 130.8 (t, *J* = 10.1 Hz), 129.4, 124.3, 121.4 (t, *J* = 16.2 Hz), 113.0 (dd, *J* = 18.5, 4.6 Hz), 36.5.

**^19^F NMR** (376 MHz, DMSO) δ -119.8.

**FTMS (ESI)** m/z: [M+H]^+^ calcd for C_14_H_11_BrF_2_NO^+^ 325.9987; found 325.9989.


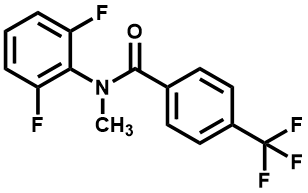
**N-(2,6-difluorophenyl)-N-methyl-4-(trifluoromethyl)benzamide (4e)**

According to the general procedure A, the product **4e** was obtained after silica gel chromatography (PE/EA: 20/1).

Colorless oil; 30.9 mg, 49% yield.

**^1^H NMR** (400 MHz, CDCl_3_) δ 7.45 (s, 4H), 7.17 (tt, *J* = 8.5, 6.2 Hz, 1H), 6.83 (t, *J* = 8.0 Hz, 2H), 3.39 (s, 3H).

**^13^C NMR** (101 MHz, CDCl_3_) δ 170.2, 158.1 (d, *J* = 251.5 Hz), 138.8, 131.8 (d, *J* = 32.8 Hz), 129.6 (t, *J* = 9.9 Hz), 127.6, 124.9 (d, *J* = 3.7 Hz), 122.2, 121.5, 112.2 (d, *J* = 23.7 Hz), 36.4.

**^19^F NMR** (376 MHz, CDCl_3_) δ -63.0, -118.8.

**FTMS (ESI)** m/z: [M+H]^+^ calcd for C_15_H_11_F_5_NO^+^ 316.0755; found 316.0749.

**methyl 4-((2,6-difluorophenyl)(methyl)carbamoyl)benzoate (4f)**


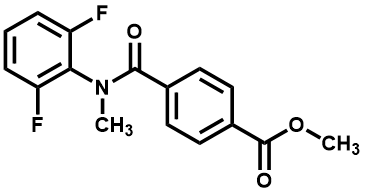


According to the general procedure A, the product **4f** was obtained after silica gel chromatography (PE/EA: 10/1).

White solid; 28.7 mg, 47% yield; M.p. = 75-77ºC.

**^1^H NMR** (400 MHz, DMSO) δ 7.82 (d, *J* = 8.3 Hz, 2H), 7.37 (d, *J* = 8.2 Hz, 2H), 7.35 – 7.30 (m, 1H), 7.10 (t, *J* = 8.3 Hz, 2H), 3.81 (s, 3H), 3.30 (s, 3H).

**^13^C NMR** (151 MHz, DMSO) δ 170.0, 166.0, 158.0 (d, *J* = 249.3 Hz), 139.8, 131.4, 131.0 (t, *J* = 10.2 Hz), 129.3, 127.5, 121.1 (t, *J* = 16.1 Hz), 112.9 (dd, *J* = 19.7, 3.5 Hz), 52.8, 36.4.

**^19^F NMR** (376 MHz, DMSO) δ -119.7.

**FTMS (ESI)** m/z: [M+H]^+^ calcd for C_16_H_14_F_2_NO_3_^+^ 306.0936; found 306.0937.


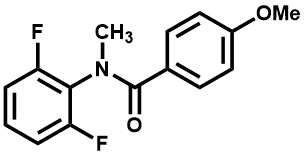
**N-(2,6-difluorophenyl)-4-methoxy-N-methylbenzamide (4g)**

According to the general procedure A, the product **4g** was obtained after silica gel chromatography (PE/EA: 20/1).

Colorless oil; 29.4 mg, 53% yield.

**^1^H NMR** (400 MHz, CDCl_3_) δ 7.31 (d, *J* = 8.6 Hz, 2H), 7.20 – 7.03 (m, 1H), 6.82 (t, *J* = 8.1 Hz, 2H), 6.67 (d, *J* = 8.6 Hz, 2H), 3.71 (s, 3H), 3.35 (s, 3H).

**^13^C NMR** (101 MHz, CDCl_3_) δ 171.3, 161.0, 158.1 (d, *J* = 246.8 Hz), 129.4, 128.8 (t, *J* = 9.8 Hz), 127.4, 122.6 (t, *J* = 15.8 Hz), 113.0, 112.0 (dd, *J* = 18.7, 5.1 Hz), 55.1, 36.5.

**^19^F NMR** (376 MHz, CDCl_3_) δ -119.0.

**FTMS (ESI)** m/z: [M+H]^+^ calcd for C_15_H_14_F_2_NO_2_^+^ 278.0987; found 278.0987.


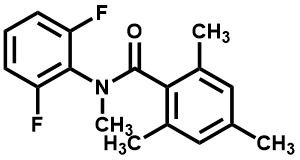
**N-(2,6-difluorophenyl)-N,2,4,6-tetramethylbenzamide (4h)**

According to the general procedure A, the product **4h** was obtained after silica gel chromatography (PE/EA: 20/1).

Colorless oil; 30.1 mg, 52% yield.

**^1^H NMR** (400 MHz, DMSO) δ 7.57 – 7.44 (m, 1H), 7.27 (t, *J* = 8.3 Hz, 2H), 6.97 (s, 2H), 2.98 (s, 3H), 2.28 (s, 3H), 2.26 (s, 6H).

**^13^C NMR** (151 MHz, DMSO) δ 170.8, 159.0 (dd, *J* = 249.5, 5.3 Hz), 138.4, 133.5, 133.3, 130.3 (t, *J* = 9.9 Hz), 128.6, 119.2 (t, *J* = 17.1 Hz), 112.8 (dd, *J* = 19.6, 3.7 Hz), 37.7, 21.2, 18.7.

**^19^F NMR** (565 MHz, DMSO) δ -119.0.

**FTMS (ESI)** m/z: [M+H]^+^ calcd for C_17_H_18_F_2_NO^+^ 290.1351; found 290.1350.


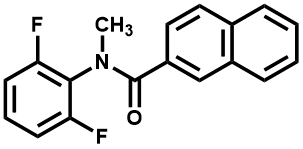
**N-(2,6-difluorophenyl)-N-methyl-2-naphthamide (4i)**

According to the general procedure A, the product **4i** was obtained after silica gel chromatography (PE/EA: 20/1).

White solid; 25.5 mg, 43% yield; M.p. = 79-80ºC.

**^1^H NMR** (400 MHz, CDCl_3_) δ 7.87 (s, 1H), 7.73 (d, *J* = 7.8 Hz, 2H), 7.64 (d, *J* = 8.6 Hz, 1H), 7.44 (dd, *J* = 12.4, 8.2 Hz, 3H), 7.08 (ddd, *J* = 14.6, 8.3, 6.3 Hz, 1H), 6.78 (t, *J* = 8.1 Hz, 2H), 3.44 (s, 3H).

**^13^C NMR** (151 MHz, CDCl_3_) δ 171.7, 158.2 (d, *J* = 247.5 Hz), 133.8, 132.7, 132.3, 129.0 (t, *J* = 9.9 Hz), 128.6, 127.8, 127.6, 127.5, 127.1, 126.3, 124.2, 122.2 (t, *J* = 15.9 Hz), 112.0 (dd, *J* = 19.9, 3.9 Hz), 36.6.

**^19^F NMR** (565 MHz, CDCl_3_) δ -118.7.

**FTMS (ESI)** m/z: [M+H]^+^ calcd for C_18_H_14_F_2_NO^+^ 298.1038; found 298.1034.


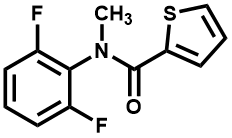
**N-(2,6-difluorophenyl)-N-methylthiophene-2-carboxamide (4j)**

According to the general procedure A, the product **4j** was obtained after silica gel chromatography (PE/EA: 20/1).

Colorless oil; 25.3 mg, 50% yield.

**^1^H NMR** (400 MHz, CDCl_3_) δ 7.38 – 7.29 (m, 2H), 7.01 (d, *J* = 3.2 Hz, 1H), 6.97 (t, *J* = 8.0 Hz, 2H), 6.82 (t, *J* = 4.2 Hz, 1H), 3.36 (s, 3H).

**^13^C NMR** (101 MHz, CDCl_3_) δ 163.4, 159.2 (dd, *J* = 252.7, 3.8 Hz), 136.6, 131.3, 130.4, 129.9 (t, *J* = 9.9 Hz), 126.8, 121.5, 112.5 (d, *J* = 23.4 Hz), 37.1.

**^19^F NMR** (376 MHz, CDCl_3_) δ -118.7.

**FTMS (ESI)** m/z: [M+H]^+^ calcd for C_12_H_10_F_2_NOS^+^ 254.0446; found 254.0445.


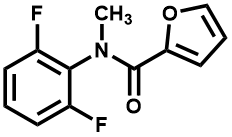
**N-(2,6-difluorophenyl)-N-methylfuran-2-carboxamide (4k)**

According to the general procedure A, the product **4k** was obtained after silica gel chromatography (PE/EA: 20/1).

Colorless oil; 24.2 mg, 51% yield.

**^1^H NMR** (400 MHz, CDCl_3_) δ 7.30 (td, *J* = 8.4, 4.2 Hz, 1H), 7.22 (s, 1H), 6.96 (t, *J* = 7.5 Hz ,2H), 6.36 (d, *J* = 3.3 Hz, 1H), 6.25 (d, *J* = 1.7 Hz, 1H), 3.33 (s, 3H).

**^13^C NMR** (101 MHz, CDCl_3_) δ 159.6, 159.2 (dd *J* = 300, 12 Hz), 146.9, 144.6, 129.4 (t, *J* = 9.8 Hz), 121.2, 115.9, 112.1 (dd, *J* = 18.4, 5.2 Hz), 111.1, 36.7.

**^19^F NMR** (376 MHz, CDCl_3_) δ -119.6.

**FTMS (ESI)** m/z: [M+H]^+^ calcd for C_12_H_10_F_2_NO_2_^+^ 238.0674; found 238.0679.


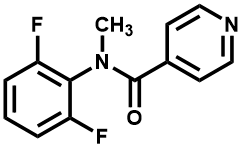
**N-(2,6-difluorophenyl)-N-methylisonicotinamide (4l)**

According to the general procedure A, the product **4l** was obtained after silica gel chromatography (PE/EA: 1/1).

Colorless oil; 22.8 mg, 46% yield.

**^1^H NMR** (400 MHz, MeOD) δ 8.45 (dd, *J* = 4.6, 1.4 Hz, 2H), 7.34 (ddd, *J* = 8.6, 6.4, 2.2 Hz, 1H), 7.29 (d, *J* = 6.0 Hz, 2H), 6.99 (t, *J* = 8.2 Hz, 2H), 3.38 (s, 3H).

**^13^C NMR** (101 MHz, MeOD) δ 169.5, 158.2 (d, *J* = 246.4 Hz), 149.0 , 143.6, 137.0 (t, *J* = 18.0 Hz), 130.7 (t, *J* = 10.2 Hz), 121.2, 112.1 (dd, *J* = 19.1, 4.8 Hz), 35.2.

**^19^F NMR** (376 MHz, MeOD) δ -121.1.

**FTMS (ESI)** m/z: [M+H]^+^ calcd for C_13_H_11_F_2_N_2_O^+^ 249.0834; found 249.0840.


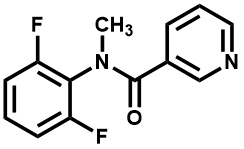
**N-(2,6-difluorophenyl)-N-methylnicotinamide (4m)**

According to the general procedure A, the product **4m** was obtained after silica gel chromatography (PE/EA: 1/1).

Colorless oil; 22.3 mg, 45% yield.

**^1^H NMR** (400 MHz, MeOD) δ 8.47 (dd, *J* = 7.1, 1.9 Hz, 2H), 7.76 (dt, *J* = 7.9, 1.7 Hz, 1H), 7.53 – 7.23 (m, 2H), 7.00 (t, *J* = 8.3 Hz, 2H), 3.38 (s, 3H).

**^13^C NMR** (101 MHz, MeOD) δ 169.4, 158.0 (dd, *J* = 250.3, 4.0 Hz), 150.4, 147.0, 135.3, 131.6, 130.4 (t, *J* = 10.1 Hz), 123.3, 120.8 (t, *J* = 16.3 Hz), 112.1 (dd, *J* = 19.2, 4.7 Hz), 35.4.

**^19^F NMR** (376 MHz, MeOD) δ -121.2.

**FTMS (ESI)** m/z: [M+H]^+^ calcd for C_13_H_11_F_2_N_2_O^+^ 249.0834; found 249.0834.


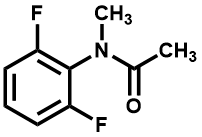
**N-(2,6-difluorophenyl)-N-methylacetamide (4n)**

According to the general procedure A, the product **4n** was obtained after silica gel chromatography (PE/EA: 20/1).

Colorless oil; 19.6 mg, 53% yield.

**^1^H NMR** (400 MHz, CDCl_3_) δ 7.39 – 7.27 (m, 1H), 7.00 (dd, *J* = 8.3, 7.4 Hz, 2H), 3.18 (s, 3H), 1.86 (s, 3H).

**^13^C NMR** (101 MHz, CDCl_3_) δ 170.8, 158.8 (dd, *J* = 252.0, 4.2 Hz), 129.5 (t, *J* = 9.8 Hz), 121.2 (t, *J* = 16.6 Hz), 112.4 (dd, *J* = 18.7, 5.2 Hz), 35.5, 21.2.

**^19^F NMR** (376 MHz, CDCl_3_) δ -118.8.

**FTMS (ESI)** m/z: [M+H]^+^ calcd for C_9_H_10_F_2_NO^+^ 186.0725; found 186.0721.


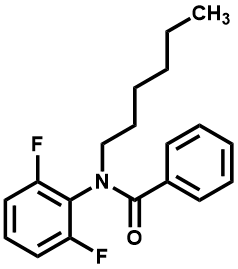
**N-(2,6-difluorophenyl)-N-hexylbenzamide (4o)**

According to the general procedure A, the product **4o** was obtained after silica gel chromatography (PE/EA: 20/1).

Colorless oil; 31.1 mg, 49% yield.

**^1^H NMR** (400 MHz, CDCl_3_) δ 7.32 (d, *J* = 7.2 Hz, 2H), 7.23 (d, *J* = 7.3 Hz, 1H), 7.19 – 7.08 (m, 3H), 6.79 (t, *J* = 8.0 Hz, 2H), 3.80 (t, *J* = 7.9 Hz, 2H), 1.65 – 1.58 (m, 2H), 1.33 – 1.23 (m, 6H), 0.92 – 0.81 (m, 3H).

**^13^C NMR** (151 MHz, CDCl_3_) δ 171.3, 158.6 (d, *J* = 251.3 Hz), 135.9, 129.8, 129.0 (t, *J* = 9.9 Hz), 127.7, 127.1, 120.9 (t, *J* = 16.2 Hz), 112.5 – 111.3 (dd, *J* = 20.3, 3.9 Hz), 49.0, 31.6, 27.4, 26.5, 22.6, 14.0.

**^19^F NMR** (376 MHz, CDCl_3_) δ -117.3.

**FTMS (ESI)** m/z: [M+H]^+^ calcd for C_19_H_22_F_2_NO^+^ 318.1664; found 318.1672.


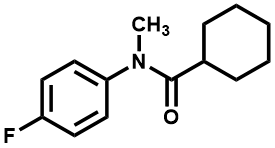
**N-(4-fluorophenyl)-N-methylcyclohexanecarboxamide(4p)**

According to the general procedure A, the product **4p** was obtained after silica gel chromatography (PE/EA: 20/1).

Colorless oil; 14.6 mg, 31% yield.

**^1^H NMR** (400 MHz, CDCl_3_) δ 7.25 – 7.03 (m, 4H), 3.22 (s, 3H), 2.16 (t, *J* = 11.4 Hz, 1H), 1.65 (t, *J* = 13.3 Hz, 3H), 1.59 – 1.42 (m, 3H), 1.38 – 1.12 (m, 2H), 0.99 (t, *J* = 12.8 Hz, 2H).

**^13^C NMR** (101 MHz, CDCl_3_) δ 176.6, 161.6 (d, *J* = 247.8 Hz), 140.2 (d, *J* = 3.3 Hz), 128.9 (d, *J* = 8.6 Hz), 116.6 (d, *J* = 22.7 Hz), 41.3, 37.5, 29.3, 28.9, 25.4.

**^19^F NMR** (376 MHz, CDCl_3_) δ -113.6.

**FTMS (ESI)** m/z: [M+H]^+^ calcd for C_14_H_19_FNO^+^ 236.1445; found 236.1445.


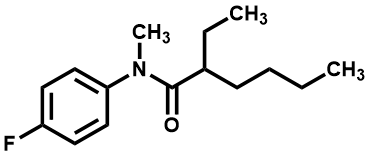
**2-ethyl-N-(4-fluorophenyl)-N-methylhexanamide (4q)**

According to the general procedure A, the product **4q** was obtained after silica gel chromatography (PE/EA: 20/1).

Colorless oil; 16.1 mg, 32% yield.

**^1^H NMR** (400 MHz, CDCl_3_) δ 7.20 – 7.03 (m, 4H), 3.26 (s, 3H), 2.31 – 2.09 (m, 1H), 1.68 – 1.48 (m, 2H), 1.46 – 1.27 (m, 2H), 1.25 – 1.04 (m, 4H), 0.87 – 0.75 (m, 6H).

**^13^C NMR** (101 MHz, CDCl_3_) δ 176.5, 161.6 (d, *J* = 247.8 Hz), 140.2 (d, *J* = 3.3 Hz), 129.6 (d, *J* = 8.5 Hz), 116.5 (d, *J* = 22.6 Hz), 43.5, 37.5, 32.7, 29.8, 26.2, 22.8, 13.9, 12.1.

**^19^F NMR** (376 MHz, CDCl_3_) δ -113.7.

**FTMS (ESI)** m/z: [M+H]^+^ calcd for C_15_H_23_FNO^+^ 252.1758; found 252.1759.


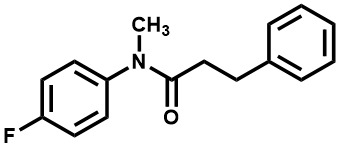
**N-(4-fluorophenyl)-N-methyl-3-phenylpropanamide (4r)**

According to the general procedure A, the product **4r** was obtained after silica gel chromatography (PE/EA: 20/1).

Colorless oil; 14.4 mg, 28% yield.

**^1^H NMR** (400 MHz, CDCl_3_) δ 7.29 – 7.12 (m, 4H), 7.08 – 6.96 (m, 4H), 6.91 (dd, *J* = 8.8, 4.9 Hz, 1H), 3.20 (s, 3H), 2.90 (t, *J* = 7.7 Hz, 2H), 2.35 (t, *J* = 7.7 Hz, 2H).

**^13^C NMR** (101 MHz, CDCl_3_) δ 172.5, 161.7 (d, *J* = 247.9 Hz), 141.1, 139.9 (d, *J* = 3.3 Hz), 129.1 (d, *J* = 8.6 Hz), 128.5, 128.4, 126.2, 116.7 (d, *J* = 22.6 Hz), 37.5, 35.9, 31.8.

**^19^F NMR** (376 MHz, CDCl_3_) δ -113.2.

**FTMS (ESI)** m/z: [M+H]^+^ calcd for C_16_H_17_FNO^+^ 258.1289; found 258.1293.

**N-(4-fluorophenyl)-N-methyl-2-phenylacetamide (4s)**


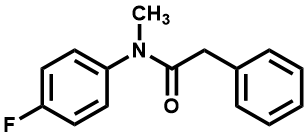


According to the general procedure A, the product **4s** was obtained after silica gel chromatography (PE/EA: 20/1).

Colorless oil; 14.1 mg, 29% yield.

**^1^H NMR** (400 MHz, CDCl_3_) δ 7.25 – 7.17 (m, 3H), 7.07 (d, *J* = 6.5 Hz, 4H), 7.03 (d, *J* = 6.9 Hz, 2H), 3.45 (s, 2H), 3.24 (s, 3H).

**^13^C NMR** (101 MHz, CDCl_3_) δ 171.1, 161.8 (d, *J* = 248.2 Hz), 139.9 (d, *J* = 3.3 Hz), 135.2, 129.4 (d, *J* = 8.6 Hz), 128.9, 128.4, 126.7, 116.6 (d, *J* = 22.7 Hz), 41.1, 37.8.

**^19^F NMR** (376 MHz, CDCl_3_) δ -113.1.

**FTMS (ESI)** m/z: [M+H]^+^ calcd for C_15_H_15_FNO^+^ 244.1132; found 244.1133.


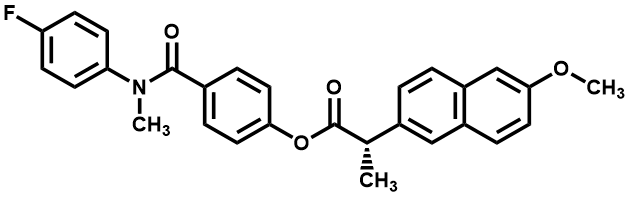
**4-((4-fluorophenyl)(methyl)carbamoyl)phenyl (S)-2-(6-methoxynaphthalen-2-yl)propanoate (4t)**

According to the general procedure A, the product **4t** was obtained after silica gel chromatography (PE/EA: 20/1).

White solid; 31.1 mg, 34% yield; M.p. = 215-217ºC.

**^1^H NMR** (400 MHz, CDCl_3_) δ 7.72 (t, *J* = 7.8 Hz, 3H), 7.44 (dd, *J* = 8.4, 1.8 Hz, 1H), 7.25 (d, *J* = 4.3 Hz, 2H), 7.19 – 7.10 (m, 2H), 7.01 – 6.95 (m, 2H), 6.94 – 6.86 (m, 2H), 6.83 (d, *J* = 8.5 Hz, 2H), 4.04 (q, *J* = 7.1 Hz, 1H), 3.91 (s, 3H), 3.43 (s, 3H), 1.65 (d, *J* = 7.1 Hz, 3H).

**^13^C NMR** (151 MHz, CDCl_3_) δ 172.6, 169.6, 160.8 (d, *J* = 247.4 Hz), 157.8, 151.7, 140.8, 134.8, 133.8, 133.0, 130.1, 129.3, 128.9, 128.4 (d, *J* = 8.5 Hz), 127.4, 126.1, 126.0, 120.9, 119.2, 116.2 (d, *J* = 22.5 Hz), 105.6, 55.3, 45.5, 38.7, 18.4.

**^19^F NMR** (376 MHz, CDCl_3_) δ -114.5.

**FTMS (ESI)** m/z: [M+H]^+^ calcd for C_28_H_25_FNO_4_^+^ 458.1762; found 458.1768.


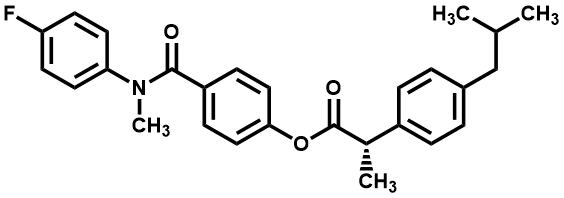
**4-((4-fluorophenyl)(methyl)carbamoyl)phenyl (S)-2-(4-isobutylphenyl)propanoate (4u)**

According to the general procedure A, the product **4u** was obtained after silica gel chromatography (PE/EA: 20/1).

White solid; 30.3 mg, 35% yield; M.p. = 191-192ºC.

**^1^H NMR** (400 MHz, CDCl_3_) δ 7.26 (dd, *J* = 9.8, 8.8 Hz, 4H), 7.12 (d, *J* = 8.1 Hz, 2H), 7.04 – 6.96 (m, 1H), 6.95 – 6.88 (m, 2H), 6.84 (d, *J* = 8.6 Hz, 1H), 3.88 (d, *J* = 7.1 Hz, 1H), 3.44 (s, 3H), 2.45 (d, *J* = 7.2 Hz, 2H), 1.85 (dt, *J* = 13.5, 6.8 Hz, 1H), 1.56 (d, *J* = 7.2 Hz, 3H), 0.90 (d, *J* = 6.6 Hz, 6H).

**^13^C NMR** (151 MHz, CDCl_3_) δ 172.7, 169.6, 160.8 (d, *J* = 247.4 Hz), 151.8, 140.9, 136.9, 132.9, 130.1, 129.5, 128.5, 128.4, 127.2, 120.9, 116.3, 116.1, 45.2, 45.0, 38.7, 30.2, 22.4, 18.4.

**^19^F NMR** (376 MHz, CDCl_3_) δ -114.6.

**FTMS (ESI)** m/z: [M+H]^+^ calcd for C_27_H_29_FNO_3_^+^ 434.2126; found 434.2126.

**N,N-diphenylbenzamide (5a)**


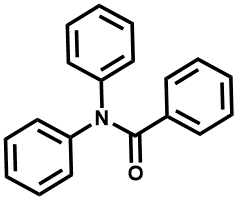


According to the general procedure A, the product **5a** was obtained after silica gel chromatography (PE/EA: 20/1).

White solid; 16.4 mg, 30% yield; M.p. = 181-182ºC.

**^1^H NMR** (400 MHz, CDCl_3_) δ 7.49 – 7.44 (m, 2H), 7.32 – 7.27 (m, 5H), 7.24 – 7.13 (m, 8H).

**^13^C NMR** (101 MHz, CDCl_3_) δ 170.6, 143.9, 136.1, 130.2, 129.2, 129.1, 127.9, 127.5, 126.3.

**FTMS (ESI)** m/z: [M+H]^+^ calcd for C_19_H_16_NO^+^ 274.1226; found 274.1225.


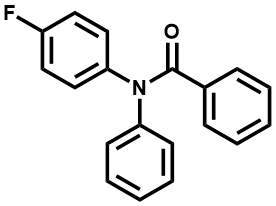
**N-(4-fluorophenyl)-N-phenylbenzamide (5b)**

According to the general procedure A, the product **5b** was obtained after silica gel chromatography (PE/EA: 20/1).

White solid; 27.4 mg, 47% yield; M.p. = 160-161ºC.

**^1^H NMR** (400 MHz, CDCl_3_) δ 7.47 – 7.41 (m, 2H), 7.33 – 7.27 (m, 3H), 7.22 (t, *J* = 7.5 Hz, 2H), 7.20 – 7.09 (m, 5H), 6.99 (t, *J* = 8.6 Hz, 2H).

**^13^C NMR** (101 MHz, CDCl_3_) δ 170.6, 161.7 (d, *J* = 52.9 Hz), 143.8, 135.8, 130.3, 129.2, 129.1, 129.0, 129.0, 127.9, 127.4, 126.5, 116.0 (d, *J* = 22.8 Hz).

**^19^F NMR** (376 MHz, CDCl_3_) δ -115.3.

**FTMS (ESI)** m/z: [M+H]^+^ calcd for C_19_H_15_FNO^+^ 292.1132; found 295.1135.

**N-(4-chlorophenyl)-N-phenylbenzamide (5c)**


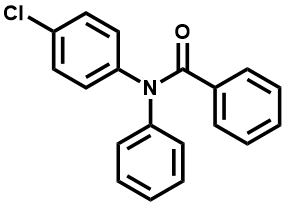


According to the general procedure A, the product **5c** was obtained after silica gel chromatography (PE/EA: 20/1).

White solid; 25.8 mg, 42% yield; M.p. = 93-95ºC.

**^1^H NMR** (400 MHz, CDCl_3_) δ 7.39 – 7.32 (m, 2H), 7.22 – 7.05 (m, 8H), 7.04 – 6.94 (m, 4H).

**^13^C NMR** (101 MHz, CDCl_3_) δ 170.6, 143.6, 142.5, 135.8, 131.9, 130.4, 129.3, 129.3, 129.2, 128.5, 128.0, 127.6, 126.7.

**FTMS (ESI)** m/z: [M+H]^+^ calcd for C_19_H_15_ClNO^+^ 308.0837; found 308.0837.


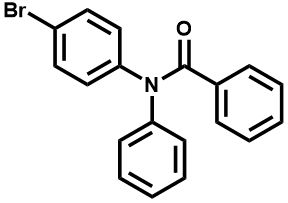
**N-(4-bromophenyl)-N-phenylbenzamide (5d)**

According to the general procedure A, the product **5d** was obtained after silica gel chromatography (PE/EA: 20/1).

White solid; 28.8 mg, 41% yield; M.p. = 115-116ºC.

**^1^H NMR** (400 MHz, CDCl_3_) δ 7.47 – 7.39 (m, 4H), 7.33 – 7.27 (m, 2H), 7.25 – 7.16 (m, 4H), 7.11 (d, *J* = 7.4 Hz, 2H), 7.04 (d, *J* = 8.7 Hz, 2H).

**^13^C NMR** (101 MHz, CDCl_3_) δ 170.5, 143.6, 143.0, 135.7, 132.2, 130.4, 129.3, 129.2, 128.8, 128.0, 127.6, 126.7, 119.8.

**FTMS (ESI)** m/z: [M+H]^+^ calcd for C_19_H_15_BrNO^+^ 352.0332; found 352.0333.


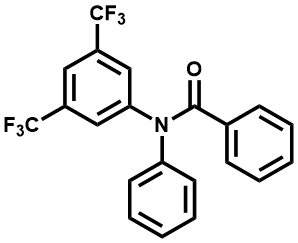
**N-(3,5-bis(trifluoromethyl)phenyl)-N-phenylbenzamide (5e)**

According to the general procedure A, the product **5e** was obtained after silica gel chromatography (PE/EA: 20/1).

White solid; 28.8 mg, 50% yield; M.p. = 123-124ºC.

**^1^H NMR** (600 MHz, CDCl_3_) δ 7.60 (s, 1H), 7.56 (s, 2H), 7.35 (d, *J* = 7.2 Hz, 2H), 7.27 – 7.16 (m, 3H), 7.16 – 7.11 (m, 3H), 6.99 (d, *J* = 7.5 Hz, 2H).

**^13^C NMR** (151 MHz, CDCl_3_) δ 170.8, 145.3, 142.6, 134.9, 132.4 (q, *J* = 33.7 Hz), 131.0, 129.8, 129.2, 128.1 (d, *J* = 5.2 Hz), 127.6, 126.6 (d, *J* = 3.0 Hz), 123.8, 122.0, 119.9 – 118.9 (m).

**^19^F NMR** (565 MHz, CDCl_3_) δ -63.0.

**FTMS (ESI)** m/z: [M+H]^+^ calcd for C_21_H_14_F_6_NO^+^ 410.0974; found 410.0981.


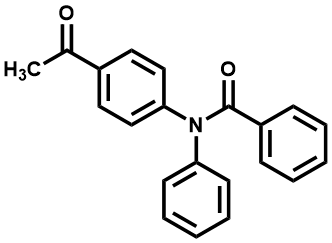
**N-(4-acetylphenyl)-N-phenylbenzamide (5f)**

According to the general procedure A, the product **5f** was obtained after silica gel chromatography (PE/EA: 10/1).

White solid; 24.5 mg, 39% yield; M.p. = 136-138ºC.

**^1^H NMR** (400 MHz, CDCl_3_) δ 7.89 (d, *J* = 8.6 Hz, 2H), 7.50 – 7.44 (m, 2H), 7.41 – 7.35 (m, 2H), 7.34 – 7.28 (m, 3H), 7.25 – 7.19 (m, 3H), 7.14 – 7.07 (m, 2H), 7.14 – 7.07 (m, 3H).

**^13^C NMR** (101 MHz, CDCl_3_) δ 197.0, 170.7, 148.2, 143.3, 135.6, 134.4, 130.6, 129.4, 129.3, 129.2, 128.4, 128.1, 127.0, 126.7, 26.6.

**FTMS (ESI)** m/z: [M+H]^+^ calcd for C_21_H_18_NO_2_^+^ 316.1332; found 316.1335.


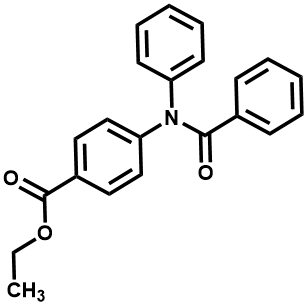
**ethyl 4-(N-phenylbenzamido)benzoate (5g)**

According to the general procedure A, the product **5g** was obtained after silica gel chromatography (PE/EA: 10/1).

White solid; 28.3 mg, 41% yield; M.p. = 144-146ºC.

**^1^H NMR** (400 MHz, CDCl_3_) δ 7.91 – 7.85 (m, 2H), 7.42 – 7.35 (m, 2H), 7.23 (ddd, *J* = 5.2, 4.6, 2.3 Hz, 3H), 7.17 – 7.10 (m, 5H), 7.08 – 7.02 (m, 2H), 4.28 (q, *J* = 7.1 Hz, 2H), 1.30 (t, *J* = 7.1 Hz, 3H).

**^13^C NMR** (101 MHz, CDCl_3_) δ 170.7, 165.9, 148.0, 143.4, 135.7, 130.6, 130.4, 129.4, 129.2, 128.0, 127.9, 127.8, 126.9, 126.7, 61.1, 14.3.

**FTMS (ESI)** m/z: [M+H]^+^ calcd for C_22_H_20_NO_3_^+^ 346.1438; found 346.1442.


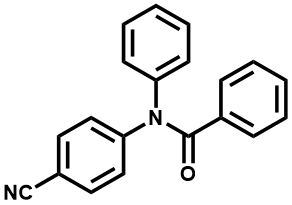
**N-(4-cyanophenyl)-N-phenylbenzamide (5h)**

According to the general procedure A, the product **5h** was obtained after silica gel chromatography (PE/EA: 10/1).

White solid; 31.0 mg, 52% yield; M.p. = 141-142ºC.

**^1^H NM**R (600 MHz, DMSO) δ 7.81 (d, *J* = 8.6 Hz, 2H), 7.48 – 7.42 (m, 2H), 7.36 (ddd, *J* = 17.7, 9.9, 4.9 Hz, 5H), 7.28 (t, *J* = 7.6 Hz, 2H), 7.25 (dd, *J* = 9.1, 8.0 Hz, 3H).

**^13^C NMR** (151 MHz, DMSO) δ 170.3, 148.2, 143.2, 136.2, 133.6, 130.8, 129.9, 129.2, 128.9, 128.4, 128.1, 127.7, 119.0, 108.7.

**FTMS (ESI)** m/z: [M+H]^+^ calcd for C_20_H_15_N_2_O^+^ 299.1179; found 299.1179.


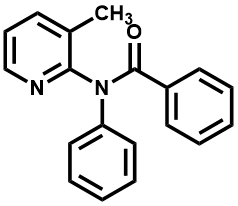
**N-(3-methylpyridin-2-yl)-N-phenylbenzamide (5i)**

According to the general procedure A, the product **5i** was obtained after silica gel chromatography (PE/EA: 3/1).

White solid; 31.1 mg, 54% yield; M.p. = 130-131ºC.

**^1^H NMR** (400 MHz, CDCl_3_) δ 8.10 (d, *J* = 7.5 Hz, 1H), 7.59 (t, *J* = 7.3 Hz, 1H), 7.47 (dd, *J* = 17.0, 8.2 Hz, 3H), 7.41 – 7.36 (m, 1H), 7.33 – 7.28 (m, 2H), 7.22 (t, *J* = 7.6 Hz, 2H), 7.19 – 7.09 (m, 3H), 1.25 (s, 3H).

**^13^C NMR** (101 MHz, CDCl_3_) δ 154.7, 147.2, 140.3, 133.3, 130.9, 130.5, 130.1, 129.1, 128.9, 128.4, 127.9, 126.3, 126.2, 123.0, 17.8.

**FTMS (ESI)** m/z: [M+H]^+^ calcd for C_19_H_17_N_2_O^+^ 289.1335; found 289.1338.


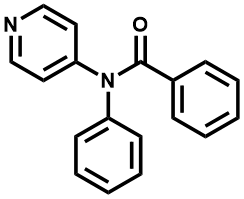
**N-phenyl-N-(pyridin-4-yl)benzamide (5j)**

According to the general procedure A, the product **5j** was obtained after silica gel chromatography (PE/EA: 3/1).

White solid; 29.0 mg, 53% yield; M.p. = 131-133ºC.

**^1^H NMR** (400 MHz, CDCl_3_) δ 8.42 (d, *J* = 5.5 Hz, 2H), 7.43 – 7.35 (m, 2H), 7.29 – 7.20 (m, 3H), 7.17 – 7.10 (m, 3H), 7.04 (dd, *J* = 5.4, 3.4 Hz, 2H), 6.99 (dd, *J* = 4.8, 1.4 Hz, 2H).

**^13^C NMR** (101 MHz, CDCl_3_) δ 170.7, 150.9, 150.6, 142.4, 135.3, 130.9, 129.7, 129.1, 128.5, 128.1, 127.5, 120.1.

**FTMS (ESI)** m/z: [M+H]^+^ calcd for C_18_H_15_N_2_O^+^ 275.1179; found 275.1179.


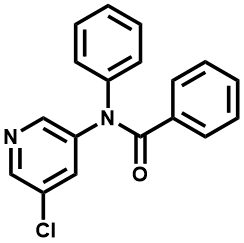
**N-(5-chloropyridin-3-yl)-N-phenylbenzamide (5k)**

According to the general procedure A, the product **5k** was obtained after silica gel chromatography (PE/EA: 4/1).

White solid; 32.6 mg, 53% yield; M.p. = 129-131ºC.

**^1^H NMR** (400 MHz, CDCl_3_) δ 8.36 (dd, *J* = 14.9, 2.2 Hz, 2H), 7.57 (t, *J* = 2.2 Hz, 1H), 7.44 (dd, *J* = 8.3, 1.2 Hz, 2H), 7.36 – 7.26 (m, 3H), 7.25 – 7.18 (m, 3H), 7.13 – 7.06 (m, 2H).

**^13^C NMR** (101 MHz, CDCl_3_) δ 170.6, 145.7, 145.7, 142.6, 140.9, 134.8, 133.4, 131.7, 130.9, 129.7, 129.2, 128.2, 128.0, 127.4.

**FTMS (ESI)** m/z: [M+H]^+^ calcd for C_18_H_14_ClN_2_O^+^ 309.0789; found 309.0796.


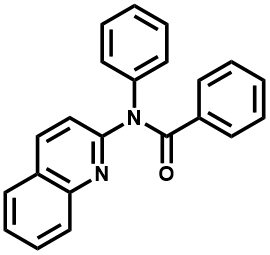
**N-phenyl-N-(quinolin-2-yl)benzamide (5l)**

According to the general procedure A, the product **5l** was obtained after silica gel chromatography (PE/EA: 1/1).

White solid; 33.1 mg, 51% yield; M.p. = 153-155ºC.

**^1^H NMR** (400 MHz, CDCl_3_) δ 8.08 (d, *J* = 8.7 Hz, 1H), 7.79 – 7.74 (m, 2H), 7.64 – 7.58 (m, 1H), 7.55 – 7.51 (m, 2H), 7.48 (dd, *J* = 11.4, 4.4 Hz, 1H), 7.38 – 7.28 (m, 4H), 7.26 – 7.16 (m, 5H).

**^13^C NMR** (101 MHz, CDCl_3_) δ 171.5, 155.4, 147.1, 142.6, 137.9, 136.2, 130.5, 129.8, 129.2, 129.1, 128.9, 128.0, 127.9, 127.3, 126.8, 126.5, 126.3, 119.8.

**FTMS (ESI)** m/z: [M+H]^+^ calcd for C_22_H_17_N_2_O^+^ 325.1335; found 325.1336.

# Mechanistic studies

## 6.1 Redical quenching experiments

To investigate the reaction pathway under light irradiation, we evaluated the effects of free radical inhibitors on the reaction process. Under the corresponding standard reaction conditions, TEMPO (2.0 equiv.) or BHT (2.0 equiv.) was introduced. The reactions were completely suppressed, and no radical trapping adducts were detected (see Table S5).


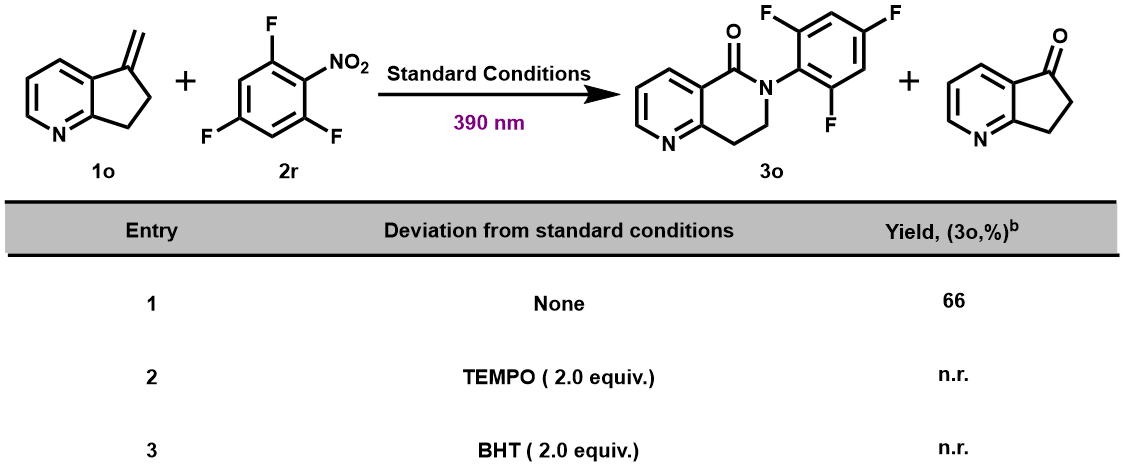
**Table S5:** Intermolecular radical quenching experiments^a^

^a^Reaction conditions: **1o** (0.3 mmol), **2r** (0.2 mmol), in MeCN (0.04 M), 390 nm irradiation (24W), under air atmosphere and room temperature for 48 h unless noted otherwise. ^b^Isolated yield. n.r. = no reaction.

## **6.2 Deuterium labeling experiment**


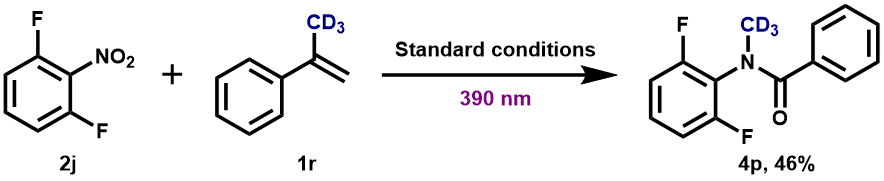
Under standard conditions, 1,3-difluoro-2-nitrobenzene (**2j**) and (prop-1-en-2-yl-3,3,3-d3)benzene (**1r**) react to form the deuterated product N-(2,6-difluorophenyl)-N-(methyl-d3)benzamide (**4p**, 46%).


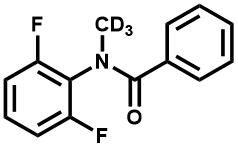
**N-(2,6-difluorophenyl)-N-(methyl-d3)benzamide (4p)**

**^1^H NMR** (400 MHz, CDCl_3_) δ 7.34 (d, *J* = 7.2 Hz, 2H), 7.28 – 7.22 (m, 1H), 7.17 (t, *J* = 7.4 Hz, 2H), 7.15 – 7.08 (m, 1H), 6.80 (t, *J* = 8.0 Hz, 2H).

**^13^C NMR** (101 MHz, CDCl_3_) δ 171.7, 158.2 (dd, J = 251.3, 4.2 Hz), 135.3, 130.1, 129.1 (t, J = 9.9 Hz), 127.7, 127.3, 122.1 (t, J = 16.0 Hz), 112.0 (dd, J = 18.6, 5.1 Hz), 35.8 (dd, J = 42.9, 22.4 Hz).

**^19^F NMR** (376 MHz, CDCl_3_) δ -118.8.

**FTMS (ESI)** m/z: [M+Na]^+^ calcd for C_14_H_8_D_3_FNNa_2_O^+^ 273.0889; found 273.0884.

## **6.3 The capture and conversion of potential intermediate**


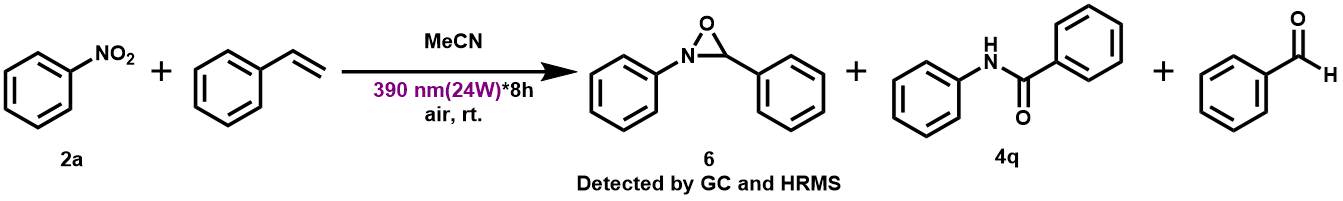

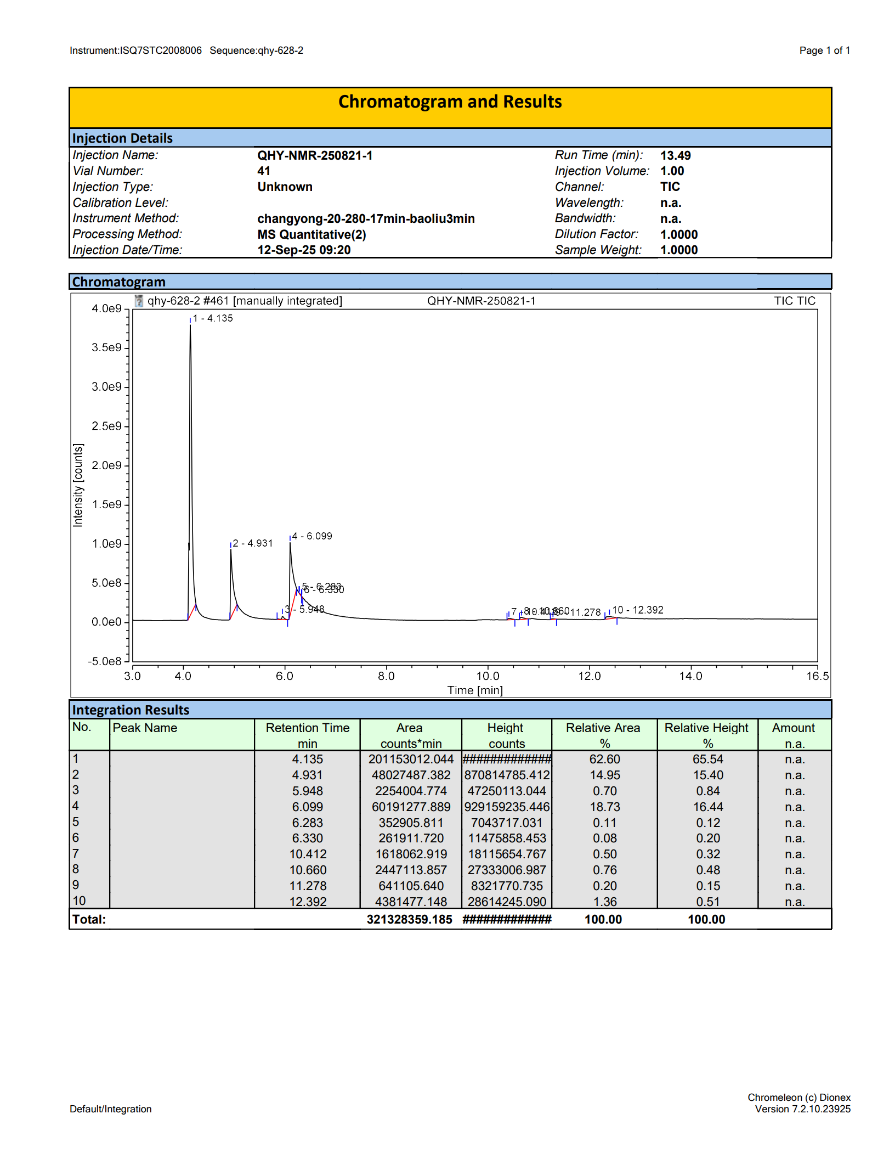
Under standard conditions, nitrobenzene (**2a**) and styrene react for 8 hours. Then the reaction solution was used for the detection by GC and HRMS. The result is that potential intermediate 2,3-diphenyl-1,2-oxaziridine (6) can be detected by GC and HRMS.

4q

6


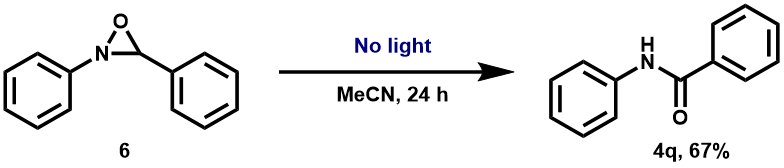


2,3-diphenyl-1,2-oxaziridine (**6**) was dissolved in acetonitrile and the solution was stirred for 24 hours under dark conditions to afford N-phenylbenzamide (**4q**, 67%).


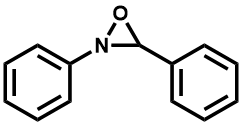
**2,3-diphenyl-1,2-oxaziridine (6)**

**^1^H NMR** (400 MHz, CDCl_3_) δ 8.39 (dd, *J* = 6.7, 3.0 Hz, 1H), 7.92 (s, 1H), 7.77 (dd, *J* = 7.7, 1.9 Hz, 1H), 7.53 – 7.39 (m, 3H).

**^13^C NMR** (101 MHz, CDCl_3_) δ 149.1, 134.8, 131.0, 130.7, 130.0, 129.2, 129.1, 128.7, 121.8.

**FTMS (ESI)** m/z: [M+Na]^+^ calcd for C_13_H_11_NNaO^+^ 220.0733; found 220.0733.


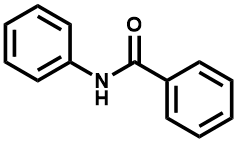
**N-phenylbenzamide (4q)**

**^1^H NMR** (400 MHz, CDCl_3_) δ 7.90 (s, 1H), 7.89 – 7.84 (m, 2H), 7.65 (d, *J* = 7.7 Hz, 2H), 7.54 (ddd, *J* = 6.4, 3.7, 1.2 Hz, 1H), 7.51 – 7.44 (m, 2H), 7.37 (t, *J* = 7.9 Hz, 2H), 7.15 (t, *J* = 7.4 Hz, 1H).

**^13^C NMR** (101 MHz, CDCl_3_) δ 165.8, 138.0, 135.0, 131.8, 129.1, 128.8, 127.0, 124.6, 120.2.

## 6.4 Cross-over study


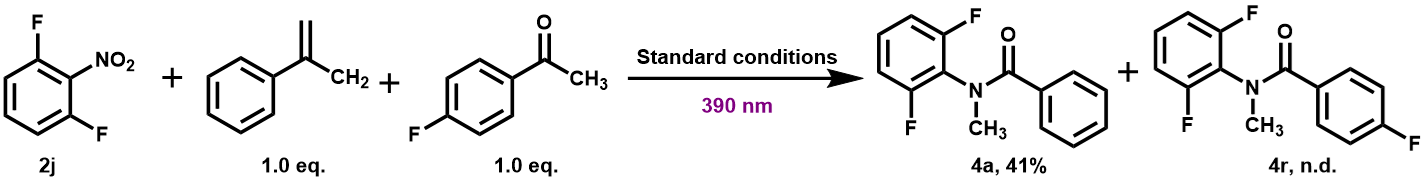
Under standard conditions, 1,3-difluoro-2-nitrobenzene (**2j**) was reacted with 2-phenylallylium and 1-(4-fluorophenyl)ethan-1-one. The result was that 3-((2,6-difluorophenyl)(methyl)carbamoyl)benzene-1-ylium (**4a**) was obtained with a yield of 41%, but N-(2,6-difluorophenyl)-4-fluoro-N-methylbenzamide (**4r**) was not detected in this study.

## **6.5 DFT calculations of energy barriers for two pathways involving the N-O=C dipole**


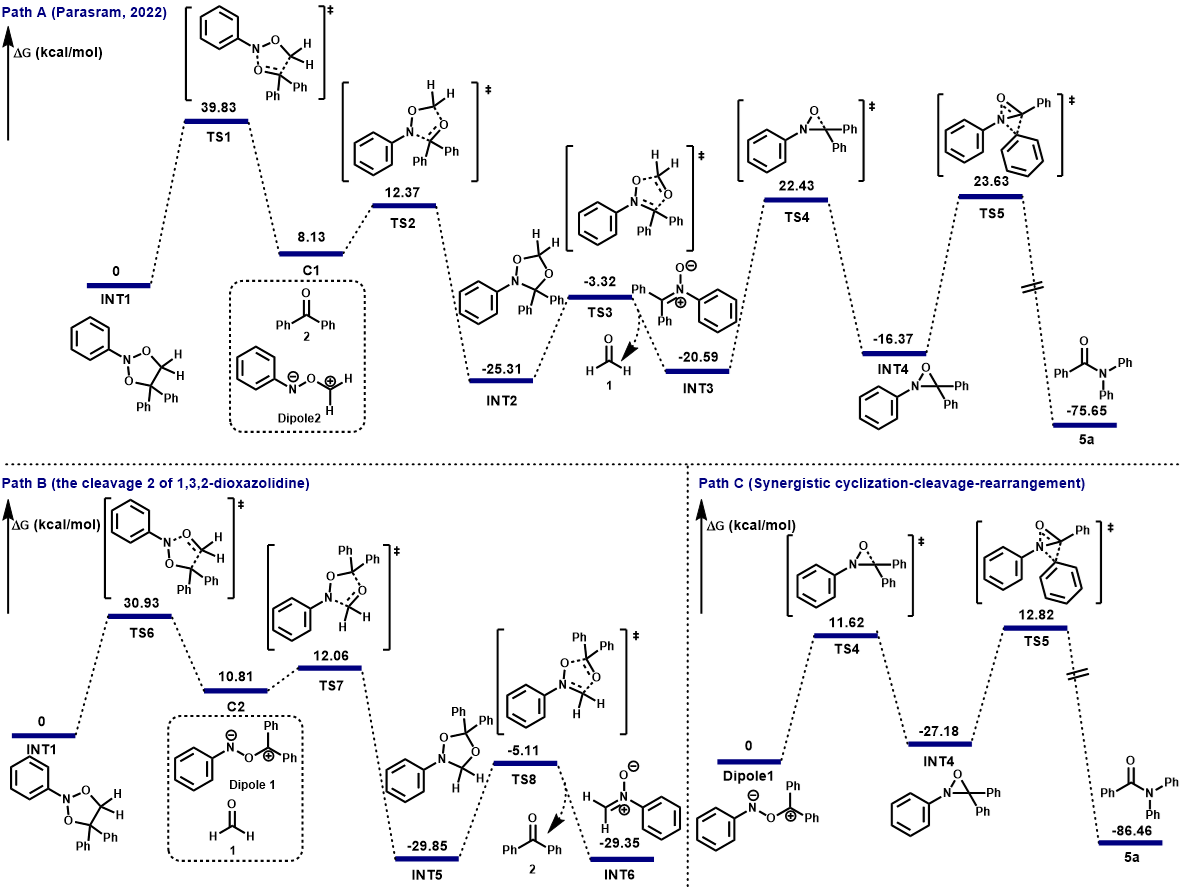
All density functional theory (DFT) calculations of both ground-state and transition-state structures were performed using M06-2X/6-31+G (d,p) with SMD = Ethyl acetate solvation and the Gaussian 09 software package. 1a-1d Vibrational frequency were also calculated at the same level to confirm that each stationary points found were either minima or transition states and to evaluate thermal corrections at standard states, including zero-point energy corrections. Based on the optimized structure, the single point energies (ESPE) were calculated at M06-2X/6-31+G(d,p) level in gas phase while applying Boys and Bernard’s counterpoise (CP) technique to analyze the effect of the basis set superposition error (BSSE) for the complex at the same level with single point energies used. 1e Binding energies (EBE) is defined as EBE = ESPE(AB) - ESPE(A) - ESPE(B) + EBSSE. 1f The topological analysis was calculated by Multiwfn 3.8 package 1g using the wave function derived from Gaussian.

## **6.6 Migration selectivity supported by** **DFT calculations**

DFT calculations provide theoretical support for the priority of alkyl migration over aryl migration. Considering the conversion of unsymmetrical alkyl/aryl alkenes, unsymmetrical dialkyl alkenes, and unsymmetrical di-aryl alkenes in this reaction. The migration selectivity (Kinetic support) of this system is as follows:

a) Alkyl group > Aryl group

b) Electron-deficient aryl > Electron-donating aryl


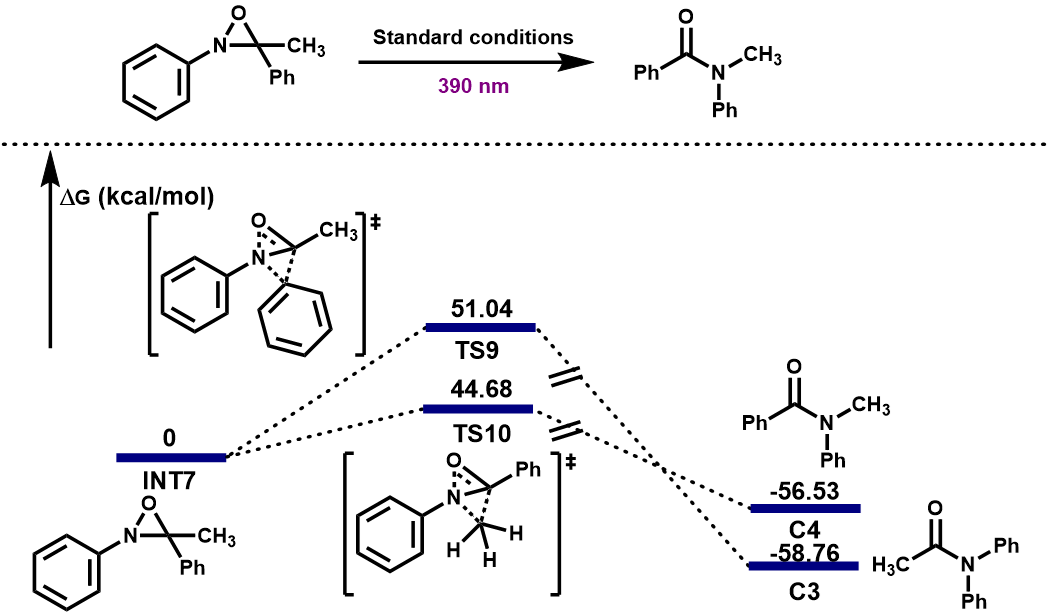

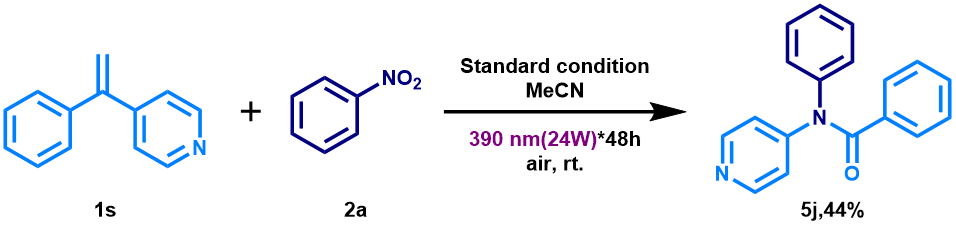
c) -CH3 > -CH2R, -CH3 > -CHR1R2

Structure: INT 1

Charge = 0 Multiplicity = 1

Number of imaginary frequencies: 0

Free Energy: -977.053837 hartree

C 0.20092374 1.49571596 -0.07958190

C 0.45753620 2.68961419 -0.76709848

C -1.07574152 0.91977565 -0.12796087

C -0.56251654 3.30757197 -1.50299422

H 1.43229881 3.12935671 -0.73016112

C -2.09579461 1.53773412 -0.86385555

H -1.27167075 0.00820771 0.39697306

C -1.83918150 2.73163105 -1.55137412

H -2.61801391 3.20345458 -2.11324834

H -0.36658656 4.21913842 -2.02793045

H -3.07055681 1.09799079 -0.90079415

C 3.22356640 0.10053589 0.85456953

C 2.37572834 -1.06445706 0.49369486

H 2.59433710 -1.95771327 1.04068989

H 2.49508792 -1.27077117 -0.54941960

O 1.07092277 -0.54843293 0.73265849

O 2.52716275 1.11271756 0.15695872

N 1.27090934 0.84750812 0.69233612

C 3.20368242 0.37161894 2.37039229

C 3.26225683 1.68795316 2.84762935

C 3.12701375 -0.69802972 3.27255393

C 3.24416098 1.93463881 4.22702802

H 3.32079499 2.50465365 2.15880892

C 3.10891650 -0.45134399 4.65195257

H 3.08229426 -1.70308028 2.90817292

C 3.16749108 0.86499022 5.12918964

H 3.28888401 2.93968919 4.59140911

H 3.05037808 -1.26804446 5.34077300

H 3.15367515 1.05334016 6.18239115

C 4.69374343 -0.06508924 0.42707938

C 5.28892164 -1.33374359 0.44125600

C 5.43642636 1.05284629 0.02388689

C 6.62678333 -1.48446215 0.05224187

H 4.72186663 -2.18731223 0.74910204

C 6.77428801 0.90212771 -0.36512736

H 4.98199441 2.02149195 0.01306213

C 7.36946580 -0.36652686 -0.35095209

H 7.08121546 -2.45310773 0.06306720

H 7.34134376 1.75569673 -0.67297099

H 8.39095185 -0.48160435 -0.64797639

Structure: TS 1

Charge = 0 Multiplicity = 1

Number of imaginary frequencies: 0

Free Energy: -976.990361 hartree

C 2.67636119 -1.29790911 0.13123240

C 3.16307719 -0.57021511 -0.96200360

C 3.52390719 -1.62448911 1.19422740

C 4.49095519 -0.14684311 -0.97000060

H 2.49884419 -0.33989611 -1.78686160

C 4.85120119 -1.19394411 1.17191840

H 3.13958119 -2.20499911 2.02436540

C 5.34103319 -0.45257511 0.09583540

H 6.37538619 -0.12107411 0.08408940

H 4.86179119 0.42435089 -1.81688160

H 5.50344219 -1.44192611 2.00508340

C -0.67065400 -0.11179700 0.22014000

C 0.32434419 -0.80527311 1.81333640

H -0.29944581 -0.97169611 2.68992940

H 1.14186919 -0.11801611 2.06104440

O 0.82599119 -2.07131111 1.37968640

O 0.21168000 -0.59961300 -0.83393400

N 1.33275419 -1.82774111 0.07599440

C -2.04071600 -0.74888800 -0.01732000

C -2.69530800 -1.56827100 0.90485800

C -2.65835400 -0.49893700 -1.25225900

C -3.94529900 -2.11930300 0.60580900

H -2.23877600 -1.80537100 1.85968400

C -3.90100700 -1.04785000 -1.55381300

H -2.14930200 0.12931300 -1.97718400

C -4.55276200 -1.85998800 -0.62086400

H -4.43781100 -2.75600400 1.33588200

H -4.36223700 -0.84306100 -2.51625800

H -5.52409400 -2.28874600 -0.85224000

C -0.71236800 1.41397800 0.18573100

C -1.84117900 2.10039300 0.65086900

C 0.40649000 2.14734900 -0.22926400

C -1.85182300 3.49525200 0.69859000

H -2.71619600 1.54260600 0.96955200

C 0.39155900 3.54126800 -0.18646000

H 1.28396100 1.62216600 -0.58925600

C -0.73688700 4.22081800 0.27708500

H -2.73598000 4.01290800 1.06100500

H 1.26454700 4.09685300 -0.51889400

H -0.74764200 5.30706600 0.30753800

Structure: Dipole 2

Charge = 0 Multiplicity = 1

Number of imaginary frequencies: 0

Free Energy: -977.040888 hartree

C -0.04379930 1.24504418 -0.13483930

C 0.41331715 2.24158796 -1.00768924

C -1.41867584 1.07685942 0.07811692

C -0.50444282 3.06994889 -1.66758070

H 1.46306595 2.37000168 -1.17028495

C -2.33643588 1.90521917 -0.58177594

H -1.76769438 0.31597636 0.74455748

C -1.87931939 2.90176364 -1.45462507

H -2.58004961 3.53423370 -1.95846907

H -0.15542425 3.83083248 -2.33402066

H -3.38618461 1.77680673 -0.41917873

C 3.74320498 2.07785453 3.24547220

C 2.65254498 -1.09366935 0.39882559

H 2.81749393 -1.67954893 1.27884659

H 3.48549294 -0.73543818 -0.16930126

O 1.31890705 -0.78942901 -0.01800493

O 2.51047593 1.94670851 3.02925385

N 0.91888611 0.37613611 0.55735689

C 4.28183887 3.38033469 3.86597118

C 4.58855814 4.47597857 3.04503194

C 4.29964145 3.54038834 5.25971412

C 4.98219778 5.69607885 3.61100709

H 4.52270077 4.37916456 1.98145785

C 4.69310490 4.75991807 5.82703984

H 4.01246334 2.72687809 5.89266736

C 5.04189163 5.83595913 5.00250003

H 5.23617450 6.52143904 2.97920301

H 4.72586443 4.86891734 6.89096934

H 5.35142771 6.76437419 5.43508860

C 4.71315559 0.93586776 2.88957612

C 5.08069362 -0.00396594 3.86429755

C 5.36961033 0.93162429 1.64961538

C 6.03960335 -0.98442711 3.57600474

H 4.62573139 0.02732771 4.83224904

C 6.32869625 -0.04826634 1.35997199

H 5.13596884 1.67961418 0.92103953

C 6.65842508 -1.01138926 2.32072981

H 6.29964183 -1.71207933 4.31614138

H 6.80995191 -0.05955764 0.40437506

H 7.38376596 -1.76546731 2.09679476

Structure: 2

Charge = 0 Multiplicity = 1

Number of imaginary frequencies: 0

Free Energy: -576.399774 hartree

C 3.97021700 6.13283646 -4.91633116

O 4.79255211 6.26233055 -5.86002964

C 2.80836152 5.13804161 -5.09542619

C 1.66960017 5.51387325 -5.82061247

C 2.88983437 3.85694666 -4.53321640

C 0.61231241 4.60860934 -5.98359042

H 1.60739359 6.49201788 -6.24987162

C 1.83254624 2.95168305 -4.69619361

H 3.75930287 3.56999172 -3.97951847

C 0.69378547 3.32751422 -5.42138103

H -0.25715690 4.89556494 -6.53728673

H 1.89475260 1.97353860 -4.26693400

H -0.11347724 2.63632517 -5.54581821

C 3.91722438 7.23443993 -3.84150020

C 4.58122477 7.05466217 -2.62055573

C 3.20500070 8.41667683 -4.08434850

C 4.53300128 8.05712122 -1.64245946

H 5.12502602 6.15199941 -2.43513692

C 3.15677704 9.41913579 -3.10625214

H 2.69802160 8.55394110 -5.01656666

C 3.82077742 9.23935803 -1.88530767

H 5.03998343 7.91985849 -0.71024272

H 2.61297756 10.32179945 -3.29167179

H 3.78395878 10.00475830 -1.13850996

Structure: TS 2

Charge = 0 Multiplicity = 1

Number of imaginary frequencies: 0

Free Energy: -977.034117 hartree

C 0.71693489 -1.06180018 0.80334779

C -0.00917211 -2.12328218 0.21043179

C 2.11237789 -0.99770918 0.59673379

C 0.64074389 -3.07076818 -0.56938321

H -1.08326511 -2.16371118 0.36556979

C 2.74763189 -1.96489918 -0.17784121

H 2.68181989 -0.18504918 1.03211079

C 2.02554389 -3.00386918 -0.76998521

H 2.53107989 -3.75188418 -1.37398321

H 0.06391389 -3.87335518 -1.02241021

H 3.82275989 -1.89855418 -0.32703721

C -0.86313067 1.47840350 0.22281884

C 0.09082789 1.80118782 2.58154479

H -0.85360511 1.55846782 3.05121179

H 0.67095089 2.67452082 2.85017079

O 0.75238789 0.88441682 1.97456779

O -1.03864316 2.29568789 1.17007508

N -0.06399711 -0.15248818 1.48573779

C -2.01659382 0.64274980 -0.21707029

C -2.26219687 0.36533940 -1.57014649

C -2.93223360 0.18766249 0.74632196

C -3.38676869 -0.37049557 -1.94915964

H -1.58666052 0.74370852 -2.33022092

C -4.04354816 -0.55703004 0.36883303

H -2.73571740 0.40566076 1.78917634

C -4.27496787 -0.84037206 -0.98255862

H -3.56744580 -0.57179337 -3.00162270

H -4.73350721 -0.91946849 1.12628747

H -5.14602677 -1.41985808 -1.27675284

C 0.40930169 1.51873504 -0.54041888

C 1.22268635 2.65942525 -0.40843856

C 0.86659545 0.44968239 -1.33223851

C 2.44239532 2.74228973 -1.07151241

H 0.86731129 3.47842781 0.20828623

C 2.09301846 0.53295361 -1.98953567

H 0.28439576 -0.46159653 -1.39880781

C 2.87990230 1.67880772 -1.86835688

H 3.05376190 3.63534306 -0.97154577

H 2.44040823 -0.31064910 -2.57874315

H 3.83450677 1.74173727 -2.38418379

Structure: Int 2

Charge = 0 Multiplicity = 1

Number of imaginary frequencies: 0

Free Energy: -977.094176 hartree

C -0.22296665 1.33633391 2.59737189

C -0.21513004 2.60038518 1.99236368

C -1.29768746 0.46310202 2.38205072

C -1.28201267 2.99120329 1.17203166

H 0.60544334 3.26711696 2.15676586

C -2.36456979 0.85391988 1.56171818

H -1.30367219 -0.50202812 2.84399010

C -2.35673204 2.11797023 0.95670807

H -3.17132064 2.41636856 0.33036643

H -1.27602790 3.95633339 0.71009221

H -3.18514196 0.18718712 1.39731397

C 2.16919663 1.57339380 3.04391648

C 2.46448883 -0.51448484 3.49812039

H 2.78021606 -1.34021149 4.10094027

H 2.88435096 -0.62227300 2.51985789

O 1.06133401 -0.43147558 3.41762624

O 2.85025797 0.73712595 4.01136817

N 0.89614265 0.92638350 3.45785723

C 2.63843459 2.86069150 3.74696724

C 2.45395026 4.10431203 3.12785861

C 3.24992545 2.78851189 5.00585206

C 2.88095979 5.27575265 3.76763333

H 1.98706308 4.15942280 2.16667228

C 3.67693268 3.95995273 5.64562791

H 3.39078220 1.83898013 5.47855578

C 3.49245079 5.20357302 5.02651809

H 2.74010490 6.22528422 3.29492869

H 4.14381878 3.90484206 6.60681477

H 3.81848091 6.09799404 5.51500084

C 3.05263092 0.92604960 1.96128573

C 4.15154587 0.13924318 2.33174974

C 2.75764119 1.12377279 0.60562775

C 4.95547267 -0.44983782 1.34655573

H 4.37677672 -0.01172335 3.36682469

C 3.56156574 0.53468864 -0.37956621

H 1.91859559 1.72451743 0.32277026

C 4.66048186 -0.25211613 -0.00910223

H 5.79452045 -1.05057943 1.62941319

H 3.33633380 0.68565366 -1.41464116

H 5.27429645 -0.70189445 -0.76131969

Structure: TS 3

Charge = 0 Multiplicity = 1

Number of imaginary frequencies: 0

Free Energy: -977.059124 hartree

C -1.65206497 0.90743422 -0.86137880

C -2.25774197 1.44516622 -1.99987080

C -2.44500997 0.32412922 0.13769120

C -3.64425197 1.40651922 -2.14588480

H -1.62220497 1.89069822 -2.75867780

C -3.83039997 0.28066822 -0.01615880

H -1.98049397 -0.12035578 1.00983720

C -4.43366897 0.82194922 -1.15372480

H -5.51367097 0.78349922 -1.26747880

H -4.10588697 1.82703822 -3.03511080

H -4.43931497 -0.18194378 0.75587620

C 0.60592803 0.03189522 -0.10041080

C 0.06212100 1.92931500 1.85049500

H 0.75462600 2.56946200 2.40827300

H -0.94515000 1.97286500 2.28933400

O 0.07159789 2.17184647 -0.06420599

O 0.51448700 0.59133000 1.82049500

N -0.21044897 1.04917722 -0.80721580

C 2.07363303 0.11836622 -0.57665480

C 2.67595303 -0.92605178 -1.28970980

C 2.85159603 1.25121422 -0.28699380

C 4.00393503 -0.83812478 -1.71458580

H 2.11697503 -1.82594378 -1.51466480

C 4.17511703 1.34054722 -0.71226180

H 2.42248603 2.08043422 0.25772720

C 4.76078003 0.29569322 -1.42986680

H 4.44312403 -1.66639178 -2.26455880

H 4.75173303 2.23154822 -0.47742880

H 5.79482103 0.36495622 -1.75718180

C 0.04086803 -1.37250878 -0.24839180

C 0.01982403 -2.23780878 0.84826920

C -0.41598597 -1.82878678 -1.49203680

C -0.46989197 -3.53787278 0.70835920

H 0.37823103 -1.88499578 1.80861520

C -0.90580497 -3.12632478 -1.62954680

H -0.39336197 -1.16261078 -2.34877680

C -0.93618497 -3.98519678 -0.52829380

H -0.48563997 -4.20075878 1.56939520

H -1.26615797 -3.46469278 -2.59715080

H -1.31951897 -4.99638578 -0.63513380

Structure: Int 3

Charge = 0 Multiplicity = 1

Number of imaginary frequencies: 0

Free Energy: -862.600283 hartree

C 0.22075383 1.96879077 1.07630769

C -0.05443544 2.94183704 2.04655188

C -0.69374238 1.73351933 0.04080492

C -1.24411992 3.67961341 1.98129204

H 0.64380225 3.12147131 2.83718178

C -1.88342811 2.47129380 -0.02445337

H -0.48362893 0.99057740 -0.69999845

C -2.15861714 3.44434045 0.94579051

H -3.06696789 4.00764870 0.89596348

H -1.45423243 4.42255675 2.72209426

H -2.58166566 2.29165975 -0.81508346

C 2.31282414 1.41207188 2.10061178

O 1.73573306 0.25059699 0.20318222

N 1.46867375 1.19489818 1.14476312

C 2.21387235 2.50144333 3.18220564

C 2.65104923 3.75728051 2.73985814

C 1.58502430 2.37740093 4.42843419

C 2.45937430 4.88907594 3.54373734

H 3.13119060 3.85198929 1.78833593

C 1.39335234 3.50919583 5.23231483

H 1.25123088 1.41854122 4.76617672

C 1.83052640 4.76503351 4.78996596

H 2.79316595 5.84793596 3.20599394

H 0.91321384 3.41448654 6.18383844

H 1.68417923 5.62918429 5.40374599

C 3.42693397 0.47268131 2.20465809

C 4.53120373 1.21797981 1.76987032

C 3.60494977 -0.83718425 2.66993708

C 5.81348927 0.65341265 1.80036132

H 4.39528499 2.21809195 1.41462140

C 4.88723540 -1.40175115 2.70042877

H 2.76181509 -1.40623675 3.00190571

C 5.99150510 -0.65645282 2.26564055

H 6.65662418 1.22246576 1.46839433

H 5.02315472 -2.40186176 3.05568178

H 6.97055899 -1.08751123 2.28892417

Structure: TS 4

Charge = 0 Multiplicity = 1

Number of imaginary frequencies: 0

Free Energy: -862.531724 hartree

C 1.78775000 -1.22368700 0.08988600

C 2.17708700 -1.31770500 -1.25265100

C 2.74738400 -1.16730900 1.10468300

C 3.53207800 -1.31338000 -1.57612800

H 1.41678100 -1.38143000 -2.02575500

C 4.10096200 -1.17303800 0.76638700

H 2.42150000 -1.09819400 2.13612100

C 4.50034300 -1.23949400 -0.57025100

H 5.55579100 -1.24002100 -0.82680200

H 3.83231600 -1.37032800 -2.61896000

H 4.84689800 -1.12189000 1.55512900

C -0.51358200 -0.19009300 0.46481800

O 0.02208260 -0.63344788 2.10588253

N 0.39755200 -1.32536200 0.37036300

C -0.17892500 1.26526900 0.21828600

C 1.11906000 1.75652700 0.00590200

C -1.23528900 2.19158800 0.27802000

C 1.34299300 3.12266800 -0.16339400

H 1.96495000 1.08752700 -0.02602300

C -1.00678000 3.55767200 0.12034400

H -2.24602000 1.84203700 0.45539700

C 0.28500600 4.03031900 -0.10899400

H 2.35631700 3.47517900 -0.33584500

H -1.84268400 4.24962100 0.17672400

H 0.46627900 5.09386200 -0.23915200

C -1.91891600 -0.61839300 0.11081200

C -2.70609700 -1.29792900 1.04252700

C -2.41758600 -0.38171900 -1.17619000

C -3.98801200 -1.72758500 0.69432600

H -2.30255000 -1.49197100 2.03033800

C -3.69320500 -0.82151900 -1.52605600

H -1.80750200 0.15368700 -1.89885200

C -4.48363000 -1.49244300 -0.58901700

H -4.59772400 -2.25020400 1.42652600

H -4.07124400 -0.63831300 -2.52828100

H -5.48031300 -1.83054100 -0.85977700

Structure: Int 4

Charge = 0 Multiplicity = 1

Number of imaginary frequencies: 0

Free Energy: -862.593558 hartree

C 0.08507742 1.83783055 1.06742021

C -0.18241063 2.58965634 2.21943153

C -0.90113271 1.68942049 0.08289699

C -1.43610939 3.19307066 2.38692041

H 0.57058241 2.70297001 2.97113726

C -2.15483047 2.29283730 0.25038448

H -0.69689899 1.11538642 -0.79669012

C -2.42231915 3.04466153 1.40239667

H -3.37954569 3.50538247 1.53027775

H -1.64034225 3.76710684 3.26650634

H -2.90782367 2.17952322 -0.50132101

C 2.52290473 1.71648498 1.52449928

O 2.42150741 1.96127164 0.10083221

N 1.40014546 1.20487700 0.89173339

C 2.37696934 2.82014473 2.58856602

C 2.78303359 4.12997213 2.29979971

C 1.83810390 2.51464770 3.84563307

C 2.65023487 5.13430193 3.26810137

H 3.19446972 4.36322579 1.34000127

C 1.70530353 3.51897788 4.81393411

H 1.52806124 1.51456623 4.06611121

C 2.11136861 4.82880508 4.52516811

H 2.96027557 6.13438384 3.04762249

H 1.29386630 3.28572446 5.77373213

H 2.00997193 5.59563361 5.26448721

C 3.63931802 0.75976119 1.98264876

C 4.97501657 1.18357892 1.96816202

C 3.31955556 -0.53467521 2.41405154

C 5.99095253 0.31296071 2.38507929

H 5.21916235 2.17190968 1.63877598

C 4.33549163 -1.40529380 2.83096774

H 2.29972001 -0.85826922 2.42511314

C 5.67119026 -0.98147633 2.81648024

H 7.01078810 0.63655469 2.37401759

H 4.09134565 -2.39362386 3.16035571

H 6.44688000 -1.64621297 3.13480496

Structure: TS 5

Charge = 0 Multiplicity = 1

Number of imaginary frequencies: 0

Free Energy: -862.529819 hartree

C -0.05872900 1.58567600 -0.36594500

C -0.94457300 2.39819300 0.34820500

C 1.25755200 1.98409900 -0.61646200

C -0.48971300 3.62099700 0.83972100

H -1.96135600 2.06430800 0.51996400

C 1.69021000 3.21396800 -0.12460200

H 1.91553200 1.34700100 -1.19201500

C 0.82449900 4.03144800 0.60664100

H 1.17133800 4.98804300 0.98667000

H -1.17072400 4.25670500 1.39801700

H 2.70994300 3.53420000 -0.31797000

C 0.06218200 -1.04608400 -0.83554800

O 0.19288144 -0.37082996 -2.62555900

N -0.55780500 0.33433900 -0.83410700

C 1.37449900 -1.31006500 -0.24185100

C 2.53486900 -1.28867900 -1.03746600

C 1.47560400 -1.71046600 1.10357600

C 3.76930400 -1.63657100 -0.49171200

H 2.45593500 -1.00349700 -2.08228100

C 2.71504500 -2.04138300 1.64387500

H 0.58093800 -1.77048600 1.71358600

C 3.86704500 -2.00486100 0.85185700

H 4.65788700 -1.61577700 -1.11731200

H 2.78222700 -2.33758900 2.68729000

H 4.83094200 -2.26960100 1.27742700

C -1.66166300 -0.66333300 -0.33055700

C -1.91272400 -0.63604900 1.06518000

C -2.72726600 -0.98074900 -1.21554300

C -3.16031100 -1.03238100 1.54676800

H -1.13107100 -0.31957300 1.74772800

C -3.94677000 -1.38025000 -0.69657600

H -2.54762700 -0.94654700 -2.28443900

C -4.18742200 -1.41875200 0.68705900

H -3.32132200 -1.03104800 2.62257800

H -4.73933800 -1.65455000 -1.38952800

H -5.15239100 -1.72849700 1.07468100

Structure: 5a

Charge = 0 Multiplicity = 1

Number of imaginary frequencies: 0

Free Energy: -862.688029 hartree

C 0.89830534 1.60427859 1.33677436

C 0.70486351 1.34431019 2.70019603

C -0.19819922 1.90944179 0.51917108

C -0.58508266 1.38950662 3.24601475

H 1.54206886 1.11130954 3.32445392

C -1.48814525 1.95463941 1.06499005

H -0.05050225 2.10793210 -0.52183190

C -1.68158693 1.69467216 2.42841197

H -2.66648905 1.72918394 2.84515737

H -0.73277980 1.19101482 4.28701744

H -2.32535034 2.18764208 0.44073260

C 2.86826279 2.88817223 0.85377867

O 3.71098139 3.25038904 -0.00772867

N 2.25139614 1.55687286 0.76423794

C 2.48320465 3.83959473 2.00187512

C 3.07128207 5.10876788 2.08723566

C 1.54472354 3.43621644 2.96128177

C 2.72087948 5.97456217 3.13200367

H 3.78783368 5.41675593 1.35470773

C 1.19431892 4.30201178 4.00604825

H 1.09571447 2.46717403 2.89610792

C 1.78239590 5.57118515 4.09140846

H 3.16988951 6.94360411 3.19717823

H 0.47776793 3.99402341 4.73857665

H 1.51485333 6.23223956 4.88911019

C 3.06605480 0.58807367 1.51167317

C 4.45034904 0.77539262 1.62375808

C 2.45840151 -0.52283287 2.11214473

C 5.22698995 -0.14819482 2.33631483

H 4.91430594 1.62359390 1.16528391

C 3.23504266 -1.44642123 2.82470001

H 1.40146224 -0.66585542 2.02656480

C 4.61933691 -1.25910231 2.93678489

H 6.28392930 -0.00517257 2.42189429

H 2.77108600 -2.29462344 3.28317271

H 5.21231939 -1.96428145 3.48083835

Structure: TS 6

Charge = 0 Multiplicity = 1

Number of imaginary frequencies: 0

Free Energy: -977.004548 hartree

C 2.19102575 1.10147184 0.71893387

C 2.64045275 0.08617384 1.56711187

C 3.04547575 1.64866384 -0.24486713

C 3.94120175 -0.39672416 1.42980787

H 1.96966675 -0.32479916 2.31011687

C 4.34288975 1.15434884 -0.37250513

H 2.68485775 2.44510384 -0.88574713

C 4.79732375 0.13002584 0.46086887

H 5.80900975 -0.25190316 0.35756287

H 4.28238475 -1.19490516 2.08349487

H 5.00021275 1.57472084 -1.12898813

C -0.66427625 0.04678884 0.26987287

C -0.25099619 1.04440548 -1.39936812

H -1.17496069 1.35970678 -1.88365977

H 0.44904466 0.62714382 -2.13070026

O 0.31761715 2.20469343 -0.77664282

O -0.05233425 0.74106184 1.41376887

N 0.88416075 1.69466284 0.91889687

C -2.17636125 0.23044784 0.41783887

C -2.66970425 1.48956684 0.79081987

C -3.08118325 -0.79925016 0.13974087

C -4.04242725 1.71034584 0.88132687

H -1.96904025 2.28683484 1.01209587

C -4.45727425 -0.57521316 0.23069387

H -2.71577925 -1.78083916 -0.14302213

C -4.94231125 0.67823284 0.60194487

H -4.41045325 2.68989784 1.17511187

H -5.14764425 -1.38655516 0.01533887

H -6.01274525 0.85059384 0.67688487

C -0.23949525 -1.41398016 0.26166987

C -0.04481425 -2.12072016 -0.92981913

C -0.10951325 -2.09500416 1.47927987

C 0.30241875 -3.47399416 -0.90621013

H -0.17062225 -1.62466816 -1.88844013

C 0.24168175 -3.44275216 1.50516487

H -0.27844525 -1.55448216 2.40508187

C 0.45269975 -4.13730616 0.31089887

H 0.45574675 -4.00565816 -1.84142813

H 0.34877375 -3.95298916 2.45868387

H 0.72737175 -5.18840916 0.33040187

Structure: Dipole 1

Charge = 0 Multiplicity = 1

Number of imaginary frequencies: 0

Free Energy: -977.036616 hartree

C 0.33877555 1.62639131 -0.02320964

C 0.56748017 2.90161678 -0.55745111

C -0.85446483 0.94998130 -0.31055966

C -0.39705345 3.50042969 -1.37904645

H 1.47854505 3.41807036 -0.33805429

C -1.81900005 1.54879634 -1.13215113

H -1.02908618 -0.02368089 0.09734617

C -1.59029319 2.82401931 -1.66639534

H -2.32673559 3.28122621 -2.29370027

H -0.22243227 4.47409191 -1.78695278

H -2.73006498 1.03234210 -1.35154982

C 3.50425907 0.29950820 1.09204238

C 1.03565482 -2.37914364 0.39638319

H 1.66825981 -2.15951943 1.23093456

H 1.22442443 -3.24369817 -0.20512499

O 0.06965823 -1.62065703 0.12230627

O 2.32253922 0.22073540 0.29064842

N 1.35052711 0.99826053 0.83859719

C 3.39155024 0.54600663 2.60800226

C 3.38998585 1.85565265 3.10674471

C 3.29055391 -0.53932489 3.48878324

C 3.28743127 2.07997045 4.48626806

H 3.46709607 2.68432702 2.43424845

C 3.18799257 -0.31500887 4.86830654

H 3.29174922 -1.53926918 3.10798315

C 3.18643502 0.99463675 5.36704898

H 3.28624246 3.07991408 4.86706973

H 3.11087207 -1.14368530 5.54080351

H 3.10812938 1.16590807 6.42034456

C 4.88959531 0.13783594 0.43912314

C 5.47019839 -1.13321598 0.33305993

C 5.56964636 1.26176396 -0.04897261

C 6.73085330 -1.28033844 -0.26109999

H 4.95096349 -1.99135967 0.70573194

C 6.83029837 1.11464050 -0.64313736

H 5.12634291 2.23223960 0.03201094

C 7.41090248 -0.15641093 -0.74920093

H 7.17415813 -2.25081392 -0.34208126

H 7.34953032 1.97278406 -1.01581229

H 8.37343985 -0.26874230 -1.20285594

Structure: 1

Charge = 0 Multiplicity = 1

Number of imaginary frequencies: 0

Free Energy: -114.486362 hartree

C 2.74424939 -0.47825110 3.51402366

H 2.67811953 -1.33349124 4.15362599

H 2.79722420 -0.60646251 2.45305453

O 2.75972070 0.67836146 4.00958253

Structure: TS 7

Charge = 0 Multiplicity = 1

Number of imaginary frequencies: 0

Free Energy: -977.03462 hartree

C -2.67993564 -0.16450260 -0.17369926

C -2.93336164 -0.57055460 -1.49254126

C -3.75123764 -0.00555960 0.72100374

C -4.24282464 -0.81506260 -1.90054226

H -2.10506464 -0.70019460 -2.17807326

C -5.05586264 -0.24676060 0.29319874

H -3.57160564 0.31936240 1.74140574

C -5.31218764 -0.65500860 -1.01649226

H -6.33006564 -0.84639060 -1.34325926

H -4.42585464 -1.13575860 -2.92279526

H -5.87514564 -0.11802060 0.99557774

C 0.65817130 -0.00543166 0.00040582

C -0.92879572 -0.22423953 2.10858144

H -1.19610572 0.60397847 2.77313444

H -1.38640772 -1.16187353 2.46273044

O 0.47396028 -0.37854553 2.03593144

O -0.53643370 -0.35103266 -0.69314718

N -1.37513664 0.15958140 0.24552274

C 1.09604230 1.42130834 -0.30708418

C 0.84661830 1.97879434 -1.56433918

C 1.80226030 2.15729234 0.64842182

C 1.29404230 3.26631634 -1.86110218

H 0.29040030 1.40344134 -2.29725118

C 2.25273830 3.44384034 0.34969082

H 1.98578330 1.71902534 1.62416082

C 1.99986830 4.00117634 -0.90557718

H 1.08873130 3.69687734 -2.83763418

H 2.79820130 4.01194334 1.09868082

H 2.34865030 5.00404634 -1.13742118

C 1.68435930 -1.07836866 -0.32471018

C 1.39871930 -2.39836866 0.04783282

C 2.87744030 -0.79387766 -0.99204718

C 2.29605930 -3.42112866 -0.24521218

H 0.47069730 -2.60931166 0.56845782

C 3.77865030 -1.82107166 -1.28482418

H 3.10538430 0.22588934 -1.28211418

C 3.49079030 -3.13362566 -0.91321618

H 2.06699030 -4.44227266 0.04753582

H 4.70562830 -1.59071866 -1.80286518

H 4.19294830 -3.93147266 -1.14050518

Structure: Int 5

Charge = 0 Multiplicity = 1

Number of imaginary frequencies: 0

Free Energy: -977.101402 hartree

C -2.72137957 0.11272376 0.03688444

C -3.08042226 -0.15185916 -1.28650611

C -3.72584147 0.31343473 0.98612084

C -4.39150239 -0.20898207 -1.62504556

H -2.32537942 -0.30845363 -2.02832042

C -5.02573542 0.24824941 0.60819268

H -3.47085681 0.51734026 2.00509353

C -5.35505348 -0.01064676 -0.68581235

H -6.38585849 -0.05893336 -0.96867176

H -4.67045034 -0.41146409 -2.63800611

H -5.79810870 0.40148408 1.33266398

C 0.70373548 -0.36696552 0.45359158

C -1.06487271 -0.29621920 1.77211180

H -1.47993579 0.33227486 2.53212355

H -1.49343100 -1.27259782 1.86112059

O 0.35743934 -0.39587132 1.85784652

O -0.49474522 -0.66551588 -0.27402192

N -1.30522689 0.17906163 0.42548561

C 1.13525718 1.06149721 0.07295628

C 0.88088581 1.55041775 -1.21552106

C 1.78231315 1.87247781 1.01505553

C 1.27357093 2.85031868 -1.56189935

H 0.38684130 0.93121721 -1.93483373

C 2.17500042 3.17237790 0.66867650

H 1.97653088 1.49917631 1.99883673

C 1.92062924 3.66129836 -0.61980091

H 1.07935223 3.22362056 -2.54568022

H 2.66904541 3.79157825 1.38798901

H 2.22045646 4.65380022 -0.88426961

C 1.85371624 -1.34120449 0.13735957

C 2.01292837 -2.50765377 0.89760264

C 2.74098642 -1.06131280 -0.91065486

C 3.05941346 -3.39420906 0.60983425

H 1.33547674 -2.72135824 1.69778367

C 3.78747053 -1.94786890 -1.19842429

H 2.61942351 -0.17070365 -1.49111829

C 3.94668566 -3.11431569 -0.43817802

H 3.18097674 -4.28481790 1.19029808

H 4.46492112 -1.73416528 -1.99860643

H 4.74570134 -3.79121941 -0.65789439

Structure: TS 8

Charge = 0 Multiplicity = 1

Number of imaginary frequencies: 0

Free Energy: -977.06198 hartree

C -3.10236081 -0.04825611 0.04039327

C -3.35578681 -0.45430811 -1.27844873

C -4.17366281 0.11068689 0.93509627

C -4.66524981 -0.69881611 -1.68644973

H -2.52748981 -0.58394811 -1.96398073

C -5.47828781 -0.13051411 0.50729127

H -3.99403081 0.43560889 1.95549827

C -5.73461281 -0.53876211 -0.80239973

H -6.75249081 -0.73014411 -1.12916673

H -4.84827981 -1.01951211 -2.70870273

H -6.29757081 -0.00177411 1.20967027

C 0.77610000 -0.10250300 0.40119000

C -1.45512367 -0.01205555 1.88531844

H -1.72243367 0.81616245 2.54987144

H -1.91273567 -0.94968955 2.23946744

O 0.39941900 -0.21605900 1.78927000

O -0.87029167 -0.39840655 -0.26896456

N -1.79756181 0.27582789 0.45961527

C 1.21397100 1.32423700 0.09370000

C 0.96454700 1.88172300 -1.16355500

C 1.92018900 2.06022100 1.04920600

C 1.41197100 3.16924500 -1.46031800

H 0.40832900 1.30637000 -1.89646700

C 2.37066700 3.34676900 0.75047500

H 2.10371200 1.62195400 2.02494500

C 2.11779700 3.90410500 -0.50479300

H 1.20666000 3.59980600 -2.43685000

H 2.91613000 3.91487200 1.49946500

H 2.46657900 4.90697500 -0.73663700

C 1.80228800 -1.17544000 0.07607400

C 1.51664800 -2.49544000 0.44861700

C 2.99536900 -0.89094900 -0.59126300

C 2.41398800 -3.51820000 0.15557200

H 0.58862600 -2.70638300 0.96924200

C 3.89657900 -1.91814300 -0.88404000

H 3.22331300 0.12881800 -0.88133000

C 3.60871900 -3.23069700 -0.51243200

H 2.18491900 -4.53934400 0.44832000

H 4.82355700 -1.68779000 -1.40208100

H 4.31087700 -4.02854400 -0.73972100

Structure: Int 6

Charge = 0 Multiplicity = 1

Number of imaginary frequencies: 0

Free Energy: -400.700837 hartree

C 0.85332146 3.20677513 -2.18254460

C 1.63466025 4.36034392 -2.33324531

C -0.41943534 3.14145893 -2.76542185

C 1.14324399 5.44859485 -3.06682688

H 2.60643779 4.41021479 -1.88820450

C -0.91085158 4.22970984 -3.49900346

H -1.01600626 2.26068489 -2.65035558

C -0.12951196 5.38327783 -3.64970591

H -0.50471828 6.21418071 -4.20981359

H 1.73981508 6.32936872 -3.18189350

H -1.88262877 4.17983864 -3.94404498

C 0.64755836 1.00041940 -1.27394738

H -0.32422030 0.95054960 -1.71898585

H 1.02276753 0.16951381 -0.71384562

O 2.60395049 2.12863956 -0.84739604

N 1.36879392 2.06525236 -1.41305522

Structure: Int 7

Charge = 0 Multiplicity = 1

Number of imaginary frequencies: 0

Free Energy: -670.93199 hartree

C -3.53875381 1.10992115 -0.43612447

C -2.16565757 1.22659974 -0.18134065

C -1.63142619 2.45473476 0.23121622

C -2.47029134 3.56619071 0.38899108

C -3.84338715 3.44951280 0.13420466

C -4.37761841 2.22137798 -0.27835296

H -3.94665141 0.17221267 -0.75112028

H -1.52516541 0.37797847 -0.30180527

H -2.06239414 4.50389853 0.70398935

H -4.48387918 4.29813428 0.25466852

H -5.42600735 2.13229212 -0.47288918

N -0.19111514 2.57712540 0.49847007

O 0.73738487 1.94547330 -0.28931969

C 0.51610854 1.52389179 1.14222358

C 1.97001601 1.66097935 1.63105899

H 2.02829526 1.37689985 2.66101160

H 2.60346113 1.02456421 1.04914571

H 2.28847369 2.67672313 1.52266489

C 0.02126262 0.09386916 0.85628162

C -0.73593036 -0.58610295 1.81062335

C 0.32993590 -0.52153028 -0.35679400

C -1.18373543 -1.88143174 1.55213607

H -0.97846159 -0.10094488 2.76720165

C -0.11876404 -1.81688140 -0.61591509

H 0.92656093 0.01431576 -1.10914806

C -0.87537830 -2.49692906 0.33834224

H -1.78003635 -2.41769074 2.30456415

H 0.12441707 -2.30169055 -1.57262678

H -1.22861618 -3.51823381 0.13473492

Structure: TS 9

Charge = 0 Multiplicity = 1

Number of imaginary frequencies: 0

Free Energy: -670.85065 hartree

C -3.01892663 -0.61644554 -0.94226574

C -2.08867063 0.42189046 -1.00958574

C -1.42348263 0.82367846 0.14969026

C -1.69190663 0.19960946 1.37314126

C -2.61731063 -0.84027354 1.42728826

C -3.28524063 -1.25290154 0.27092326

H -3.53488863 -0.92946554 -1.84618074

H -1.86635063 0.92021946 -1.94617174

H -1.17145763 0.52947646 2.26774126

H -2.81585763 -1.33067154 2.37652726

H -4.00795363 -2.06262254 0.31722126

N -0.52762063 1.93987146 0.16924526

O 0.24029403 2.09163299 -1.07867140

C 0.89594637 1.67679046 -0.02091674

C 1.80947437 2.71654046 0.58871126

H 2.05516237 2.47074546 1.62647626

H 2.74374837 2.76696946 0.01981926

H 1.31273137 3.68849746 0.56233326

C 0.11402405 0.05027252 -0.16404726

C 0.69554105 -0.53840548 0.96662174

C -0.01699595 -0.70383448 -1.33577526

C 1.13628405 -1.86084548 0.92615374

H 0.79549905 0.02933252 1.88727574

C 0.43091205 -2.02386048 -1.37722326

H -0.47456795 -0.24696848 -2.20559026

C 1.00743905 -2.60647348 -0.24729726

H 1.57942105 -2.30835948 1.81167374

H 0.32667205 -2.59832548 -2.29368026

H 1.35361305 -3.63600548 -0.27970826

Structure: TS 10

Charge = 0 Multiplicity = 1

Number of imaginary frequencies: 0

Free Energy: -670.860784 hartree

C -0.43672635 1.64565411 0.28132898

O -0.91886130 2.43422079 -0.57264937

N 0.89470104 1.57542545 0.53369472

C 1.81794217 0.47663398 0.21560178

C 3.08238225 0.75653727 -0.31585652

C 1.43181749 -0.85257696 0.44465938

C 3.95333451 -0.29563724 -0.61509482

H 3.38043731 1.76898808 -0.49194329

C 2.27788707 -1.87468994 0.15397313

H 0.46450317 -1.06253912 0.85100765

C 3.54933995 -1.61502134 -0.37926336

H 4.92224623 -0.09362959 -1.02166952

H 1.97267285 -2.88451682 0.33284171

H 4.21023854 -2.42548253 -0.60568274

C -1.22908362 0.34700387 0.04200179

C -2.23130963 0.30795032 -0.93674220

C -0.94790262 -0.79571433 0.80295795

C -2.95235687 -0.87382049 -1.15452776

H -2.44599718 1.18044085 -1.51774953

C -1.66894825 -1.97748583 0.58517078

H -0.18267912 -0.76589678 1.55024898

C -2.67117575 -2.01653875 -0.39357170

H -3.71757877 -0.90363872 -1.90182041

H -1.45425824 -2.84997741 1.16617560

H -3.22171015 -2.91884749 -0.55985698

C -0.10382242 1.99293763 2.11290786

H -0.32164935 1.18389339 2.77841431

H 0.56270967 2.50813829 2.77260725

H -0.92639939 2.62241430 2.38128493

Structure: C3

Charge = 0 Multiplicity = 1

Number of imaginary frequencies: 0

Free Energy: -671.025626 hartree

C -3.24446749 2.11346628 -0.97318661

C -2.14246421 1.46526472 -0.39930030

C -1.64471468 1.89288702 0.83896726

C -2.24896977 2.96870947 1.50334953

C -3.35097366 3.61691038 0.92946369

C -3.84872199 3.18928933 -0.30880478

H -3.62451025 1.78696722 -1.91863140

H -1.68110213 0.64385031 -0.90657084

H -1.86892748 3.29520803 2.44879468

H -3.81233541 4.43832513 1.43673397

H -4.69012576 3.68420579 -0.74697985

N -0.48876577 1.21295678 1.44094483

O 0.75127090 3.09345101 0.71957874

C 0.75057199 1.86549306 0.99469567

C 2.04807053 1.04635674 0.86387811

H 1.80702902 0.03328131 0.61795706

H 2.65810017 1.46549398 0.09116354

H 2.58059112 1.07115476 1.79162100

C -0.47779531 -0.19670024 1.02423540

C -1.14685842 -1.15952085 1.79186356

C 0.20172632 -0.57775265 -0.14065574

C -1.13639693 -2.50339441 1.39460249

H -1.66568881 -0.86857877 2.68128345

C 0.21218742 -1.92162615 -0.53791706

H 0.71257087 0.15738224 -0.72675687

C -0.45687336 -2.88444718 0.22971259

H -1.64723808 -3.23852993 1.98070580

H 0.73101844 -2.21256834 -1.42733655

H -0.44888313 -3.91052495 -0.07360334

Structure: C4

Charge = 0 Multiplicity = 1

Number of imaginary frequencies: 0

Free Energy: -671.022081 hartree

C 1.06248532 2.44467197 -1.21502631

C 0.91822265 1.70305074 -0.03472627

C 0.25432882 2.26051805 1.06636280

C -0.26529848 3.55960823 0.98715332

C -0.12103507 4.30122977 -0.19314644

C 0.54285513 3.74376092 -1.29423692

H 1.56938313 2.01903336 -2.05573220

H 1.31497264 0.71116784 0.02575310

H -0.77219278 3.98524832 1.82786057

H -0.51778132 5.29311426 -0.25362435

H 0.65300109 4.31000442 -2.19542289

N 0.64085617 0.08286519 2.44648041

O -0.50035279 1.94613187 3.35212822

C 0.09579463 1.44554807 2.36339435

C -0.17786412 -0.71696293 3.36890767

H -1.18473135 -0.75688921 3.00901286

H 0.21888136 -1.70884775 3.42938529

H -0.16368163 -0.26733967 4.33975152

C 0.62137181 -0.53484154 1.11270429

C -0.50119903 -1.26139207 0.69332261

C 1.72536756 -0.39717143 0.26055273

C -0.51977333 -1.85027413 -0.57820988

H -1.34412452 -1.36650576 1.34395901

C 1.70679335 -0.98605367 -1.01097968

H 2.58247520 0.15756627 0.58075984

C 0.58422388 -1.71260710 -1.43036004

H -1.37688115 -2.40501145 -0.89841716

H 2.54971870 -0.88093970 -1.66161621

H 0.57004277 -2.16223325 -2.40120257

#
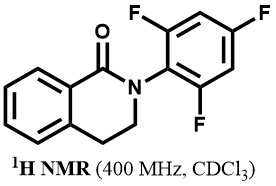

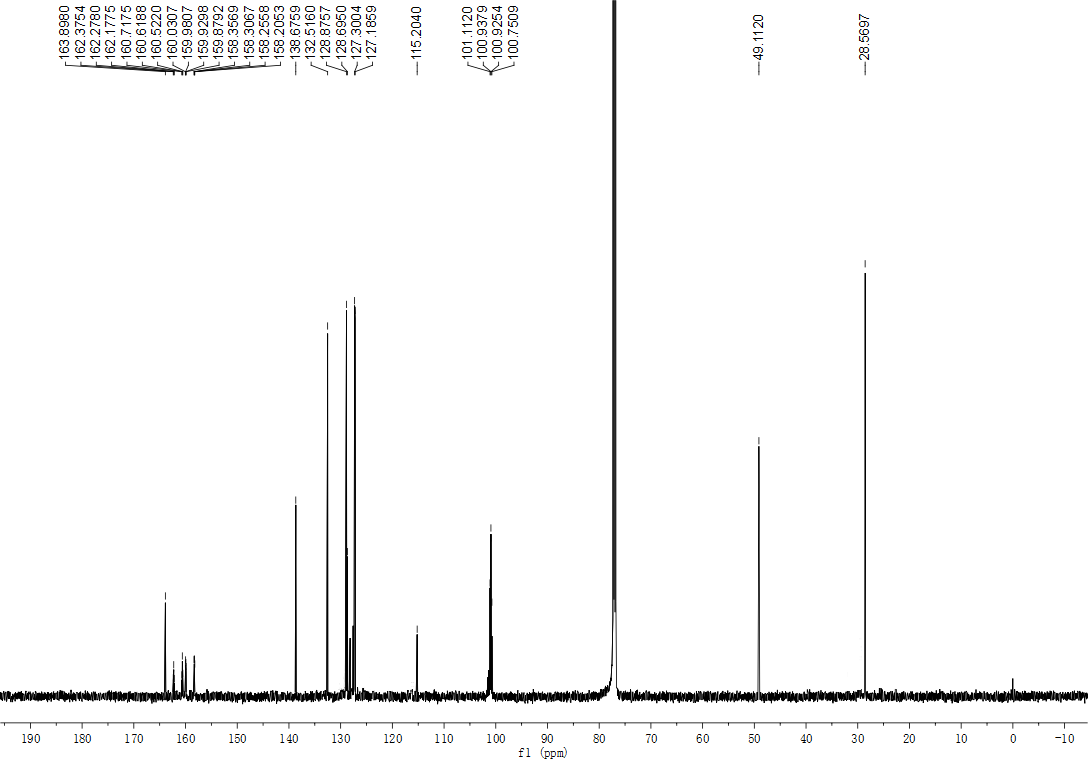

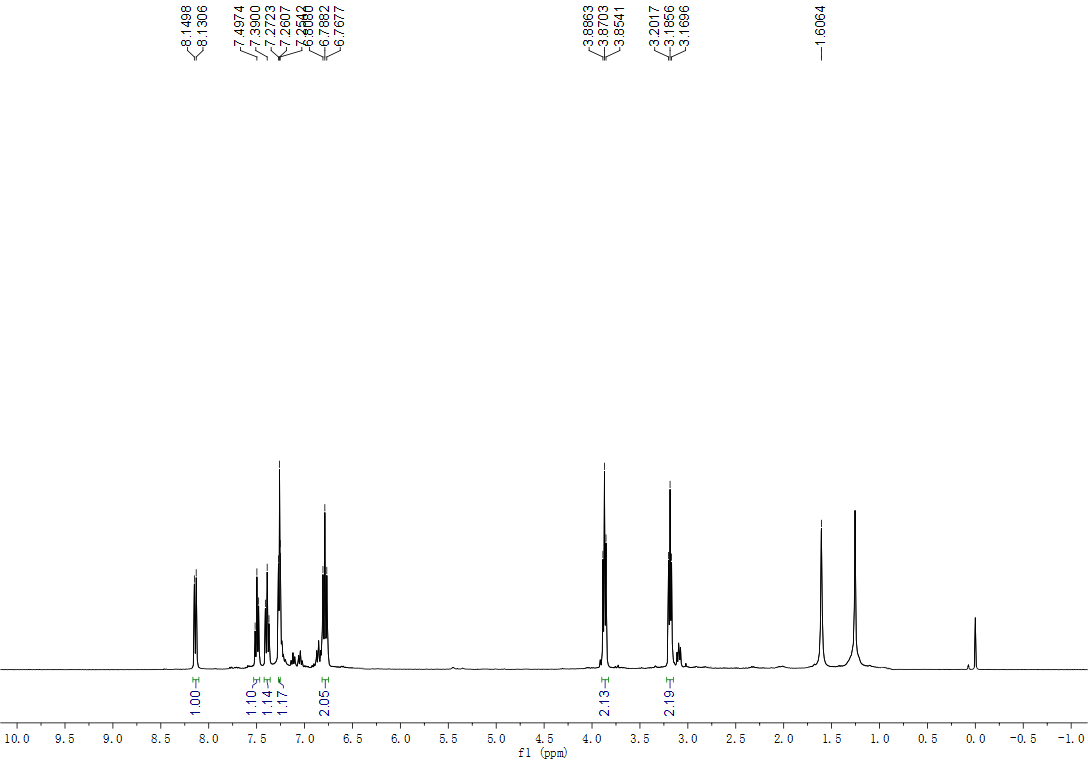
**7. Copies of product NMR spectra**


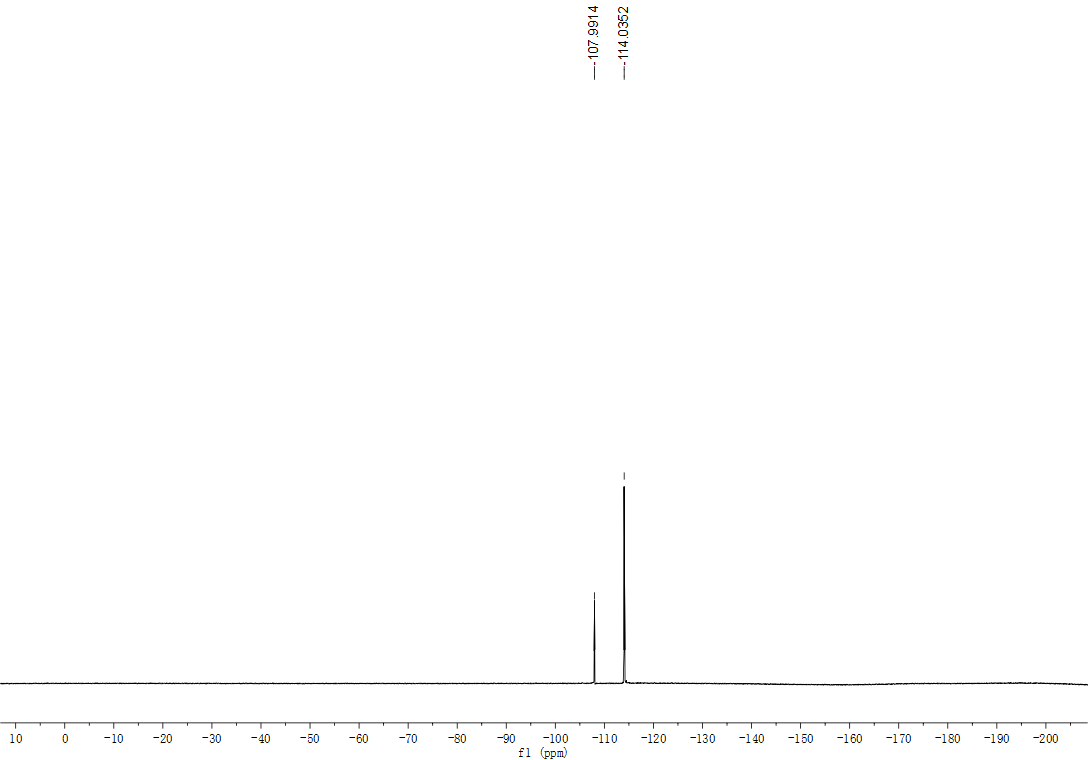


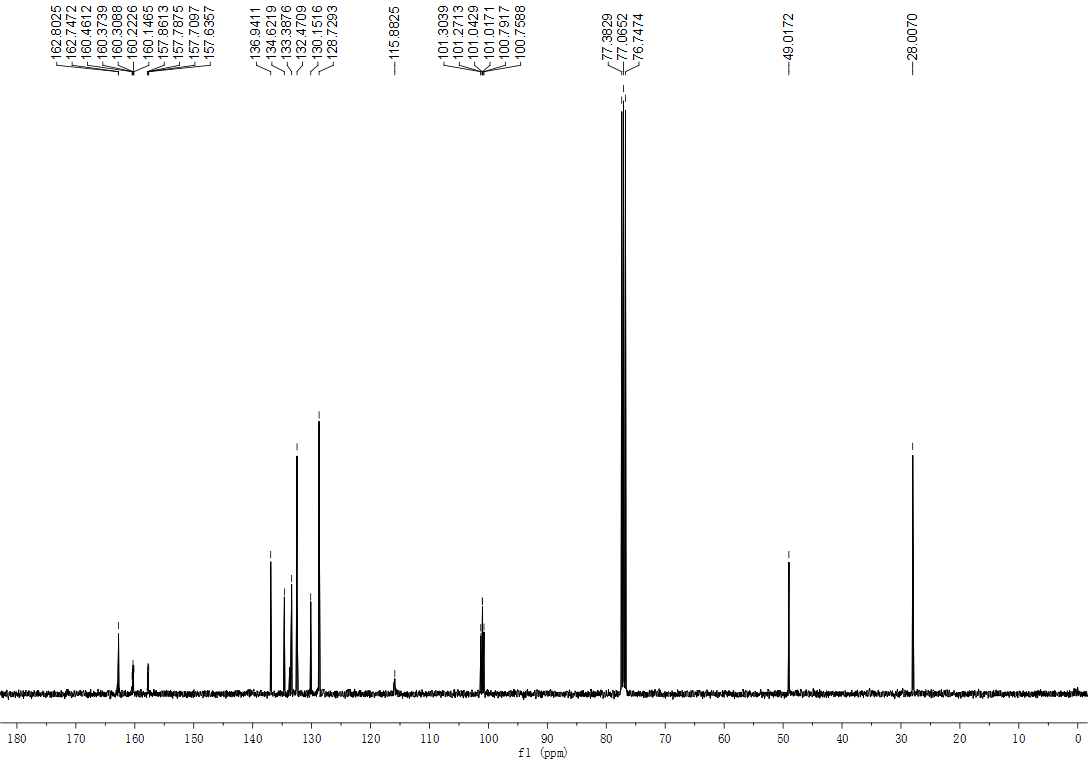

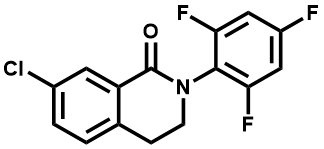

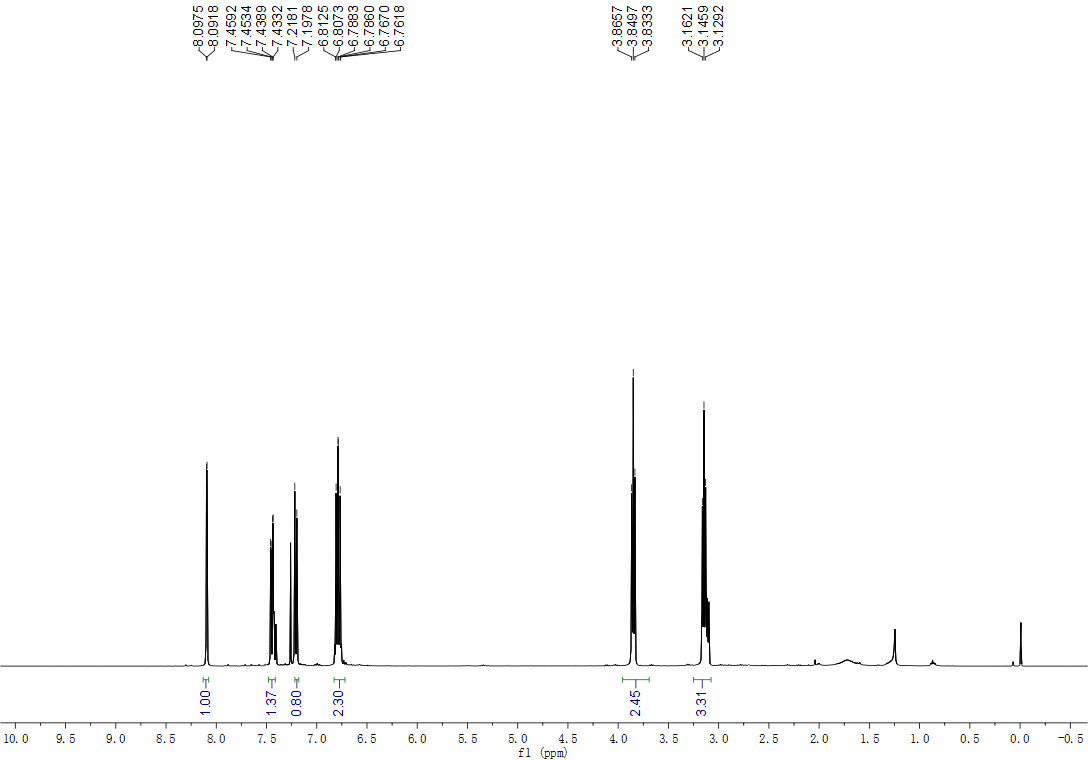


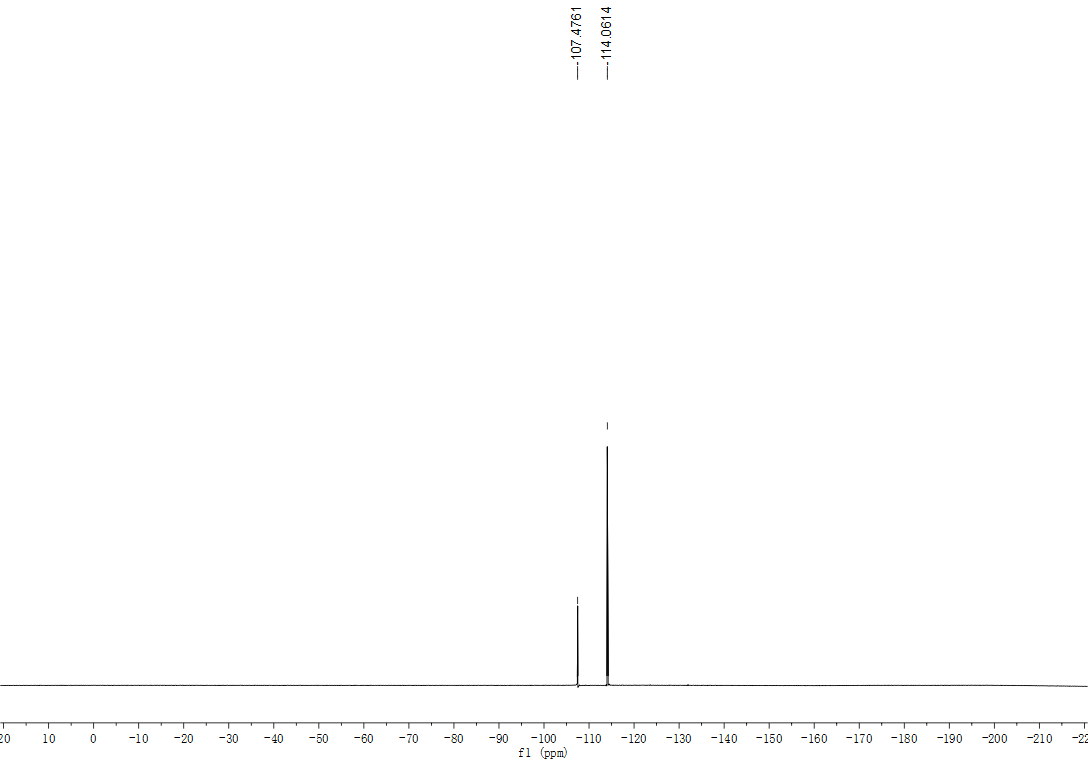


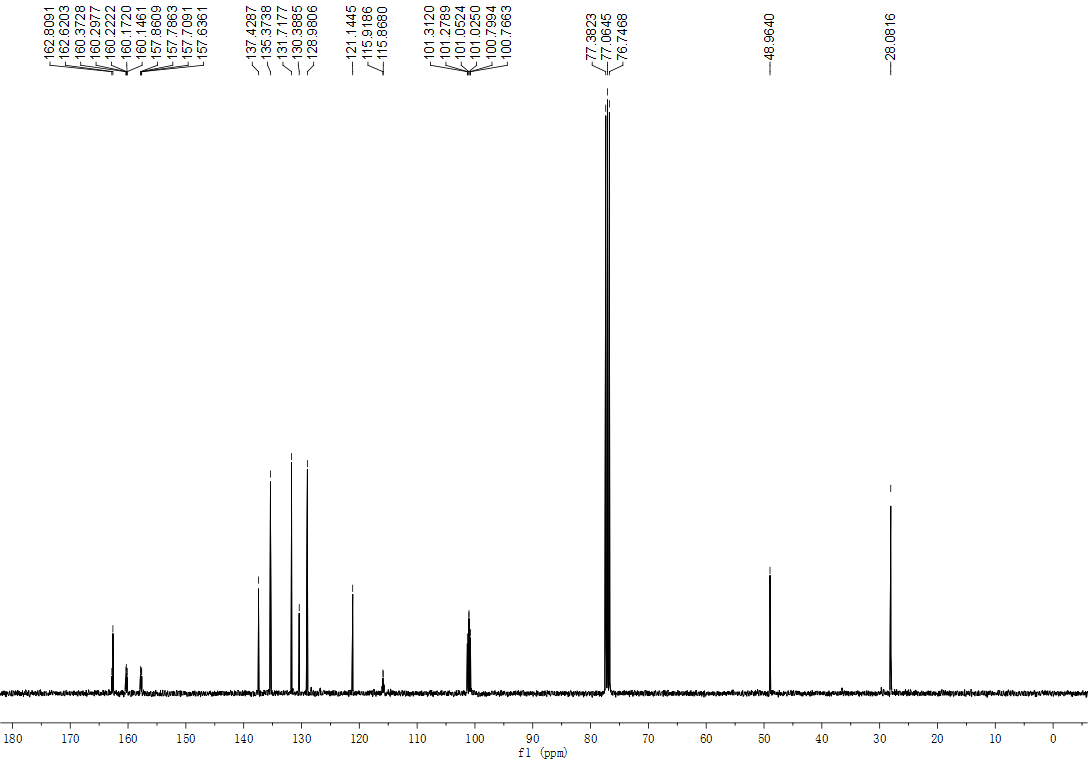

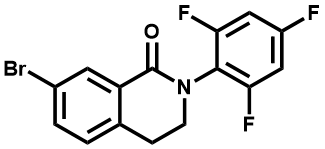

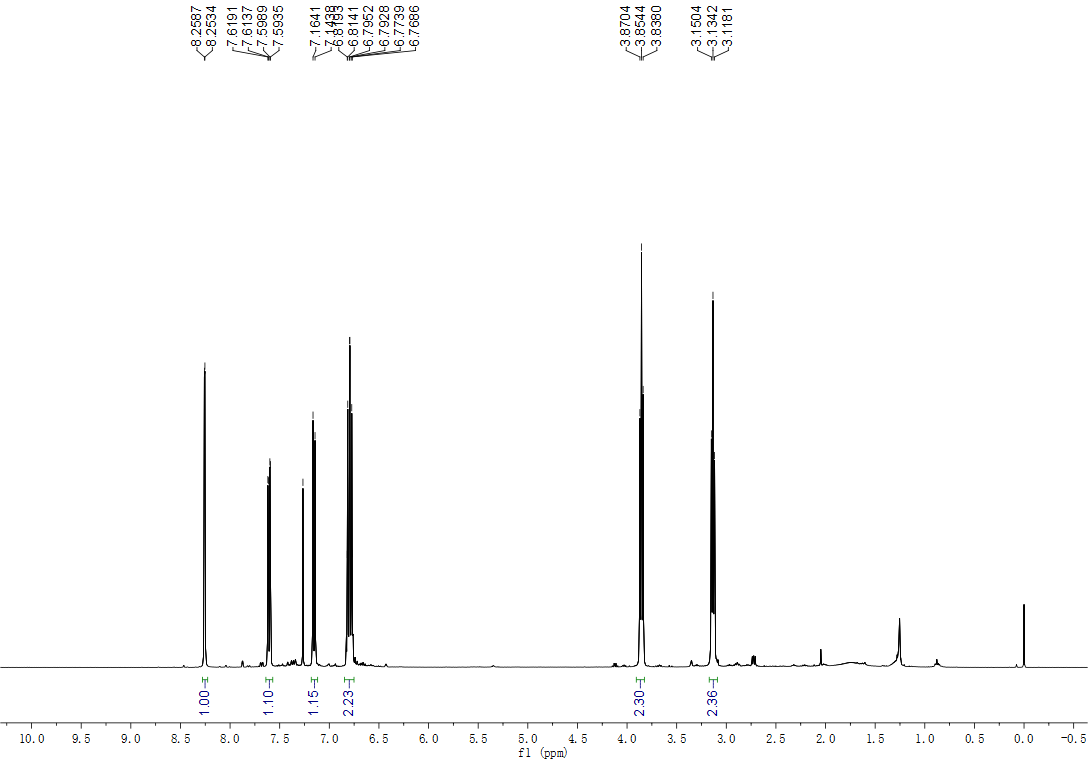


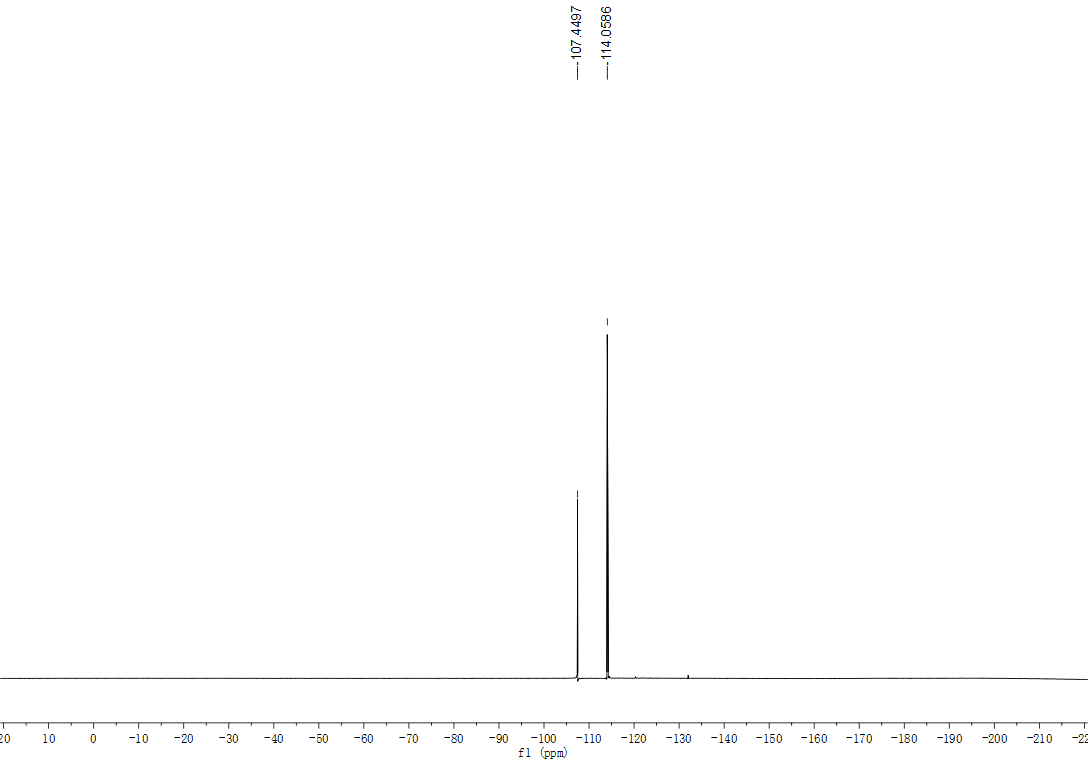


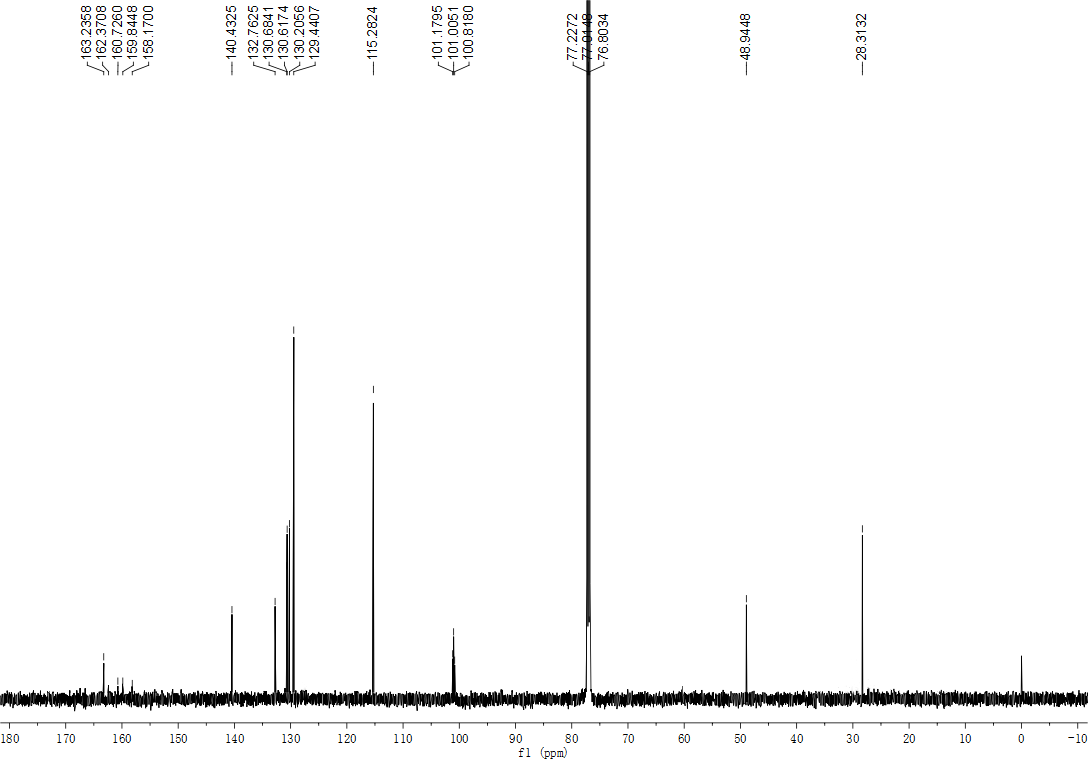

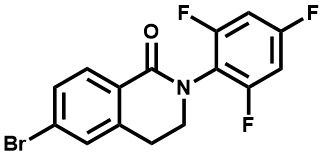

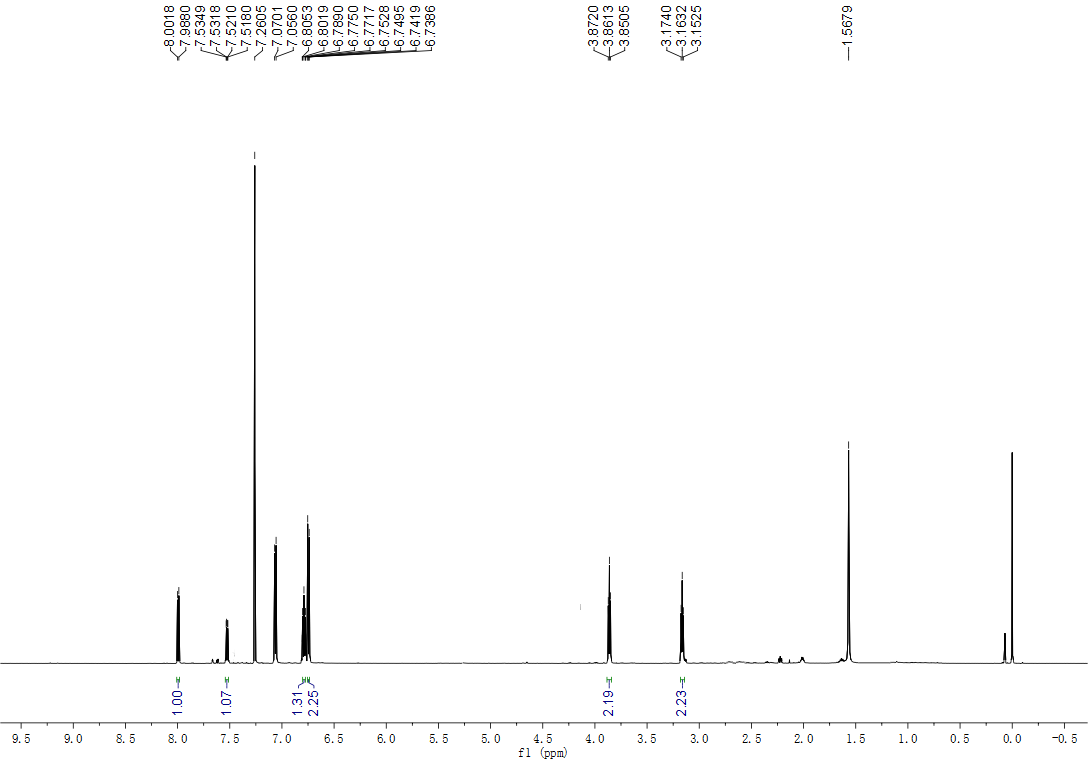


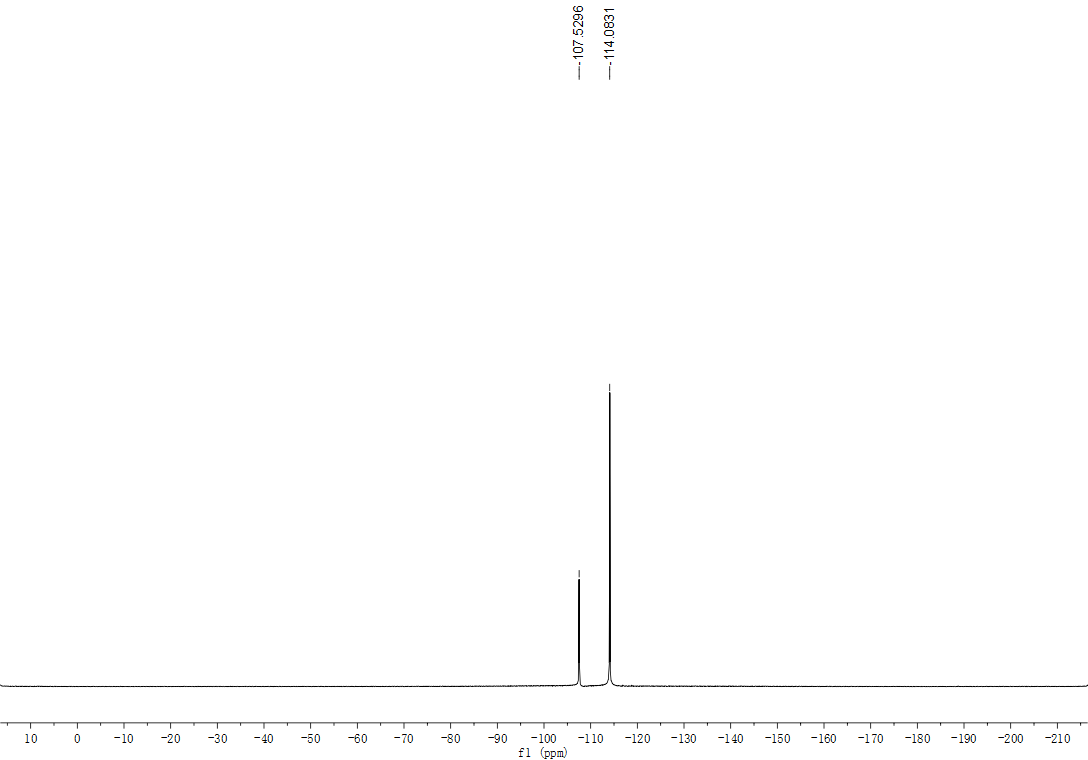


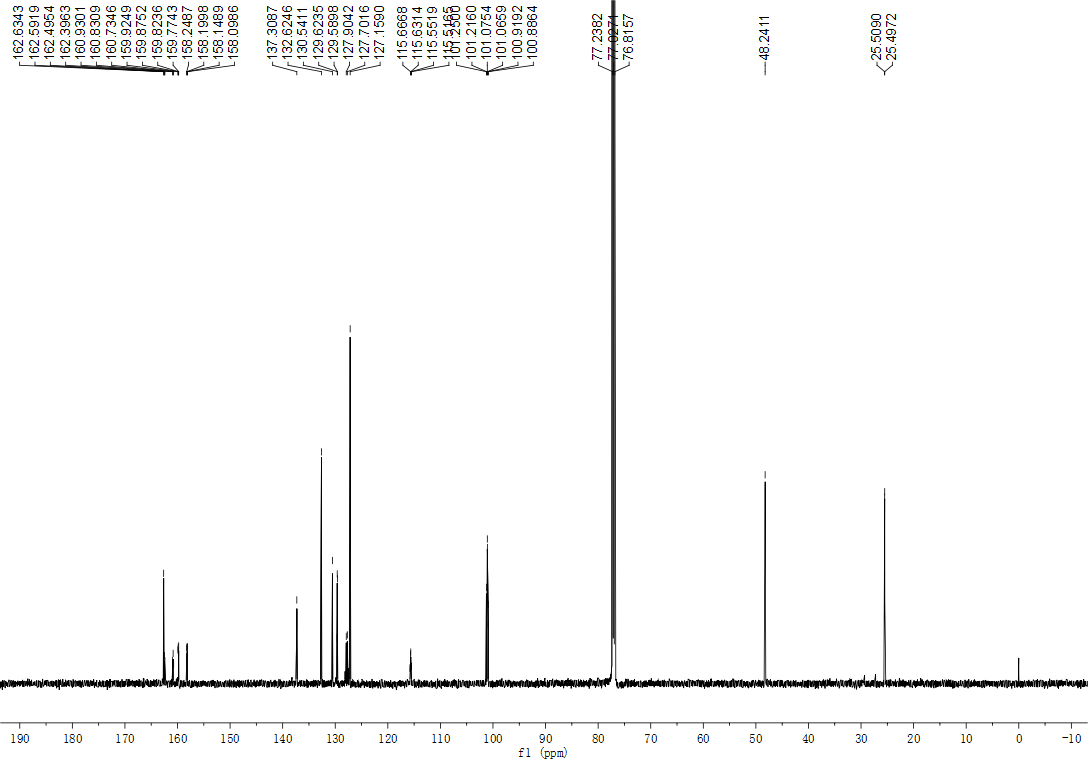

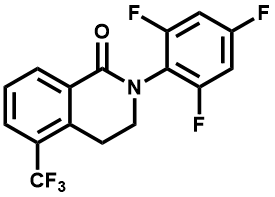

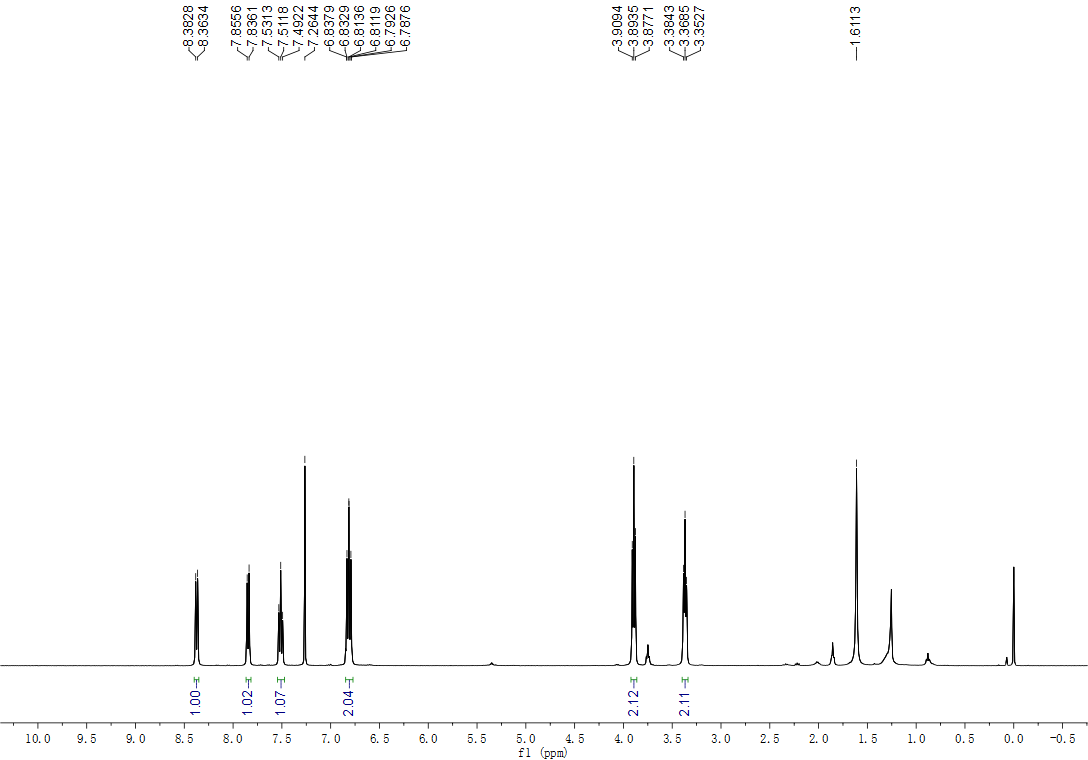


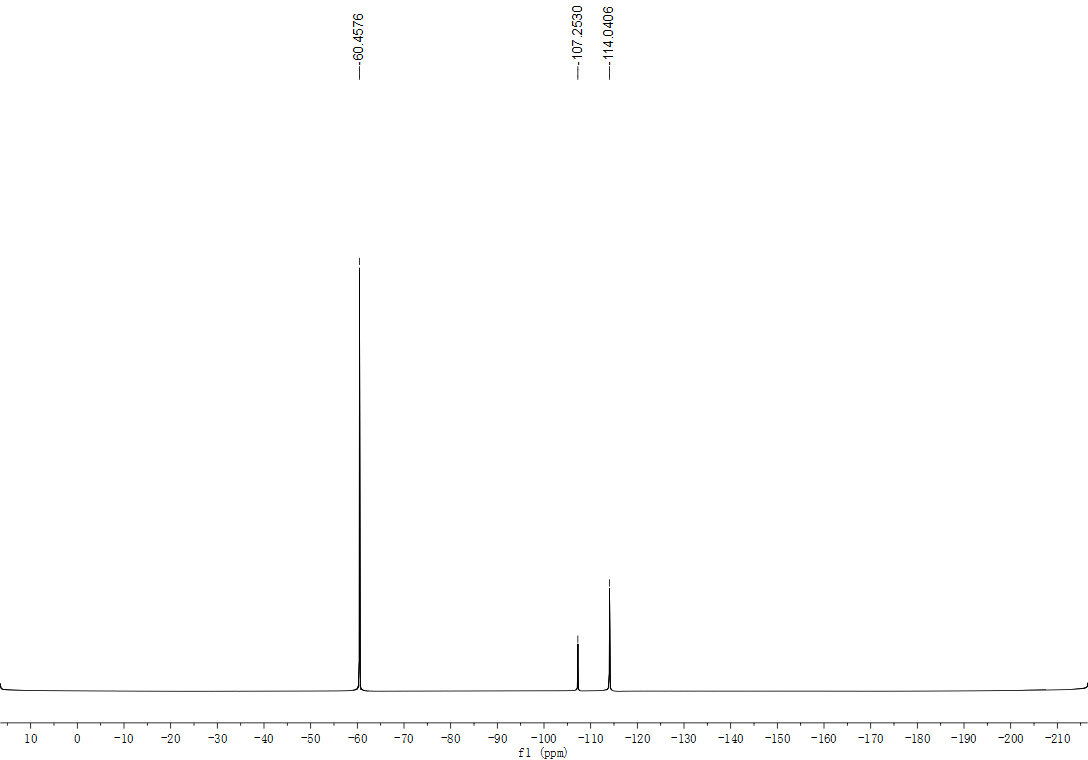


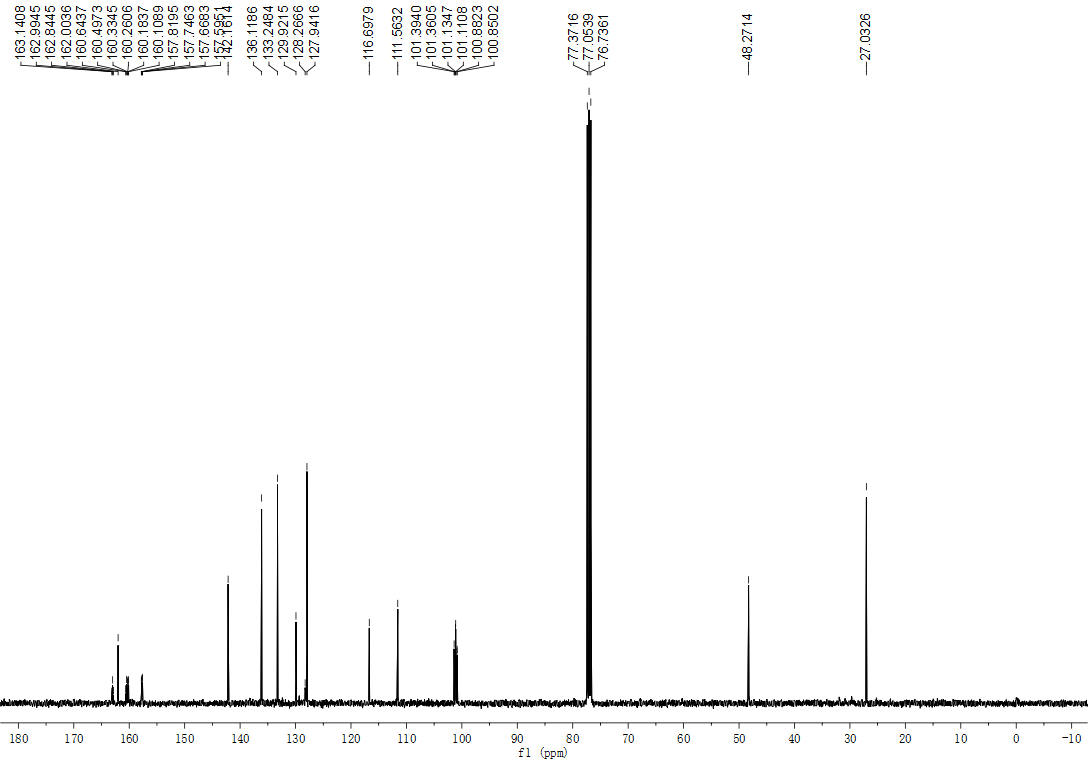

Supplement: Supplementary file 1 — Supporting Information [file ADVS-13-e18828-s001.docx]
